# Supplementary material for: The differential impact of pediatric COVID-19 between high-income countries and low- and middle-income countries: A systematic review of fatality and ICU admission in children worldwide
Source: PLoS One. 2021 Jan 29;16(1):e0246326. doi: 10.1371/journal.pone.0246326 (PMC7845974; doi:10.1371/journal.pone.0246326)
Supplement: S1 File — (DOCX) [file pone.0246326.s002.docx]

**Supplementary methods**

**Searching results for each database**

**Search 1 was performed on April 27, 2020**

**Search 2 was performed on Aug 10, 2020**

**Search 3 was performed on Dec 7, 2020**

|  | **Results** |
| --- | --- |
| **Search 1 Database [Platform]** Searches completed on April 30, 2020 |  |
| MEDLINE(R) and Epub Ahead of Print, In-Process & Other Non-Indexed Citations and Daily [OVID] 1946 to April 24, 2020 | 3266 |
| Embase Classic+Embase [OVID] 1947 to 2020 Week 17 | 2543 |
| Cochrane Central Register of Controlled Trials March 2020, EBM Reviews - Cochrane Database of Systematic Reviews [OVID] 2005 to April 22, 2020 | 110 |
| Cumulative Index of Nursing and Allied Health Plus (CINAHL Plus) [EBSCO]April 27, 2020 | 863 |
| WHO Covid-19 Database – Pediatrics April 29, 2020 | 635 |
| WHO Covid-19 Database – Outcomes April 29, 2020 | 5173 |
| Wanfang  (Chinese) Jan 1 to Apr 28, 2020 | 949 |
| CNKI (Chinese) Jan 1 to Apr 28, 2020 | 1542 |
| SciELO (Spanish/Portuguese) Jan 1 to Apr 27, 2020 | 114 |
| LiSSa (French) Jan 1 to Apr 27, 2020 | 65 |
| ICHUSHI (Japanese) Jan 1 to Apr 27, 2020 | 13 |
| KMbase (Korean) Jan 1 to Apr 27, 2020 | 88 |
| Magiran (Persian) Jan 27 to Apr 27, 2020 | 84 |
| TRdizin(Ulakbim) Jan 1 to Apr 27, 2020 | 1 |
| Islamic World Science Citation Center (Arabic) Jan 1 to Apr 30, 2020 | 1 |
| Russian Scientific Electronic Library (Russian) Jan 1 to Apr 27, 2020 | 223 |
| LILACS – Jan 1 to April 30, 2020 | 221 |
| **Subtotal** | **15891** |
| **Search 2 Database [Platform]** Searches run August 10, 2020 (rerun from April 27, 2020 |  |
| MEDLINE(R) and Epub Ahead of Print, In-Process & Other Non-Indexed Citations and Daily [OVID] 1946 to August 07, 2020 | 3023 |
| Embase Classic+Embase [OVID] 1947 to 2020 Week 32 | 3258 |
| **Subtotal** | **6281** |
| **Search 3 Database [Platform]** Searches run December 7, 2020 (updated from August 10, 2020) |  |
| MEDLINE(R) and Epub Ahead of Print, In-Process & Other Non-Indexed Citations and Daily [OVID] 1946 to December 04, 2020 | 2932 |
| Embase Classic+Embase [OVID] 1947 to 2020 Week 49 | 3451 |
| **Subtotal** | **6383** |
| Manually added articles | **2** |
| **Duplicates removed** | **-12530** |
| **Total** | **16027** |

**Search 1**

**Ovid MEDLINE(R) and Epub Ahead of Print, In-Process & Other Non-Indexed Citations and Daily**1946 to April 24, 2020

Search Strategy:

| **#** | **Searches** | **Results** |
| --- | --- | --- |
| 1 | exp Coronavirus/ | 12592 |
| 2 | exp Coronavirus Infections/ | 11016 |
| 3 | (coronavirus* or corona virus* or OC43 or NL63 or 229E or HKU1 or HCoV* or ncov* or covid* or sarscov* or sarscov* or Sars-coronavirus* or Severe Acute Respiratory Syndrome Coronavirus*).mp. | 21036 |
| 4 | (or/1-3) and (("201912" or 202*).dp. or 20191201:20301231.(ep).) [This set is the sensitive/broad part of the search with limits to date of publication or electronic publication to include 2019 to current] | 7633 |
| 5 | 4 not (SARS or SARS-CoV or MERS or MERS-CoV or Middle East respiratory syndrome or camel* or dromedar* or equine or coronary or coronal or covidence* or covidien or influenza virus or HIV or bovine or calves or TGEV or feline or porcine or erinaceus or BCoV or PED or PEDV or PDCoV or FIPV or FCoV or canine or CCov or zoonotic or avian influenza or H1N1 or H5N1 or H5N6 or IBV or murine corona*).mp. [line 5 removes the noise in the search results] | 5296 |
| 6 | ((pneumonia or covid* or coronavirus* or corona virus* or ncov* or 2019-ncov or sars*).mp. or exp pneumonia/) and Wuhan.mp. | 935 |
| 7 | (2019-ncov or ncov19 or ncov-19 or sars-cov2 or sars-cov-2 or sarscov2 or sarscov-2 or Sarscoronavirus2 or Sars-coronavirus-2 or coronavirus-19 or covid19 or covid-19 or covid 2019 or "2019-novel Cov" or ((novel or new or nouveau) adj2 (CoV on nCoV or covid or coronavirus* or corona virus or Pandemi*2)) or (coronavirus* and pneumonia)).mp. | 9192 |
| 8 | COVID-19.rx,px,ox. or severe acute respiratory syndrome coronavirus 2.os. | 1540 |
| 9 | ("32240632" or "32236488" or "32268021" or "32267941" or "32169616" or "32267649" or "32267499" or "32267344" or "32248853" or "32246156" or "32243118" or "32240583" or "32237674" or "32234725" or "32173381" or "32227595" or "32185863" or "32221979" or "32213260" or "32205350" or "32202721" or "32197097" or "32196032" or "32188729" or "32176889" or "32088947" or "32277065" or "32273472" or "32273444" or "32145185" or "31917786" or "32267384" or "32265186" or "32253187" or "32265567" or "32231286" or "32105468" or "32179788" or "32152361" or "32152148" or "32140676" or "32053580" or "32029604" or "32127714" or "32047315" or "32020111" or "32267950" or "32249952" or "32172715").ui. [Manually curated articles relevant to COVID-19 or SARS-CoV-2 where pandemic is synonym and relevant to this topic, to account for typographical error in article titles and for articles not completely or properly indexed] | 49 |
| 10 | or/6-9 [Lines 6 through 9 are specific to COVID-19 or closely related] | 9267 |
| 11 | 5 or 10 | 9681 |
| 12 | 11 and 20191201:20301231.(dt). | 7687 |
| 13 | exp child/ or exp "congenital, hereditary, and neonatal diseases and abnormalities"/ or exp infant/ or adolescent/ or exp pediatrics/ or child, abandoned/ or exp child, exceptional/ or child, orphaned/ or child, unwanted/ or minors/ or (pediatric* or paediatric* or child* or newborn* or congenital* or infan* or baby or babies or neonat* or pre-term or preterm* or premature birth* or NICU or preschool* or pre-school* or kindergarten* or kindergarden* or elementary school* or nursery school* or (day care* not adult*) or schoolchild* or toddler* or boy or boys or girl* or middle school* or pubescen* or juvenile* or teen* or youth* or high school* or adolesc* or pre-pubesc* or prepubesc*).mp. or (child* or adolesc* or pediat* or paediat*).jn. | 5032745 |
| 14 | Epidemiology/ | 12333 |
| 15 | exp Mortality/ | 377110 |
| 16 | exp "Severity of Illness Index"/ | 247741 |
| 17 | treatment outcome/ | 961008 |
| 18 | (epidemiolog* or death? or fatalit* or mortalit* or outcome? or sever*).tw,kf. | 5344147 |
| 19 | intensive care units/ or intensive care units, pediatric/ or intensive care units, neonatal/ | 75045 |
| 20 | (intensive care or ICU or ICUs or NICU or NICUs).tw,kf. | 166360 |
| 21 | exp Morbidity/ | 548158 |
| 22 | Prognosis/ | 500695 |
| 23 | (clinical or incidence? or morbidit* or prevalence? or prognos*).tw,kf. | 5012177 |
| 24 | or/13-23 | 11896705 |
| 25 | 12 and 24 | 3266 |

**Embase Classic+Embase**1947 to 2020 Week 17

Search Strategy:

| **#** | **Searches** | **Results** |
| --- | --- | --- |
| 1 | exp coronavirinae/ | 13276 |
| 2 | exp Coronavirus infection/ | 12285 |
| 3 | (coronavirus* or corona virus* or OC43 or NL63 or 229E or HKU1 or HCoV* or ncov* or covid* or sarscov* or sarscov* or Sars-coronavirus* or Severe Acute Respiratory Syndrome Coronavirus*).mp. | 29342 |
| 4 | 3 not (SARS or SARS-CoV or MERS or MERS-CoV or Middle East respiratory syndrome or camel* or dromedar* or equine or coronary or coronal or covidence* or covidien or influenza virus or HIV or bovine or calves or TGEV or feline or porcine or erinaceus or BCoV or PED or PEDV or PDCoV or FIPV or FCoV or canine or CCov or zoonotic or avian influenza or H1N1 or H5N1 or H5N6 or IBV or murine corona*).mp. [line 5 removes the noise in the search results] | 8408 |
| 5 | ((pneumonia or covid* or coronavirus* or corona virus* or ncov* or 2019-ncov or sars*).mp. or exp pneumonia/) and Wuhan.mp. | 760 |
| 6 | (2019-ncov or ncov19 or ncov-19 or sars-cov2 or sars-cov-2 or sarscov2 or sarscov-2 or Sarscoronavirus2 or Sars-coronavirus-2 or coronavirus-19 or covid19 or covid-19 or covid 2019 or "2019-novel Cov" or ((novel or new or nouveau) adj2 (CoV on nCoV or covid or coronavirus* or corona virus or Pandemi*2)) or (coronavirus* and pneumonia)).mp. | 7785 |
| 7 | or/4-6 | 12915 |
| 8 | exp child/ or exp "congenital, hereditary, and neonatal diseases and abnormalities"/ or exp infant/ or adolescent/ or exp pediatrics/ or child, abandoned/ or exp child, exceptional/ or child, orphaned/ or child, unwanted/ or minors/ or (pediatric* or paediatric* or child* or newborn* or congenital* or infan* or baby or babies or neonat* or pre-term or preterm* or premature birth* or NICU or preschool* or pre-school* or kindergarten* or kindergarden* or elementary school* or nursery school* or (day care* not adult*) or schoolchild* or toddler* or boy or boys or girl* or middle school* or pubescen* or juvenile* or teen* or youth* or high school* or adolesc* or pre-pubesc* or prepubesc*).mp. or (child* or adolesc* or pediat* or paediat*).jn. | 5883560 |
| 9 | epidemiology/ | 228336 |
| 10 | exp mortality/ | 1123022 |
| 11 | disease severity/ | 556299 |
| 12 | exp morbidity/ | 370674 |
| 13 | prognosis/ | 618400 |
| 14 | exp treatment outcome/ | 1633816 |
| 15 | intensive care unit/ or neonatal intensive care unit/ or pediatric intensive care unit/ | 167386 |
| 16 | (epidemiolog* or death? or fatalit* or mortalit* or outcome? or sever*).tw,kw. | 7554569 |
| 17 | (clinical or incidence? or morbidit* or prevalence? or prognos*).tw,kw. | 7407370 |
| 18 | (intensive care or ICU or ICUs or NICU or NICUs).tw,kw. | 276774 |
| 19 | or/8-18 | 15935174 |
| 20 | 7 and 19 | 6750 |
| 21 | limit 20 to yr="2019 -Current" | 2536 |
| 22 | ("20191201" or "20191202" or "20191203" or "20191204" or "20191205" or "20191206" or "20191208" or "20191209" or "20191210" or "20191211" or "20191212" or "20191213" or "20191214" or "20191215" or "20191216" or "20191217" or "20191218" or "20191219" or "20191220" or "20191221" or "20191222" or "20191223" or "20191224" or "20191225" or "20191226" or "20191227" or "20191228" or "20191229" or "20191230" or "20191231" or 202*).dc. | 910350 |
| 23 | 20 and 22 | 2382 |
| 24 | 21 or 23 | 2543 |

**Cochrane Central Register of Controlled Trials**March 2020**, EBM Reviews - Cochrane Database of Systematic Reviews**2005 to April 22, 2020

Search Strategy:

| **#** | **Searches** | **Results** |
| --- | --- | --- |
| 1 | exp Coronavirus/ | 10 |
| 2 | exp coronavirus infections/ | 70 |
| 3 | (coronavirus* or corona virus* or OC43 or NL63 or 229E or HKU1 or HCoV* or ncov* or covid* or sarscov* or sarscov* or Sars-coronavirus* or Severe Acute Respiratory Syndrome Coronavirus*).mp. | 1804 |
| 4 | 3 not (SARS or SARS-CoV or MERS or MERS-CoV or Middle East respiratory syndrome or camel* or dromedar* or equine or coronary or coronal or covidence* or covidien or influenza virus or HIV or bovine or calves or TGEV or feline or porcine or erinaceus or BCoV or PED or PEDV or PDCoV or FIPV or FCoV or canine or CCov or zoonotic or avian influenza or H1N1 or H5N1 or H5N6 or IBV or murine corona*).mp. [line 5 removes the noise in the search results] | 1020 |
| 5 | ((pneumonia or covid* or coronavirus* or corona virus* or ncov* or 2019-ncov or sars*).mp. or exp pneumonia/) and Wuhan.mp. | 24 |
| 6 | (2019-ncov or ncov19 or ncov-19 or sars-cov2 or sars-cov-2 or sarscov2 or sarscov-2 or Sarscoronavirus2 or Sars-coronavirus-2 or coronavirus-19 or covid19 or covid-19 or covid 2019 or "2019-novel Cov" or ((novel or new or nouveau) adj2 (CoV on nCoV or covid or coronavirus* or corona virus or Pandemi*2)) or (coronavirus* and pneumonia)).mp. | 115 |
| 7 | or/4-6 | 1092 |
| 8 | exp child/ or exp "congenital, hereditary, and neonatal diseases and abnormalities"/ or exp infant/ or adolescent/ or exp pediatrics/ or child, abandoned/ or exp child, exceptional/ or child, orphaned/ or child, unwanted/ or minors/ or (pediatric* or paediatric* or child* or newborn* or congenital* or infan* or baby or babies or neonat* or pre-term or preterm* or premature birth* or NICU or preschool* or pre-school* or kindergarten* or kindergarden* or elementary school* or nursery school* or (day care* not adult*) or schoolchild* or toddler* or boy or boys or girl* or middle school* or pubescen* or juvenile* or teen* or youth* or high school* or adolesc* or pre-pubesc* or prepubesc*).mp. or (child* or adolesc* or pediat* or paediat*).jn. | 310344 |
| 9 | Epidemiology/ | 14 |
| 10 | exp mortality/ | 12877 |
| 11 | "severity of illness index"/ | 19082 |
| 12 | morbidity/ | 743 |
| 13 | exp morbidity/ | 15027 |
| 14 | prognosis/ | 13732 |
| 15 | treatment outcome/ | 129616 |
| 16 | intensive care units/ or intensive care units, pediatric/ or intensive care units, neonatal/ | 3156 |
| 17 | (epidemiolog* or death? or fatalit* or mortalit* or outcome? or sever*).tw,kw. | 648924 |
| 18 | (clinical or incidence? or morbidit* or prevalence? or prognos*).tw,kw. | 814984 |
| 19 | (intensive care or ICU or ICUs or NICU or NICUs).tw,kw. | 29754 |
| 20 | or/8-19 | 1196729 |
| 21 | 7 and 20 | 742 |
| 22 | limit 21 to yr="2019 -Current" | 75 |
| 23 | ("201912" or "202001" or "202002" or "202003").up. | 464109 |
| 24 | 21 and 23 | 97 |
| 25 | 22 or 24 | 110 |

**CINAHL Plus**

**Cumulative Index of Nursing and Allied Health Literature [EBSCO]**

| Monday, April 27, 2020 12:07:18 PM |
| --- |

| **#** | **Query** | **Limiters/Expanders** | **Last Run Via** | **Results** |
| --- | --- | --- | --- | --- |
| S30 | S15 AND S28 | Limiters - Published Date: 20191201-20201231 Search modes - Boolean/Phrase | Interface - EBSCOhost Research Databases Search Screen - Advanced Search Database - CINAHL Plus with Full Text | 863 |
| S29 | S15 AND S28 | Search modes - Boolean/Phrase | Interface - EBSCOhost Research Databases Search Screen - Advanced Search Database - CINAHL Plus with Full Text | 1,816 |
| S28 | S17 OR S18 OR S19 OR S20 OR S21 OR S22 OR S23 OR S24 OR S25 OR S26 OR S27 | Search modes - Boolean/Phrase | Interface - EBSCOhost Research Databases Search Screen - Advanced Search Database - CINAHL Plus with Full Text | 4,151,891 |
| S27 | TX ("intensive care" or ICU or ICUs or NICU or NICUs) | Search modes - Boolean/Phrase | Interface - EBSCOhost Research Databases Search Screen - Advanced Search Database - CINAHL Plus with Full Text | 196,674 |
| S26 | TX (clinical or incidence* or morbidit* or prevalence* or prognos*) | Search modes - Boolean/Phrase | Interface - EBSCOhost Research Databases Search Screen - Advanced Search Database - CINAHL Plus with Full Text | 2,512,744 |
| S25 | TX (epidemiolog* or death* or fatalit* or mortalit* or outcome* or sever*) | Search modes - Boolean/Phrase | Interface - EBSCOhost Research Databases Search Screen - Advanced Search Database - CINAHL Plus with Full Text | 2,544,013 |
| S24 | (MH "Intensive Care Units") OR (MH "Intensive Care Units, Neonatal") OR (MH "Intensive Care Units, Pediatric") | Search modes - Boolean/Phrase | Interface - EBSCOhost Research Databases Search Screen - Advanced Search Database - CINAHL Plus with Full Text | 60,940 |
| S23 | (MH "Treatment Outcomes") | Search modes - Boolean/Phrase | Interface - EBSCOhost Research Databases Search Screen - Advanced Search Database - CINAHL Plus with Full Text | 357,304 |
| S22 | (MH "Prognosis") | Search modes - Boolean/Phrase | Interface - EBSCOhost Research Databases Search Screen - Advanced Search Database - CINAHL Plus with Full Text | 80,421 |
| S21 | (MH "Morbidity+") | Search modes - Boolean/Phrase | Interface - EBSCOhost Research Databases Search Screen - Advanced Search Database - CINAHL Plus with Full Text | 169,400 |
| S20 | (MH "Severity of Illness") | Search modes - Boolean/Phrase | Interface - EBSCOhost Research Databases Search Screen - Advanced Search Database - CINAHL Plus with Full Text | 32,300 |
| S19 | (MH "Mortality+") | Search modes - Boolean/Phrase | Interface - EBSCOhost Research Databases Search Screen - Advanced Search Database - CINAHL Plus with Full Text | 75,525 |
| S18 | (MH "Epidemiology") | Search modes - Boolean/Phrase | Interface - EBSCOhost Research Databases Search Screen - Advanced Search Database - CINAHL Plus with Full Text | 6,942 |
| S17 | (pediatric* or paediatric* or child* or newborn* or congenital* or infan* or baby or babies or neonat* or “pre-term” or preterm or “premature birth*” or NICU or preschool* or “pre-school*” or kindergarten* or “elementary school*” or “nursery school*” or schoolchild* or toddler* or boy or boys or girl* or “middle school*” or pubescen* or juvenile* or teen* or youth* or “high school*” or adolesc*or prepubesc* or “pre-pubesc*” or "(MH "Child+") OR (MH "Adolescence+") OR (MH "Minors (Legal)") or "(MH "Child Abuse, Sexual") OR (MH "Child Behavior Disorders+") OR (MH "Child, Medically Fragile") OR (MH "Child Day Care") OR (MH "Child Behavior+") OR (MH "Child Mortality") OR (MH "Child Passenger Safety") OR (MH "Child Development Disorders, Pervasive+") OR (MH "Child Custody") OR (MH "Child Abuse+") OR (MH "Child Nutritional Physiology+") OR (MH "Child Behavior Checklist") ) OR SO ( child* or pediatric* or paediatric* or adolescent ) | Search modes - Boolean/Phrase | Interface - EBSCOhost Research Databases Search Screen - Advanced Search Database - CINAHL Plus with Full Text | 1,186,741 |
| S16 | S6 OR S11 OR S12 OR S13 OR S14 | Limiters - Published Date: 20191001-20201231 Search modes - Boolean/Phrase | Interface - EBSCOhost Research Databases Search Screen - Advanced Search Database - CINAHL Plus with Full Text | 1,645 |
| S15 | S6 OR S11 OR S12 OR S13 OR S14 | Search modes - Boolean/Phrase | Interface - EBSCOhost Research Databases Search Screen - Advanced Search Database - CINAHL Plus with Full Text | 2,669 |
| S14 | TX coronavirus* and pneumonia | Search modes - Boolean/Phrase | Interface - EBSCOhost Research Databases Search Screen - Advanced Search Database - CINAHL Plus with Full Text | 693 |
| S13 | TX (novel or new or nouveau) N2 (CoV on nCoV or covid or coronavirus* or corona virus or Pandemi*2) | Search modes - Boolean/Phrase | Interface - EBSCOhost Research Databases Search Screen - Advanced Search Database - CINAHL Plus with Full Text | 617 |
| S12 | TX 2019-ncov or ncov19 or ncov-19 or sars-cov2 or sars-cov-2 or sarscov2 or sarscov-2 or Sarscoronavirus2 or Sars-coronavirus-2 or coronavirus-19 or covid19 or covid-19 or covid 2019 or "2019-novel Cov" | Search modes - Boolean/Phrase | Interface - EBSCOhost Research Databases Search Screen - Advanced Search Database - CINAHL Plus with Full Text | 1,279 |
| S11 | S9 AND S10 | Search modes - Boolean/Phrase | Interface - EBSCOhost Research Databases Search Screen - Advanced Search Database - CINAHL Plus with Full Text | 386 |
| S10 | TX Wuhan | Search modes - Boolean/Phrase | Interface - EBSCOhost Research Databases Search Screen - Advanced Search Database - CINAHL Plus with Full Text | 9,387 |
| S9 | S7 OR S8 | Search modes - Boolean/Phrase | Interface - EBSCOhost Research Databases Search Screen - Advanced Search Database - CINAHL Plus with Full Text | 71,961 |
| S8 | TX pneumonia or covid* or coronavirus* or corona virus* or ncov* or 2019-ncov or sars*) | Search modes - Boolean/Phrase | Interface - EBSCOhost Research Databases Search Screen - Advanced Search Database - CINAHL Plus with Full Text | 71,961 |
| S7 | (MH "Pneumonia") | Search modes - Boolean/Phrase | Interface - EBSCOhost Research Databases Search Screen - Advanced Search Database - CINAHL Plus with Full Text | 11,535 |
| S6 | S4 NOT S5 | Search modes - Boolean/Phrase | Interface - EBSCOhost Research Databases Search Screen - Advanced Search Database - CINAHL Plus with Full Text | 1,843 |
| S5 | TX SARS or SARS-CoV or MERS or MERS-CoV or Middle East respiratory syndrome or camel* or dromedar* or equine or coronary or coronal or covidence* or covidien or influenza virus or HIV or bovine or calves or TGEV or feline or porcine or erinaceus or BCoV or PED or PEDV or PDCoV or FIPV or FCoV or canine or CCov or zoonotic or avian influenza or H1N1 or H5N1 or H5N6 or IBV or murine corona* | Search modes - Boolean/Phrase | Interface - EBSCOhost Research Databases Search Screen - Advanced Search Database - CINAHL Plus with Full Text | 434,047 |
| S4 | S1 OR S2 OR S3 | Search modes - Boolean/Phrase | Interface - EBSCOhost Research Databases Search Screen - Advanced Search Database - CINAHL Plus with Full Text | 5,619 |
| S3 | TX (coronavirus* or corona virus* or OC43 or NL63 or 229E or HKU1 or HCoV* or ncov* or covid* or sarscov* or sarscov* or Sars-coronavirus* or Severe Acute Respiratory Syndrome Coronavirus*) | Search modes - Boolean/Phrase | Interface - EBSCOhost Research Databases Search Screen - Advanced Search Database - CINAHL Plus with Full Text | 5,619 |
| S2 | (MH "Coronavirus Infections") | Search modes - Boolean/Phrase | Interface - EBSCOhost Research Databases Search Screen - Advanced Search Database - CINAHL Plus with Full Text | 1,067 |
| S1 | (MH "Coronavirus") | Search modes - Boolean/Phrase | Interface - EBSCOhost Research Databases Search Screen - Advanced Search Database - CINAHL Plus with Full Text | 309 |

**WHO COVID-19 Database – April 29, 2020**

**Pediatric terms:**

"title, abstract, subject fields":

pediatric* or paediatric* or child* or newborn* or infan* or baby or babies or neonat* or pre-term or preterm* or premature birth* or NICU or pubescen* or juvenile* or teen* or youth* or adolesc* or pre-pubesc* or prepubesc*

Yield: 635

**Outcome terms:**

"title, abstract, subject fields":

clinical or death* or epidemiolog* or fatalit* or intensive care or ICU or ICUs or NICU or NICUs or morbidit* or mortalit* or outcome* or prognos* or sever*

Yield: 5173

**CNKI**

(to Apr 28, 2020)

1542 results

((AB=covid or AB=新冠病毒 or AB=武汉肺炎) and (AB=儿童 or AB=小儿 or AB=青少年 or AB=婴儿 or AB=新生儿 or AB=流行病 or AB=死亡 or AB=致命 or AB=重症监护 or AB=ICU or AB=严重 or AB=临床))OR ((TI=covid or TI=新冠病毒 or TI=武汉肺炎) and (TI=儿童 or TI=小儿 or TI=青少年 or TI=婴儿 or TI=新生儿 or TI=流行病 or TI=死亡 or TI=致命 or TI=重症监护 or TI=ICU or TI=严重 or TI=临床)) or ((KY=covid or KY=新冠病毒 or KY=武汉肺炎) and (KY=儿童 or KY=小儿 or KY=青少年 or KY=婴儿 or KY=新生儿 or KY=流行病 or KY=死亡 or KY=致命 or KY=重症监护 or KY=ICU or KY=严重 or KY=临床))

The same searching words by traditional Chinese found 25 fewer results (1517 results). Therefore, we extracted searching results with simplified Chinese

**Wanfang**

Full text search (Jan 1 to Apr 28, 2020)

949 results

(covid or新冠病毒 or武汉肺炎) and (儿童 or 小儿 or 青少年 or 婴儿 or 新生儿 or流行病 or 死亡 or 致命 or 重症监护 or ICU or 严重 or 临床)

The same searching words by traditional Chinese found the same number of results (949 results). Therefore, we extracted searching results with simplified Chinese

**LILACS**

(Jan 1 to Apr 30, 2020)

221 results

2020 Jan 1 - Apr30

(tw:((COVID-19 OR SARS OR coronavirus) )) AND (tw:((pediatric OR pediatrics OR paediatrics OR paediatric OR children OR infants OR adolescents OR outcomes OR epidemiology OR morbidity OR mortality OR death OR fatalities OR clinical OR prognosis)))

**SciELO**

Jan 1 to Apr 27, 2020

114 results

COVID-19 OR SARS OR coronavirus OR coronavírus

**LiSSa**

Jan 1 to Apr 27, 2020

114 results

COVID-19 OR SARS OR coronavirus

**ICHUSHI Web**

Jan 1 to Apr 27, 2020

13 results

COVID-19 OR SARS OR coronavirus OR コロナウイルス

**KMbase**

Jan 1 to Apr 27, 2020

88 results

COVID-19 OR SARS OR coronavirus OR 코로나바이러스

**Magiran**

Jan 27 to Apr 27, 2020

84 results

COVID-19 OR SARS OR coronavirus OR ویروس کرونا

**TRdizin(Ulakbim)**

Jan 1 to Apr 27, 2020

1 result

COVID-19 OR SARS OR coronavirus OR koronavirüs

**Islamic World Science Citation Center**

Jan 27 to Apr 30, 2020

1 result

COVID-19 OR SARS OR coronavirus OR فيروس كورونا

**Russian: Scientific electronic library (elibrary.ru)**

Jan 1 to Apr 27, 2020

223 results

COVID-19 OR SARS OR coronavirus OR коронавирус

**Search 2 (completed on Aug 10, 2020)**

**Ovid MEDLINE(R) and Epub Ahead of Print, In-Process & Other Non-Indexed Citations and Daily**1946 to August 07, 2020
Search Strategy:

| **#** | **Searches** | **Results** |
| --- | --- | --- |
| 1 | exp Coronavirus/ | 25240 |
| 2 | exp Coronavirus Infections/ | 26114 |
| 3 | (coronavirus* or corona virus* or OC43 or NL63 or 229E or HKU1 or HCoV* or ncov* or covid* or sarscov* or sarscov* or Sars-coronavirus* or Severe Acute Respiratory Syndrome Coronavirus*).mp. | 53987 |
| 4 | (or/1-3) and (("201912" or 202*).dp. or 20191201:20301231.(ep).) [This set is the sensitive/broad part of the search with limits to date of publication or electronic publication to include 2019 to current] | 40555 |
| 5 | 4 not (SARS or SARS-CoV or MERS or MERS-CoV or Middle East respiratory syndrome or camel* or dromedar* or equine or coronary or coronal or covidence* or covidien or influenza virus or HIV or bovine or calves or TGEV or feline or porcine or erinaceus or BCoV or PED or PEDV or PDCoV or FIPV or FCoV or canine or CCov or zoonotic or avian influenza or H1N1 or H5N1 or H5N6 or IBV or murine corona*).mp. [line 5 removes the noise in the search results] | 27430 |
| 6 | ((pneumonia or covid* or coronavirus* or corona virus* or ncov* or 2019-ncov or sars*).mp. or exp pneumonia/) and Wuhan.mp. | 2376 |
| 7 | (2019-ncov or ncov19 or ncov-19 or sars-cov2 or sars-cov-2 or sarscov2 or sarscov-2 or Sarscoronavirus2 or Sars-coronavirus-2 or coronavirus-19 or covid19 or covid-19 or covid 2019 or "2019-novel Cov" or ((novel or new or nouveau) adj2 (CoV on nCoV or covid or coronavirus* or corona virus or Pandemi*2)) or (coronavirus* and pneumonia)).mp. | 41616 |
| 8 | COVID-19.rx,px,ox. or severe acute respiratory syndrome coronavirus 2.os. | 16527 |
| 9 | ("32240632" or "32236488" or "32268021" or "32267941" or "32169616" or "32267649" or "32267499" or "32267344" or "32248853" or "32246156" or "32243118" or "32240583" or "32237674" or "32234725" or "32173381" or "32227595" or "32185863" or "32221979" or "32213260" or "32205350" or "32202721" or "32197097" or "32196032" or "32188729" or "32176889" or "32088947" or "32277065" or "32273472" or "32273444" or "32145185" or "31917786" or "32267384" or "32265186" or "32253187" or "32265567" or "32231286" or "32105468" or "32179788" or "32152361" or "32152148" or "32140676" or "32053580" or "32029604" or "32127714" or "32047315" or "32020111" or "32267950" or "32249952" or "32172715").ui. [Manually curated articles relevant to COVID-19 or SARS-CoV-2 where pandemic is synonym and relevant to this topic, to account for typographical error in article titles and for articles not completely or properly indexed] | 49 |
| 10 | or/6-9 [Lines 6 through 9 are specific to COVID-19 or closely related] | 41688 |
| 11 | 5 or 10 | 43125 |
| 12 | 11 and 20191201:20301231.(dt). | 41124 |
| 13 | exp child/ or exp "congenital, hereditary, and neonatal diseases and abnormalities"/ or exp infant/ or adolescent/ or exp pediatrics/ or child, abandoned/ or exp child, exceptional/ or child, orphaned/ or child, unwanted/ or minors/ or (pediatric* or paediatric* or child* or newborn* or congenital* or infan* or baby or babies or neonat* or pre-term or preterm* or premature birth* or NICU or preschool* or pre-school* or kindergarten* or kindergarden* or elementary school* or nursery school* or (day care* not adult*) or schoolchild* or toddler* or boy or boys or girl* or middle school* or pubescen* or juvenile* or teen* or youth* or high school* or adolesc* or pre-pubesc* or prepubesc*).mp. or (child* or adolesc* or pediat* or paediat*).jn. | 5092485 |
| 14 | Epidemiology/ | 12368 |
| 15 | exp Mortality/ | 382742 |
| 16 | exp "Severity of Illness Index"/ | 251751 |
| 17 | treatment outcome/ | 976747 |
| 18 | (epidemiolog* or death? or fatalit* or mortalit* or outcome? or sever*).tw,kf. | 5466105 |
| 19 | intensive care units/ or intensive care units, pediatric/ or intensive care units, neonatal/ | 76898 |
| 20 | (intensive care or ICU or ICUs or NICU or NICUs).tw,kf. | 171684 |
| 21 | exp Morbidity/ | 558108 |
| 22 | Prognosis/ | 509357 |
| 23 | (clinical or incidence? or morbidit* or prevalence? or prognos*).tw,kf. | 5119664 |
| 24 | or/13-23 | 12105244 |
| 25 | 12 and 24 | 20057 |
| 26 | 12 and 13 | 3322 |
| 27 | ("20200425" or "20200426" or "20200427" or "20200428" or "20200429" or "20200430" or 202005* or 202006* or 202007* or 202008* or 202009* or 202010* or 202011* or 202012*).dt,ez,da. | 648636 |
| 28 | 26 and 27 | 3023 |

**Embase Classic+Embase**1947 to 2020 Week 32
Search Strategy:

| **#** | **Searches** | **Results** |
| --- | --- | --- |
| 1 | exp coronavirinae/ | 17356 |
| 2 | exp Coronavirus infection/ | 17964 |
| 3 | (coronavirus* or corona virus* or OC43 or NL63 or 229E or HKU1 or HCoV* or ncov* or covid* or sarscov* or sarscov* or Sars-coronavirus* or Severe Acute Respiratory Syndrome Coronavirus*).mp. | 65338 |
| 4 | (or/1-3) and 20190101:20301231.(dc). | 43884 |
| 5 | 4 not (SARS or SARS-CoV or MERS or MERS-CoV or Middle East respiratory syndrome or camel* or dromedar* or equine or coronary or coronal or covidence* or covidien or influenza virus or HIV or bovine or calves or TGEV or feline or porcine or erinaceus or BCoV or PED or PEDV or PDCoV or FIPV or FCoV or canine or CCov or zoonotic or avian influenza or H1N1 or H5N1 or H5N6 or IBV or murine corona*).mp. [line 5 removes the noise in the search results] | 27624 |
| 6 | ((pneumonia or covid* or coronavirus* or corona virus* or ncov* or 2019-ncov or sars*).mp. or exp pneumonia/) and Wuhan.mp. | 2436 |
| 7 | (2019-ncov or ncov19 or ncov-19 or sars-cov2 or sars-cov-2 or sarscov2 or sarscov-2 or Sarscoronavirus2 or Sars-coronavirus-2 or coronavirus-19 or covid19 or covid-19 or covid 2019 or "2019-novel Cov" or ((novel or new or nouveau) adj2 (CoV on nCoV or covid or coronavirus* or corona virus or Pandemi*2)) or (coronavirus* and pneumonia)).mp. | 41198 |
| 8 | or/5-7 | 44219 |
| 9 | exp child/ or exp "congenital, hereditary, and neonatal diseases and abnormalities"/ or exp infant/ or adolescent/ or exp pediatrics/ or child, abandoned/ or exp child, exceptional/ or child, orphaned/ or child, unwanted/ or minors/ or (pediatric* or paediatric* or child* or newborn* or congenital* or infan* or baby or babies or neonat* or pre-term or preterm* or premature birth* or NICU or preschool* or pre-school* or kindergarten* or kindergarden* or elementary school* or nursery school* or (day care* not adult*) or schoolchild* or toddler* or boy or boys or girl* or middle school* or pubescen* or juvenile* or teen* or youth* or high school* or adolesc* or pre-pubesc* or prepubesc*).mp. or (child* or adolesc* or pediat* or paediat*).jn. | 5959936 |
| 10 | epidemiology/ | 229474 |
| 11 | exp mortality/ | 1147282 |
| 12 | disease severity/ | 566687 |
| 13 | exp morbidity/ | 376907 |
| 14 | prognosis/ | 626951 |
| 15 | exp treatment outcome/ | 1671978 |
| 16 | intensive care unit/ or neonatal intensive care unit/ or pediatric intensive care unit/ | 174021 |
| 17 | (epidemiolog* or death? or fatalit* or mortalit* or outcome? or sever*).tw,kw. | 7718917 |
| 18 | (clinical or incidence? or morbidit* or prevalence? or prognos*).tw,kw. | 7565238 |
| 19 | (intensive care or ICU or ICUs or NICU or NICUs).tw,kw. | 285450 |
| 20 | or/9-19 | 16215190 |
| 21 | 8 and 20 | 24044 |
| 22 | limit 21 to yr="2019 -Current" | 21638 |
| 23 | ("20191201" or "20191202" or "20191203" or "20191204" or "20191205" or "20191206" or "20191208" or "20191209" or "20191210" or "20191211" or "20191212" or "20191213" or "20191214" or "20191215" or "20191216" or "20191217" or "20191218" or "20191219" or "20191220" or "20191221" or "20191222" or "20191223" or "20191224" or "20191225" or "20191226" or "20191227" or "20191228" or "20191229" or "20191230" or "20191231" or 202*).dc. | 1508541 |
| 24 | 21 and 23 | 21451 |
| 25 | 22 or 24 | 21653 |
| 26 | 8 and 9 | 4264 |
| 27 | limit 26 to dc=20200427-20201231 | 3258 |

**Search 3 (completed on Dec 7, 2020)**

MEDLINE(R) and Epub Ahead of Print, In-Process & Other Non-Indexed Citations and Daily 1946 to December 04, 2020
Search Strategy:

| **#** | **Searches** | **Results** |
| --- | --- | --- |
| 1 | exp Coronavirus/ | 45017 |
| 2 | exp Coronavirus Infections/ | 49294 |
| 3 | (coronavirus* or corona virus* or OC43 or NL63 or 229E or HKU1 or HCoV* or ncov* or covid* or sarscov* or sarscov* or Sars-coronavirus* or Severe Acute Respiratory Syndrome Coronavirus*).mp. | 93303 |
| 4 | (or/1-3) and (("201912" or 202*).dp. or 20191201:20301231.(ep).) [This set is the sensitive/broad part of the search with limits to date of publication or electronic publication to include 2019 to current] | 79899 |
| 5 | 4 not (SARS or SARS-CoV or MERS or MERS-CoV or Middle East respiratory syndrome or camel* or dromedar* or equine or coronary or coronal or covidence* or covidien or influenza virus or HIV or bovine or calves or TGEV or feline or porcine or erinaceus or BCoV or PED or PEDV or PDCoV or FIPV or FCoV or canine or CCov or zoonotic or avian influenza or H1N1 or H5N1 or H5N6 or IBV or murine corona*).mp. [line 5 removes the noise in the search results] | 52803 |
| 6 | ((pneumonia or covid* or coronavirus* or corona virus* or ncov* or 2019-ncov or sars*).mp. or exp pneumonia/) and Wuhan.mp. | 3774 |
| 7 | (2019-ncov or ncov19 or ncov-19 or sars-cov2 or sars-cov-2 or sarscov2 or sarscov-2 or Sarscoronavirus2 or Sars-coronavirus-2 or coronavirus-19 or covid19 or covid-19 or covid 2019 or "2019-novel Cov" or ((novel or new or nouveau) adj2 (CoV on nCoV or covid or coronavirus* or corona virus or Pandemi*2)) or (coronavirus* and pneumonia)).mp. | 80767 |
| 8 | COVID-19.rx,px,ox. or severe acute respiratory syndrome coronavirus 2.os. | 39417 |
| 9 | ("32240632" or "32236488" or "32268021" or "32267941" or "32169616" or "32267649" or "32267499" or "32267344" or "32248853" or "32246156" or "32243118" or "32240583" or "32237674" or "32234725" or "32173381" or "32227595" or "32185863" or "32221979" or "32213260" or "32205350" or "32202721" or "32197097" or "32196032" or "32188729" or "32176889" or "32088947" or "32277065" or "32273472" or "32273444" or "32145185" or "31917786" or "32267384" or "32265186" or "32253187" or "32265567" or "32231286" or "32105468" or "32179788" or "32152361" or "32152148" or "32140676" or "32053580" or "32029604" or "32127714" or "32047315" or "32020111" or "32267950" or "32249952" or "32172715").ui. [Manually curated articles relevant to COVID-19 or SARS-CoV-2 where pandemic is synonym and relevant to this topic, to account for typographical error in article titles and for articles not completely or properly indexed] | 49 |
| 10 | or/6-9 [Lines 6 through 9 are specific to COVID-19 or closely related] | 80844 |
| 11 | 5 or 10 | 82760 |
| 12 | 11 and 20191201:20301231.(dt). | 80761 |
| 13 | exp child/ or exp "congenital, hereditary, and neonatal diseases and abnormalities"/ or exp infant/ or adolescent/ or exp pediatrics/ or child, abandoned/ or exp child, exceptional/ or child, orphaned/ or child, unwanted/ or minors/ or (pediatric* or paediatric* or child* or newborn* or congenital* or infan* or baby or babies or neonat* or pre-term or preterm* or premature birth* or NICU or preschool* or pre-school* or kindergarten* or kindergarden* or elementary school* or nursery school* or (day care* not adult*) or schoolchild* or toddler* or boy or boys or girl* or middle school* or pubescen* or juvenile* or teen* or youth* or high school* or adolesc* or pre-pubesc* or prepubesc*).mp. or (child* or adolesc* or pediat* or paediat*).jn. | 5162738 |
| 14 | Epidemiology/ | 12415 |
| 15 | exp Mortality/ | 389506 |
| 16 | exp "Severity of Illness Index"/ | 256618 |
| 17 | treatment outcome/ | 996128 |
| 18 | (epidemiolog* or death? or fatalit* or mortalit* or outcome? or sever*).tw,kf. | 5610081 |
| 19 | intensive care units/ or intensive care units, pediatric/ or intensive care units, neonatal/ | 79005 |
| 20 | (intensive care or ICU or ICUs or NICU or NICUs).tw,kf. | 178179 |
| 21 | exp Morbidity/ | 569437 |
| 22 | Prognosis/ | 518913 |
| 23 | (clinical or incidence? or morbidit* or prevalence? or prognos*).tw,kf. | 5247421 |
| 24 | or/14-23 | 9424285 |
| 25 | 12 and 13 and 24 | 4577 |
| 26 | ("20200810" or "20200811" or "20200812" or "20200813" or "20200814" or "20200815" or "20200816" or "20200817" or "20200818" or "20200819" or "20200820" or "20200821" or "20200822" or "20200823" or "20200824" or "20200825" or "20200826" or "20200827" or "20200828" or "20200829" or "20200830" or "20200831").dt,ez,da. | 136461 |
| 27 | (202009* or 202010* or 202011* or 202012*).dt,ez,da. | 585520 |
| 28 | or/26-27 | 711409 |
| 29 | 25 and 28 | 2932 |

**Embase Classic+Embase**1947 to 2020 Week 49
Search Strategy:

| **#** | **Searches** | **Results** |
| --- | --- | --- |
| 1 | exp coronavirinae/ | 22623 |
| 2 | exp Coronavirus infection/ | 24174 |
| 3 | (coronavirus* or corona virus* or OC43 or NL63 or 229E or HKU1 or HCoV* or ncov* or covid* or sarscov* or sarscov* or Sars-coronavirus* or Severe Acute Respiratory Syndrome Coronavirus*).mp. | 105404 |
| 4 | (or/1-3) and 20190101:20301231.(dc). | 83977 |
| 5 | 4 not (SARS or SARS-CoV or MERS or MERS-CoV or Middle East respiratory syndrome or camel* or dromedar* or equine or coronary or coronal or covidence* or covidien or influenza virus or HIV or bovine or calves or TGEV or feline or porcine or erinaceus or BCoV or PED or PEDV or PDCoV or FIPV or FCoV or canine or CCov or zoonotic or avian influenza or H1N1 or H5N1 or H5N6 or IBV or murine corona*).mp. [line 5 removes the noise in the search results] | 52736 |
| 6 | ((pneumonia or covid* or coronavirus* or corona virus* or ncov* or 2019-ncov or sars*).mp. or exp pneumonia/) and Wuhan.mp. | 3956 |
| 7 | (2019-ncov or ncov19 or ncov-19 or sars-cov2 or sars-cov-2 or sarscov2 or sarscov-2 or Sarscoronavirus2 or Sars-coronavirus-2 or coronavirus-19 or covid19 or covid-19 or covid 2019 or "2019-novel Cov" or ((novel or new or nouveau) adj2 (CoV on nCoV or covid or coronavirus* or corona virus or Pandemi*2)) or (coronavirus* and pneumonia)).mp. | 77589 |
| 8 | or/5-7 | 83761 |
| 9 | exp child/ or exp "congenital, hereditary, and neonatal diseases and abnormalities"/ or exp infant/ or adolescent/ or exp pediatrics/ or child, abandoned/ or exp child, exceptional/ or child, orphaned/ or child, unwanted/ or minors/ or (pediatric* or paediatric* or child* or newborn* or congenital* or infan* or baby or babies or neonat* or pre-term or preterm* or premature birth* or NICU or preschool* or pre-school* or kindergarten* or kindergarden* or elementary school* or nursery school* or (day care* not adult*) or schoolchild* or toddler* or boy or boys or girl* or middle school* or pubescen* or juvenile* or teen* or youth* or high school* or adolesc* or pre-pubesc* or prepubesc*).mp. or (child* or adolesc* or pediat* or paediat*).jn. | 6089944 |
| 10 | epidemiology/ | 233095 |
| 11 | exp mortality/ | 1181564 |
| 12 | disease severity/ | 584868 |
| 13 | exp morbidity/ | 385387 |
| 14 | prognosis/ | 640887 |
| 15 | exp treatment outcome/ | 1731676 |
| 16 | intensive care unit/ or neonatal intensive care unit/ or pediatric intensive care unit/ | 182910 |
| 17 | (epidemiolog* or death? or fatalit* or mortalit* or outcome? or sever*).tw,kw. | 7968652 |
| 18 | (clinical or incidence? or morbidit* or prevalence? or prognos*).tw,kw. | 7795080 |
| 19 | (intensive care or ICU or ICUs or NICU or NICUs).tw,kw. | 295991 |
| 20 | or/10-19 | 13449668 |
| 21 | 8 and 9 and 20 | 5678 |
| 22 | ("20200810" or "20200811" or "20200812" or "20200813" or "20200814" or "20200815" or "20200816" or "20200817" or "20200818" or "20200819" or "20200820" or "20200821" or "20200822" or "20200823" or "20200824" or "20200825" or "20200826" or "20200827" or "20200828" or "20200829" or "20200830" or "20200831").dc. | 115946 |
| 23 | (202009* or 202010* or 202011* or 202012*).dc. | 767347 |
| 24 | or/22-23 | 883293 |
| 25 | 21 and 24 | 3451 |

**Reviewers for non-English data**

Full text of non-English articles were reviewed by HJ and HA for Arabic, RL, RS, IK, and ZJ for Chinese, SI, MI and PP for French, TK, MK, HH, and NM for Japanese, LC and IJ for Korean, BC and LB for Portuguese, MK for Russian, HG for Farsi, and MG and AB for Spanish. MK, HG, and EH translated extracted Russian, Farsi, and Turkish data to English, and TK and CK reviewed the translated data as a second reviewer.

**Overlapping data check**

For all included articles, patients’ demographic, clinical characteristics, outcome, geographical data of the report, name of health care facility, and duration of the study period were extracted. Based on these extracted data, we performed an elaborate analysis evaluating any possibility of overlapping. If we cannot recognize that cases described in two or more articles are fully distinct due to the ambiguity of information, we considered them as “a possibility of overlapping”. If there is a possibility that any cases from two or more articles are the same patients (overlap), then the article with the most comprehensive data for each outcome is only included. A few simple examples are provided below in case of multiple articles or reports identified in one country.

Example 1

Article A: a case series with 100 infant (<1y) cases from 20 hospitals (including hospital C) in a province with both fatality and ICU outcomes.

Article B: a case report with an infant (8 months old) from hospital C in the same province with both fatality and ICU outcomes.

Decision: Articles A is included. Article B is excluded as there is a possibility that this 8 month old is included in Article A.

\

Example 2 (This overlap check in national reports and articles were performed during article screening process in search 3. For search 1 and 2, this was performed after extracting all articles)

National report D: a nationwide report with 10,000 cases reporting only fatality outcome and no ICU outcome reported.

Article E: a nationwide large cohort study with 8,000 case report with both fatality and ICU outcomes.

Decision: national report D is included for fatality outcome is included. Article E is not included for fatality outcome analysis, but included for ICU outcome analysis.

Example 3

Article F: a large cohort study reporting 10,000 cases from 100 anonymous hospitals in a country

with both fatality and ICU outcomes, but no details for neonatal data were provided.

Article G: a case series from hospital H in the same country reporting 100 cases with both fatality and ICU outcomes.

Article I: a nationwide multicenter study from 10 neonatal units in the country reporting 50 neonatal cases with both fatality and ICU outcomes

Decision: Articles F is included. Article G is excluded. Article I is excluded for fatality and ICU outcome analysis, but included in neonatal outcome analysis.

Example 4

Article J: a cohort study about a school outbreak in province K reporting 300 cases with only fatality outcome (no health care facility data)

Article L: a case series from a clinic in province K reporting 50 cases with only fatality outcome.

Decision: Article J is included. Article L is excluded as there is a possibility that some cases in article L is overlapped with cases in article J, and article J is more comprehensive regarding the outcome of interest.

Example 5

Article M: a case series reporting 5 cases from hospital N, and all of them are teenagers

Article O: a case series reporting 5 cases from hospital N, and all of them are infants

Decision: Both article M and O are included as patients’ populations are distinct with no chance of overlapping cases between two studies.

**Data analysis**

Adult case fatality calculation

In our protocol registered with PROSPERO (registration number: CRD42020179696), we described that the proportion of pediatric case fatality rate/total case fatality rate in each country would also be calculated to effectively compare the difference of the magnitude of effect on children and adult. However, because the significant difference in pediatric CFR between HIC and LMIC was found to be significant in our study, we did not calculate the specific adult CFR.
