# Supplementary figures and images for: The differential impact of pediatric COVID-19 between high-income countries and low- and middle-income countries: A systematic review of fatality and ICU admission in children worldwide
Source: PLoS One. 2021 Jan 29;16(1):e0246326. doi: 10.1371/journal.pone.0246326 (PMC7845974; doi:10.1371/journal.pone.0246326)

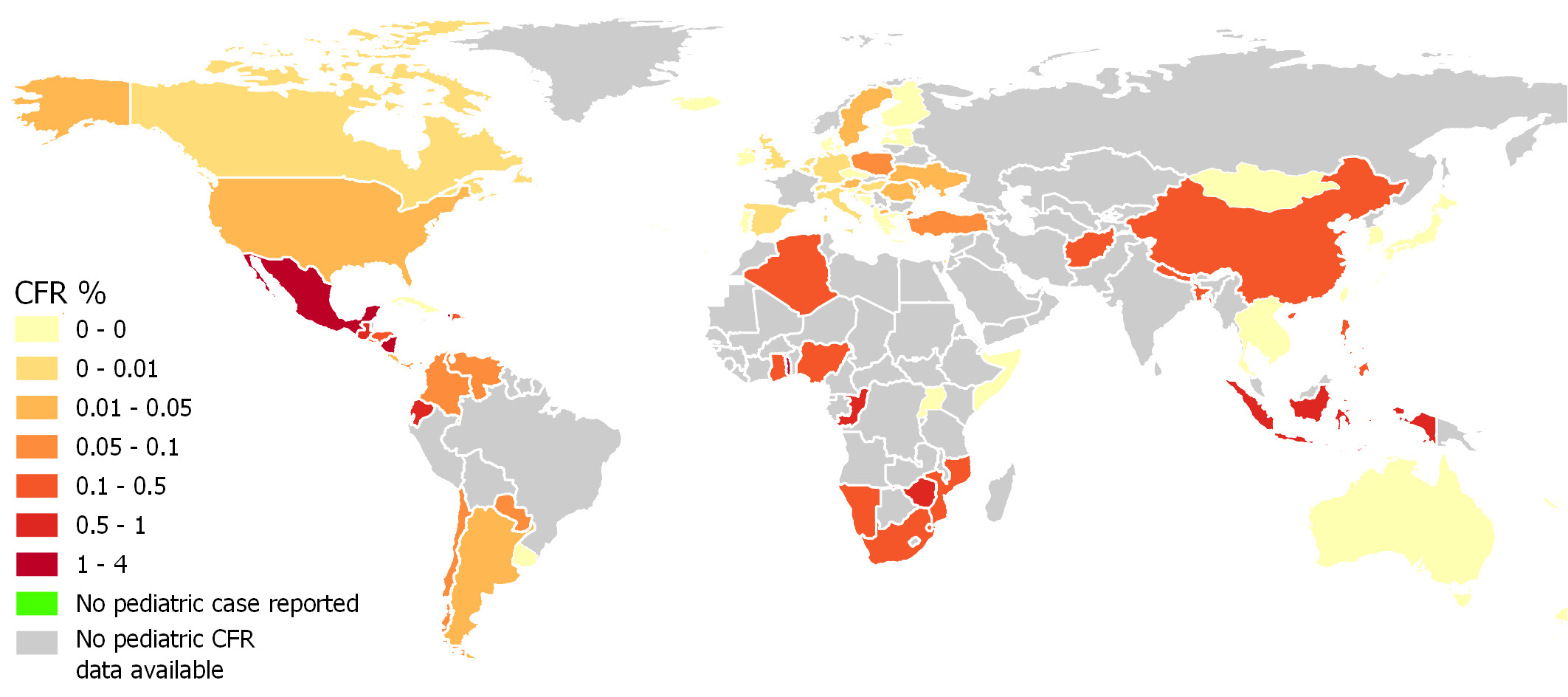

Supplement: S1 Fig — Abbreviations: CFR, case fatality rate. CFRs are presented in percentages (%). Countries of no pediatric case reported includes the country clearly report that there was no confirmed case in children in the national report as of December 7, 2020. National reports published more than 2 months before December 7 are not included, if the countries are CDC COVID-19 Level 2–4 since the date of report. (TIF) [file pone.0246326.s003.tif]

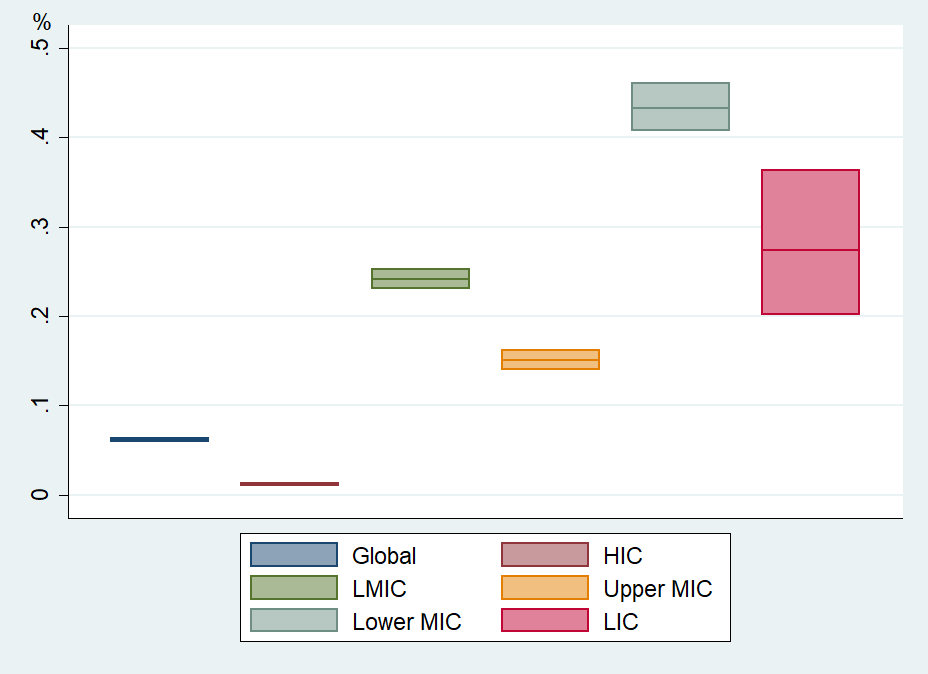

Supplement: S2 Fig — The ranges are presented by 95% confidence intervals of each proportion. Global includes all countries defined by World income. Abbreviations: HICs, high-income countries; LMICs, low- and middle-income countries; MICs, middle-income countries; LICs, low-income countries. (TIF) [file pone.0246326.s004.tif]

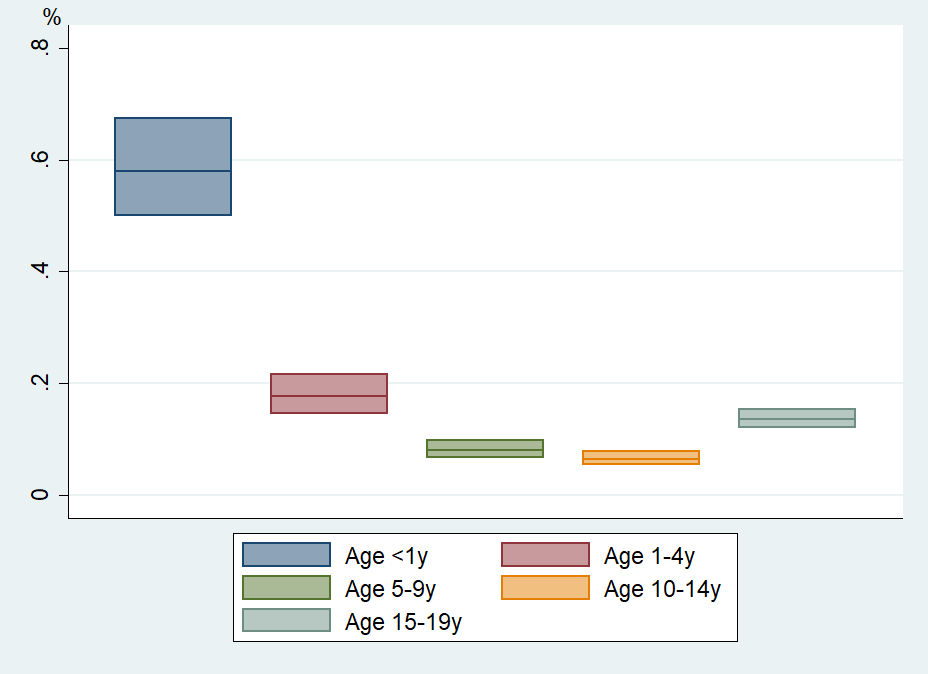

Supplement: S3 Fig — A. Age-specific case fatality rate. B. Age-specific ICU admission rate. Age-specific national data with up to one year difference of age buckets were included. For example, age-specific national data reporting outcomes of 1–5 years and 10–15 years were included in our calculation of 1–4 years and 10–14 years. Abbreviations: y, years. (ZIP) [file pone.0246326.s005.zip › S3A_Fig.tif]

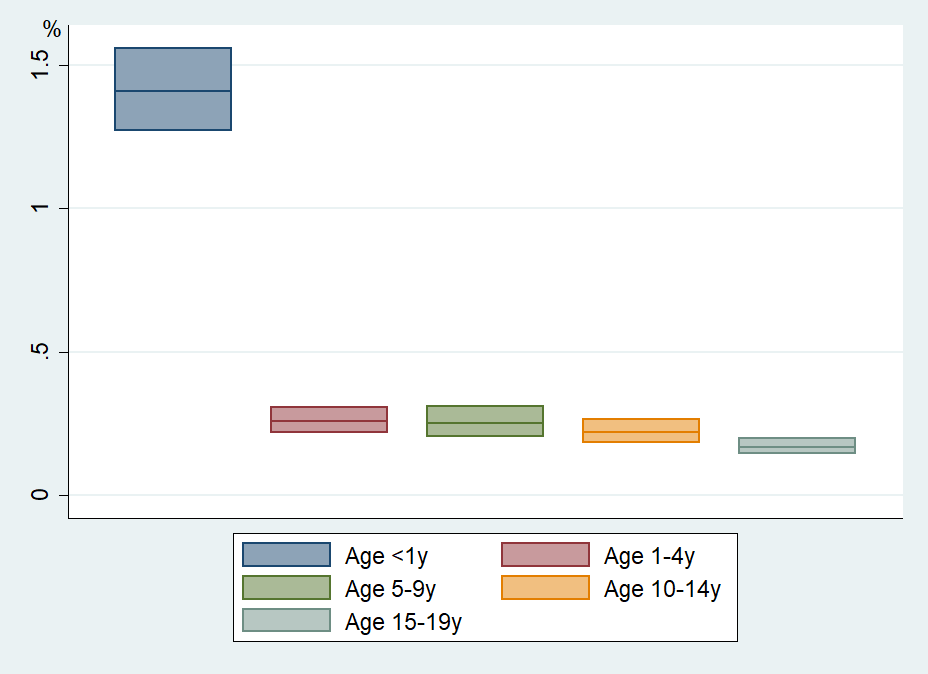

Supplement: S3 Fig — A. Age-specific case fatality rate. B. Age-specific ICU admission rate. Age-specific national data with up to one year difference of age buckets were included. For example, age-specific national data reporting outcomes of 1–5 years and 10–15 years were included in our calculation of 1–4 years and 10–14 years. Abbreviations: y, years. (ZIP) [file pone.0246326.s005.zip › S3B_Fig.tif]
