# Supplementary material for: The differential impact of pediatric COVID-19 between high-income countries and low- and middle-income countries: A systematic review of fatality and ICU admission in children worldwide
Source: PLoS One. 2021 Jan 29;16(1):e0246326. doi: 10.1371/journal.pone.0246326 (PMC7845974; doi:10.1371/journal.pone.0246326)
Supplement: S1 Table — (DOCX) [file pone.0246326.s006.docx]

**S1 Table. Included articles from database search**

| **Authors** | | **Country** | **0-19 years Number of cases** | | **0-19 years Number of deaths** | | **0-19 years Number of ICU**  **admissions** | | **<1 month Number of cases** | | **<1 year Number of cases** | **1-4 years Number of cases** | **5-9 years Number of cases** | | **10-14 years Number of cases** | | **15-19 years Number of cases** | |  |
| --- | --- | --- | --- | --- | --- | --- | --- | --- | --- | --- | --- | --- | --- | --- | --- | --- | --- | --- | --- |
| Khesrani 2020 ^1^ | | Algeria | 1 | | 1 | | N/A | |  | |  |  | 1 | |  | |  | |  |
| Cairoli 2020 ^2^ | | Argentina | 191 | | 0 | | 0 | |  | |  |  |  | |  | |  | |  |
| Ibrahim 2020 ^2^ | | Australia |  | |  | |  | |  | |  |  |  | |  | |  | |  |
| Macartney 2020 ^4^ | | Australia | 97 | | 0 | | 1 | |  | |  |  |  | |  | |  | |  |
| Farmer 2020 ^5^ | | Australia | 621 | | N/A | | 0 | |  | |  |  |  | |  | |  | |  |
| Presler 2020 ^6^ | | Austria | 2 | | 0 | | 1 | | 2 | | 2 |  |  | |  | |  | |  |
| Saha 2020 ^7^ | | Bangladesh | 26 | | 8 | | N/A | | 26 | | 26 |  |  | |  | |  | |  |
| Piersigilli 2020 ^8^ | | Belgium | 1 | | 0 | | 1 | | 1 | | 1 |  |  | |  | |  | |  |
| Garau 2020 ^9^ | | Belgium | 1 | | 0 | | 1 | |  | |  |  |  | |  | | 1 | |  |
| Escalera-Antezana 2020 ^10^ | | Bolivia | 9 | | 0 | | N/A | |  | |  |  |  | |  | |  | |  |
| Escalera-Antezana 2020 ^11^ | | Bolivia | 2 | | 0 | | 0 | |  | |  |  |  | | 1 | | 1 | |  |
| Arapovic 2020 ^12^ | | Bosnia and Herzegovina | 36 | | 0 | | 0 | |  | |  |  |  | |  | |  | |  |
| Aragao 2020 ^13^ | | Brazil | 1 | | 0 | | 0 | |  | |  | 1 |  | |  | |  | |  |
| Frank 2020 ^14^ | | Brazil | 1 | | 0 | | 0 | |  | |  |  |  | |  | | 1 | |  |
| Schaan 2020 ^15^ | | Brazil | 2 | | 0 | | 2 | | 0 | | 1 |  | 1 | |  | |  | |  |
| Florencio 2020 ^16^ | | Brazil | 1 | | 0 | | 0 | |  | |  |  |  | | 1 | |  | |  |
| deMirandaHenriques-Souza 2020 ^17^ | | Brazil | 1 | | 0 | | 1 | |  | |  |  |  | | 1 | |  | |  |
| Bezerra 2020 ^18^ | | Brazil | 1 | | 0 | | 1 | |  | |  | 1 |  | |  | |  | |  |
| Carvalho 2020 ^19^ | | Brazil | 1 | | 0 | | 0 | | 1 | | 1 |  |  | |  | |  | |  |
| Almeida 2020 ^20^ | | Brazil | 1 | | 0 | | 0 | |  | |  |  |  | |  | |  | |  |
| Giorno 2020 ^21^ | | Brazil | 34 | | 3 | | 10 | |  | |  |  |  | |  | |  | |  |
| Rabha 2020 ^22^ | | Brazil | 115 | | 0 | | 12 | |  | |  |  |  | |  | |  | |  |
| Silva 2020 ^23^ | | Brazil | 2 | | 0 | | 0 | |  | |  |  | 1 | | 1 | |  | |  |
| Prata-Barbosa 2020 ^24^ | | Brazil | 69 | | 2 | | 69 | |  | | 17 | 22 |  | |  | |  | |  |
| Hillesheim 2020 ^25^ | | Brazil | 6989 | | 661 | | N/A | |  | | 1341 | 1918 | 1073 | | 954 | | 1703 | |  |
| Magalhaes 2020 ^26^ | | Brazil | 12 | | 1 | | 0 | |  | |  |  |  | |  | |  | |  |
| deSiqueira Alves Lopes 2020 ^27^ | | Brazil | 6038 | | 37 | | 14 | | N/A | | 989 | 935 |  | |  | | 2815 | |  |
| Wong 2020 ^28^ | | Brunei | 2 | | 0 | | 0 | |  | |  |  | 1 | | 1 | |  | |  |
| George 2020 ^28^ | | Brunei | 12 | | 0 | | 0 | | 0 | | 2 | 3 | 2 | | 5 | | 0 | |  |
| Wong 2020 ^30^ | | Brunei | 23 | | 0 | | N/A | |  | |  |  |  | |  | |  | |  |
| Ladha 2020 ^31^ | | Canada | 1 | | 0 | | 0 | |  | |  |  |  | |  | | 1 | |  |
| Kirtsman 2020 ^32^ | | Canada | 1 | | 0 | | 0 | | 1 | | 1 |  |  | |  | |  | |  |
| Diaz-Corvillon 2020 ^33^ | | Chile | 3 | | 0 | | 0 | | 2 | | 2 |  |  | |  | | 1 | |  |
| Geng 2020 ^34^ | | China | 1 | | 0 | | 0 | |  | |  |  |  | |  | | 1 | |  |
| Xu 2020 ^35^ | | China | 5 | | 0 | | 0 | |  | |  |  |  | |  | |  | |  |
| Zhang 2020 ^36^ | | China | 1 | | 0 | | 0 | |  | |  |  |  | |  | | 1 | |  |
| Chang 2020 ^37^ | | China | 2 | | 0 | | 0 | | 0 | | 0 | 1 | 0 | | 0 | | 1 | |  |
| Sun 2020 ^38^ | | China | 4 | | 0 | | 0 | |  | |  |  |  | |  | |  | |  |
| Zhao 2020 ^39^ | | China | 14 | | 0 | | 0 | |  | |  |  |  | |  | |  | |  |
| Gao 2020 ^40^ | | China | 11 | | 0 | | 0 | |  | |  |  |  | |  | |  | |  |
| Liu 2020 ^41^ | | China | 1 | | 0 | | 0 | | 0 | | 1 | 0 | 0 | | 0 | | 0 | |  |
| Liu 2020 ^42^ | | China | 1 | | 0 | | 0 | |  | |  |  |  | |  | |  | |  |
| Jiang 2020 ^43^ | | China | 1 | | 0 | | 0 | |  | |  |  | 1 | |  | |  | |  |
| Lu 2020 ^44^ | | China | 1 | | 0 | | 0 | |  | |  |  | 1 | |  | |  | |  |
| Bai 2020 ^45^ | | China | 25 | | 0 | | 0 | |  | |  |  |  | |  | |  | |  |
| Wei 2020 ^46^ | | China | 1 | | 0 | | 0 | | 1 | | 1 | 0 | 0 | | 0 | | 0 | |  |
| Zhang 2020 ^47^ | | China | 3 | | 0 | | 0 | | 0 | | 1 |  |  | | 2 | |  | |  |
| Liu 2020 ^48^ | | China | 1 | | 0 | | 0 | | 0 | | 1 |  |  | |  | |  | |  |
| Ren 2020 ^49^ | | China | 1 | | 0 | | 0 | |  | |  |  |  | |  | | 1 | |  |
| Xu 2020 ^50^ | | China | 10 | | 0 | | 0 | |  | |  |  |  | |  | |  | |  |
| Song 2020 ^51^ | | China | 1 | | 0 | | 0 | | 0 | | 0 | 1 | 0 | | 0 | | 0 | |  |
| Yang 2020 ^52^ | | China | 11 | | 0 | | 0 | |  | |  |  |  | |  | |  | |  |
| Zhong 2020 ^53^ | | China | 2 | | 0 | | 0 | |  | |  |  |  | |  | |  | |  |
| Zhao 2020 ^54^ | | China | 1 | | 0 | | 0 | |  | |  |  | 1 | |  | |  | |  |
| Yang 2020 ^55^ | | China | 10 | | 0 | | 0 | | 0 | | 2 | 3 | 4 | | 1 | | 0 | |  |
| Gao 2020 ^56^ | | China | 7 | | 0 | | 0 | |  | |  |  |  | |  | |  | |  |
| Zhao 2020 ^57^ | | China | 7 | | 0 | | 0 | |  | |  |  |  | |  | |  | |  |
| Li 2020 ^58^ | | China | 1 | | 0 | | 0 | |  | |  | 1 |  | |  | |  | |  |
| Jiao 2020 ^59^ | | China | 1 | | 0 | | 0 | | 0 | | 1 |  |  | |  | |  | |  |
| Chen 2020 ^60^ | | China | 1 | | 0 | | 0 | |  | |  |  |  | |  | | 1 | |  |
| Sun 2020 ^61^ | | China | 4 | | 0 | | 0 | |  | |  |  |  | |  | |  | |  |
| Chen 2020 ^62^ | | China | 1 | | 0 | | 0 | |  | |  |  |  | | 1 | |  | |  |
| Qian 2020 ^63^ | | China | 1 | | 0 | | 0 | | 0 | | 0 | 1 | 0 | | 0 | | 0 | |  |
| Xing 2020 ^64^ | | China | 3 | | 0 | | 0 | |  | |  | 1 | 2 | |  | |  | |  |
| Jia 2020 ^65^ | | China | 19 | | 0 | | 0 | |  | |  |  |  | |  | |  | |  |
| Wang 2020 ^66^ | | China | 35 | | 0 | | 0 | |  | |  |  |  | |  | |  | |  |
| Wang 2020 ^67^ | | China | 1 | | 0 | | 0 | |  | |  |  |  | |  | | 1 | |  |
| Liu 2020 ^68^ | | China | 6 | | 0 | | 0 | |  | |  |  |  | |  | |  | |  |
| ji 2020 ^69^ | | China | 4 | | 0 | | 0 | | 0 | | 2 | 1 | 0 | | 1 | | 0 | |  |
| Feng 2020 ^70^ | | China | 1 | | 0 | | 0 | |  | |  |  |  | |  | | 1 | |  |
| Li 2020 ^71^ | | China | 1 | | 0 | | 0 | | 0 | | 1 |  |  | |  | |  | |  |
| Zhang 2020 ^72^ | | China | 3 | | 0 | | 0 | |  | |  |  | 3 | |  | |  | |  |
| Lei 2020 ^73^ | | China | 1 | | 0 | | 0 | |  | |  |  | 1 | |  | |  | |  |
| Wu 2020 ^74^ | | China | 2 | | 0 | | 2 | | 2 | | 2 |  |  | |  | |  | |  |
| Wu 2020 ^75^ | | China | 157 | | 2 | | N/A | |  | |  |  |  | |  | |  | |  |
| Liu 2020 ^76^ | | China | 46 | | 1 | | N/A | | 7 | | 46 |  |  | |  | |  | |  |
| Lan 2020 ^77^ | | China | 4 | | 0 | | 0 | |  | |  |  | 2 | | 2 | |  | |  |
| Sun 2020 ^78^ | | China | 8 | | 0 | | 8 | | 0 | | 2 | 2 | 1 | | 2 | | 1 | |  |
| Lu 2020 ^79^ | | China | 171 | | 1 | | 3 | |  | | 31 | 40 | 58 | | 42 | | 0 | |  |
| Zeng 2020 ^80^ | | China | 3 | | 0 | | 3 | | 3 | | 3 | 0 | 0 | | 0 | | 0 | |  |
| Jiang 2020 ^81^ | | China | 2 | | 0 | | 1 | | 0 | | 0 | 1 | 1 | | 0 | | 0 | |  |
| Liu 2020 ^82^ | | China | 6 | | 0 | | 1 | | 0 | | 0 | 5 | 1 | | 0 | | 0 | |  |
| Wang 2020 ^83^ | | China | 3 | | 0 | | 0 | |  | |  |  |  | |  | | 3 | |  |
| Wang 2020 ^84^ | | China | 260 | | 1 | | - | |  | |  |  |  | |  | |  | |  |
| Gao 2020 ^85^ | | China | 1 | | 0 | | 0 | | 0 | | 0 | 0 | 1 | | 0 | | 0 | |  |
| Zhang 2020 ^86^ | | China | 2 | | 0 | | 0 | |  | |  | 1 |  | |  | |  | |  |
| Li 2020 ^87^ | | China | 8 | | 0 | | 1 | |  | |  | 7 | 1 | |  | |  | |  |
| Chen 2020 ^88^ | | China | 2 | | 0 | | 0 | |  | |  |  | 2 | |  | |  | |  |
| Zheng 2020 ^89^ | | China | 1 | | 0 | | 0 | | 0 | | 0 | 0 | 1 | | 0 | | 0 | |  |
| Li 2020 ^89^ | | China | 1 | | 0 | | 1 | | 1 | | 1 |  |  | |  | |  | |  |
| Li 2020 ^91^ | | China | 1 | | 0 | | 0 | | 0 | | 0 | 0 | 0 | | 0 | | 1 | |  |
| Wu 2020 ^92^ | | China | 35 | | 0 | | 2 | |  | |  |  |  | |  | |  | |  |
| Li 202 ^93^ | | China | 1 | | 0 | | 0 | | 0 | | 1 |  |  | |  | |  | |  |
| Wu 2020 ^94^ | | China | 1 | | 0 | | 0 | | 0 | | 1 | 0 | 0 | | 0 | | 0 | |  |
| Li 2020 ^95^ | | China | 1 | | 0 | | 0 | |  | |  | 1 |  | |  | |  | |  |
| Wei 2020 ^96^ | | China | 4 | | 0 | | 0 | |  | |  |  |  | |  | |  | |  |
| Sun 2020 ^97^ | | China | 1 | | 0 | | 0 | | 1 | | 1 |  |  | |  | |  | |  |
| Li 2020 ^98^ | | China | 2 | | 0 | | 0 | |  | |  | 2 |  | |  | |  | |  |
| Yu 2020 ^99^ | | China | 1 | | 0 | | 0 | | 1 | | 1 |  |  | |  | |  | |  |
| Tang 2020 ^100^ | | China | 1 | | 0 | | 0 | |  | |  |  |  | | 1 | |  | |  |
| Li 2020 ^101^ | | China | 6 | | 0 | | 0 | |  | |  |  |  | |  | |  | |  |
| Zhu 2020 ^102^ | | China | 1 | | 0 | | 0 | |  | |  |  |  | | 1 | |  | |  |
| Zheng 2020 ^103^ | | China | 52 | | 0 | | 0 | |  | |  |  |  | |  | |  | |  |
| Fu 2020 ^104^ | | China | 1 | | 0 | | 0 | |  | |  |  | 1 | |  | |  | |  |
| Yuxia Cui 2020 ^105^ | | China | 1 | | 0 | | 0 | | 0 | | 1 |  |  | |  | |  | |  |
| Zhu 2020 ^106^ | | China | 10 | | 0 | | 0 | |  | |  | 2 | 3 | | 4 | | 1 | |  |
| Wu 2020 ^107^ | | China | 23 | | 0 | | 0 | |  | |  |  |  | |  | |  | |  |
| Wang 2020 ^108^ | | China | 31 | | 0 | | 0 | |  | |  |  |  | |  | |  | |  |
| Lin 2020 ^109^ | | China | 56 | | 0 | | 0 | |  | |  |  |  | |  | |  | |  |
| Song 2020 ^110^ | | China | 8 | | 0 | | 0 | | 0 | | 2 | 2 | 3 | | 1 | | 0 | |  |
| Tan 2020 ^111^ | | China | 10 | | 0 | | 0 | | 0 | | 0 | 3 | 5 | | 2 | | 0 | |  |
| Xu 2020 ^112^ | | China | 10 | | 0 | | 0 | | 0 | | 1 | 4 | 1 | | 3 | | 1 | |  |
| Lei 2020 ^113^ | | China | 1 | | 0 | | 0 | |  | |  |  |  | | 1 | |  | |  |
| Huang 2020 ^114^ | | China | 1 | | 0 | | 0 | | 0 | | 0 | 0 | 0 | | 0 | | 1 | |  |
| Su 2020 ^115^ | | China | 9 | | 0 | | 0 | | 0 | | 2 | 3 | 4 | |  | |  | |  |
| Du 2020 ^116^ | | China | 14 | | 0 | | 0 | |  | |  |  |  | |  | |  | |  |
| Fan 2020 ^117^ | | China | 1 | | 0 | | 0 | | 0 | | 1 | 0 | 0 | | 0 | | 0 | |  |
| Wang 2020 ^118^ | | China | 3 | | 0 | | 0 | |  | |  |  |  | | 1 | | 2 | |  |
| Chan 2020 ^119^ | | China | 1 | | 0 | | 0 | | 0 | | 0 | 0 | 0 | | 1 | | 0 | |  |
| Guiqing 2020 ^120^ | | China | 1 | | 0 | | 0 | |  | |  |  |  | | 1 | |  | |  |
| Zheng 2020 ^121^ | | China | 25 | | 0 | | 2 | | 0 | | 10 | 6 | 9 | | 0 | | 0 | |  |
| Chen 2020 ^122^ | | China | 1 | | 0 | | 0 | |  | |  |  |  | | 1 | |  | |  |
| Liu 2020 ^123^ | | China | 2 | | 0 | | 0 | |  | |  |  | 1 | |  | | 1 | |  |
| Han 2020 ^124^ | | China | 7 | | 0 | | 0 | |  | |  |  |  | |  | |  | |  |
| Song 2020 ^125^ | | China | 16 | | 0 | | 0 | | 0 | | 1 | 3 | 5 | | 7 | | 0 | |  |
| Lou 2020 ^126^ | | China | 3 | | 0 | | 0 | | 0 | | 1 | 0 | 2 | | 0 | | 0 | |  |
| Li 2020 ^127^ | | China | 1 | | 0 | | 0 | |  | |  |  | 1 | |  | |  | |  |
| Li 2020 ^128^ | | China | 5 | | 0 | | 0 | | 0 | | 1 | 3 | 1 | | 0 | | 0 | |  |
| Lin 2020 ^129^ | | China | 1 | | 0 | | 0 | | 0 | | 0 | 0 | 1 | | 0 | | 0 | |  |
| Xu 2020 ^130^ | | China | 2 | | 0 | | 0 | | 0 | | 0 | 0 | 0 | | 1 | | 1 | |  |
| Lo 2020 ^131^ | | China | 1 | | 0 | | 0 | |  | |  |  |  | |  | | 1 | |  |
| Dong 2020 ^312^ | | China | 2143 | | 1 | | N/A | | N/A | | 86 | 137 | 171 | | 180 | | 157 | |  |
| Lendorf 2020 ^133^ | | Denmark | 5 | | 0 | | 0 | |  | | 4 |  |  | |  | | 1 | |  |
| Ali 2020 ^134^ | | Egypt | 1 | | 0 | | 0 | |  | |  |  | 1 | |  | |  | |  |
| Nofal 2020 ^135^ | | Egypt | 2 | | 0 | | 0 | |  | |  |  | 2 | |  | |  | |  |
| Ministerio de Salud 2020 ^136^ | | El Salvador | 7 | | 1 | | N/A | |  | |  |  |  | |  | |  | |  |
| Gotzinger 2020 ^137^ | | Europe | 582 | | 4 | | 48 | | 40 | |  |  |  | |  | |  | |  |
| Martenot 2020 ^138^ | | France | 1 | | 0 | | 0 | | 1 | |  |  |  | |  | |  | |  |
| Tchidjou 2020 ^139^ | | France | 1 | | 0 | | 0 | | 1 | | 1 |  |  | |  | |  | |  |
| Tchidjou 2020 ^140^ | | France | 1 | | 0 | | 0 | | 0 | | 1 |  |  | |  | |  | |  |
| Danis 2020 ^141^ | | France | 1 | | 0 | | 0 | | 0 | | 0 | 0 | 1 | | 0 | | 0 | |  |
| LeRoux 2020 ^142^ | | France | 1 | | 0 | | 0 | | 0 | | 1 |  |  | |  | |  | |  |
| Morand 2020 ^143^ | | France | 1 | | 0 | | 0 | |  | |  | 1 |  | |  | |  | |  |
| Aherfi 2020 ^144^ | | France | 9 | | 0 | | N/A | |  | |  |  |  | |  | |  | |  |
| Colson 2020 ^145^ | | France | 20 | | 0 | | N/A | |  | |  | 3 | 7 | | 7 | | 3 | |  |
| Abasse 2020 ^146^ | | France | 1 | | 0 | | 1 | | 1 | | 1 |  |  | |  | |  | |  |
| Heilbronner 2020 ^147^ | | France | 4 | | 0 | | 4 | |  | |  |  |  | | 2 | | 2 | |  |
| Vivanti 2020 ^148^ | | France | 1 | | 0 | | 1 | | 1 | | 1 |  |  | |  | |  | |  |
| Nathan 2020 ^149^ | | France | 5 | | 0 | | 0 | | 0 | | 5 | 0 | 0 | | 0 | | 0 | |  |
| Fischer 2020 ^150^ | | France | 1 | | 0 | | 1 | |  | |  |  |  | |  | | 1 | |  |
| Nathan 2020 ^151^ | | France | 23 | | 0 | | 5 | |  | |  |  |  | |  | |  | |  |
| Oualha 2020 ^152^ | | France | 27 | | 5 | | 27 | |  | |  |  |  | |  | |  | |  |
| Gaborieau 2020 ^153^ | | France | 157 | | 3 | | 16 | | 32 | | 90 |  |  | |  | |  | |  |
| Meslin 2020 ^154^ | | France | 6 | | 0 | | 0 | | 4 | | 6 |  |  | |  | |  | |  |
| Loron 2020 ^155^ | | France | 1 | | 0 | | 1 | | 0 | | 1 |  |  | |  | |  | |  |
| Nazon 2020 ^156^ | | France | 1 | | 0 | | 0 | |  | |  |  |  | |  | | 1 | |  |
| Lorenz 2020 ^157^ | | Germany | 1 | | 0 | | 1 | | 1 | | 1 |  |  | |  | |  | |  |
| Olfe 2020 ^158^ | | Germany | 1 | | 0 | | 0 | |  | |  |  |  | |  | | 1 | |  |
| Wehl 2020 ^159^ | | Germany | 1 | | 0 | | 0 | | 0 | | 1 |  |  | |  | |  | |  |
| Kim-Hellmuth 2020 ^160^ | | Germany | 1 | | 0 | | 1 | | ^298^ | | ^299^ | 1 |  | |  | |  | |  |
| Farber 2020 ^161^ | | Germany | 1 | | 0 | | 0 | | 0 | | 1 |  |  | |  | |  | |  |
| Koczulla 2020 ^162^ | | Germany | 1 | | 0 | | 0 | |  | |  |  |  | |  | | 1 | |  |
| Wolf 2020 ^163^ | | Germany | 2 | | 0 | | 0 | |  | |  | 1 | 1 | |  | |  | |  |
| Armann 2020 ^164^ | | Germany | 128 | | 1 | | 16 | |  | |  |  |  | |  | |  | |  |
| Ayisi-Boateng 2020 ^165^ | | Ghana | 1 | | 0 | | 0 | |  | |  |  |  | | 1 | |  | |  |
| Maltezou 2020 ^166^ | | Greece | 203 | | 0 | | 1 | |  | | 23 |  |  | |  | |  | |  |
| Maltezou 2020 ^167^ | | Greece | 13 | | 0 | | 0 | |  | |  | 2 | 3 | | 5 | | 3 | |  |
| Majachani 2020 ^168^ | | Grenada | 1 | | 0 | | 1 | | 1 | | 1 |  |  | |  | |  | |  |
| Mak 2020 ^169^ | | Hong Kong | 3 | | 0 | | 0 | |  | |  |  |  | | 1 | | 2 | |  |
| Rost 2020 ^170^ | | Hungary | 44 | | 0 | | N/A | |  | |  |  |  | |  | |  | |  |
| Dosi 2020 ^171^ | | India | 36 | | 0 | | N/A | |  | |  |  |  | |  | |  | |  |
| Singh 2020 ^172^ | | India | 1 | | 0 | | 0 | | 1 | | 1 |  |  | |  | |  | |  |
| Kant 2020 ^173^ | | India | 3 | | 0 | | N/A | |  | |  |  |  | |  | | 3 | |  |
| Meghwal 2020 ^174^ | | India | 2 | | 0 | | 0 | |  | |  |  |  | |  | |  | |  |
| Mohakud 2020 ^175^ | | India | 1 | | 0 | | 1 | | 1 | | 1 |  |  | |  | |  | |  |
| Radhakrishnan 2020 ^176^ | | India | 15 | | 0 | | 1 | |  | | 0 | 6 | 1 | | 3 | | 5 | |  |
| Daniel 2020 ^177^ | | India | 1 | | 0 | | 1 | |  | |  |  |  | |  | | 1 | |  |
| Nayak 2020 ^178^ | | India | 3 | | 0 | | N/A | | 3 | | 3 | N/A |  | |  | |  | |  |
| Kalamdani 2020 ^179^ | | India | 12 | | 0 | | 0 | | 12 | | 12 |  |  | |  | |  | |  |
| Gupta 2020 ^180^ | | India | 1 | | 0 | | 0 | | 0 | | 0 | 0 | 0 | | 0 | | 1 | |  |
| Bandyopadhyay 2020 ^181^ | | India | 1 | | 0 | | 1 | | 1 | | 1 |  |  | |  | |  | |  |
| Anand 2020 ^182^ | | India | 7 | | 0 | | 1 | | 7 | | 7 |  |  | |  | |  | |  |
| Shankar 2020 ^183^ | | India | 1 | | 0 | | 0 | |  | |  | 1 |  | |  | |  | |  |
| Kulkarni 2020 ^184^ | | India | 1 | | 0 | | 0 | | 1 | | 1 |  |  | |  | |  | |  |
| Kulkarni 2020 ^3185^ | | India | 1 | | 1 | | 1 | |  | |  | 1 |  | |  | |  | |  |
| Sarangi 2020 ^186^ | | India | 50 | | 0 | | 0 | |  | |  |  |  | |  | |  | |  |
| Saraswathi 2020 ^187^ | | India | 238 | | 0 | | N/A | |  | |  |  |  | |  | |  | |  |
| Gupta 2020 ^188^ | | India | 85 | | 3 | | 3 | |  | |  |  |  | |  | |  | |  |
| Laxminarayan 2020 ^189^ | | India | 34506 | | 17 | | N/A | |  | |  |  |  | |  | |  | |  |
| Banerjee 2020 ^190^ | | India | 41 | | 1 | | 6 | | 6 | | 18 | 15 | 6 | |  | |  | |  |
| Marhaeni 2020 ^191^ | | Indonesia | 1 | | 0 | | 0 | |  | |  |  |  | |  | | 1 | |  |
| Sumarni 2020 ^192^ | | Indonesia | 1 | | 0 | | 1 | | 0 | | 1 |  |  | |  | |  | |  |
| EsmaeiliDooki 2020 ^193^ | | Iran | 13 | | 0 | | N/A | |  | |  |  |  | |  | |  | |  |
| Mirzaee 2020 ^194^ | | Iran | 1 | | 0 | | N/A | |  | |  |  |  | | 1 | |  | |  |
| Hashemi 2020 ^195^ | | Iran | 3 | | 3 | | 3 | |  | |  | 1 | 2 | |  | |  | |  |
| Noghabi 2020 ^196^ | | Iran | 1 | | 0 | | N/A | | 0 | | 1 |  |  | |  | |  | |  |
| Gharekhanloo 2020 ^197^ | | iran | 2 | | 0 | | 1 | |  | |  |  |  | |  | | 1 | |  |
| Norooznezhad 2020 ^198^ | | Iran | 15 | | 0 | | 0 | |  | |  |  |  | |  | |  | |  |
| Mamishi 2020 ^199^ | | Iran | 25 | | 3 | | N/A | |  | |  |  |  | |  | |  | |  |
| Moradveisi 2020 ^200^ | | Iran | 1 | | 0 | | 1 | |  | |  | 1 |  | |  | |  | |  |
| Navaeifar 2020 ^201^ | | Iran | 1 | | 0 | | 0 | |  | |  | 1 |  | |  | |  | |  |
| Malekhosseini 2020 ^202^ | | Iran | 4 | | 4 | | N/A | |  | |  | 3 | 1 | |  | |  | |  |
| Emami 2020 ^203^ | | Iran | 2 | | 0 | | 2 | | 1 | | 1 | 1 |  | |  | |  | |  |
| Nikoupour 2020 ^204^ | | Iran | 1 | | 1 | | 1 | |  | |  | 1 |  | |  | |  | |  |
| Saeed 2020 ^205^ | | Iran | 1 | | 0 | | 1 | |  | |  | 1 |  | |  | |  | |  |
| Saeed 2020 ^206^ | | Iran | 1 | | 1 | | 1 | |  | |  |  |  | | 1 | |  | |  |
| Ekbatani 2020 ^207^ | | Iran | 3 | | 0 | | 0 | |  | |  | 1 |  | | 2 | |  | |  |
| Nikpouraghdam 2020 ^208^ | | Iran | 10 | | 0 | | N/A | |  | |  |  |  | |  | |  | |  |
| Jafari 2020 ^209^ | | Iran | 1 | | 0 | | 0 | | 0 | | 1 |  |  | |  | |  | |  |
| Eghbali 2020 ^210^ | | Iran | 4 | | 1 | | 2 | |  | |  |  | 1 | | 3 | |  | |  |
| Karimi 2020 ^211^ | | Iran | 1 | | 0 | | 0 | |  | |  |  |  | | 1 | |  | |  |
| Rabizadeh 2020 ^212^ | | Iran | 1 | | 0 | | 1 | |  | |  |  |  | |  | | 1 | |  |
| Rohani 2021 ^213^ | | Iran | 1 | | 0 | | 0 | |  | |  |  | 1 | |  | |  | |  |
| HajiEsmaeilMemar 2020 ^214^ | | Iran | 1 | | 1 | | 1 | |  | |  |  |  | | 1 | |  | |  |
| Dorgalaleh 2020 ^215^ | | Iran | 2 | | 0 | | 1 | | 1 | |  |  |  | |  | | 1 | |  |
| Mahmoudi 2020 ^216^ | | Iran | 1 | | 0 | | 0 | |  | |  |  |  | | 1 | |  | |  |
| Kalantari 2020 ^217^ | | Iran | 18 | | 0 | | N/A | |  | |  |  |  | |  | |  | |  |
| Nasimfar 2020 ^218^ | | Iran | 1 | | 0 | | 0 | |  | |  |  |  | | 1 | |  | |  |
| Soltani 2020 ^219^ | | Iran | 30 | | 1 | | N/A | | 5 | | 6 |  |  | |  | |  | |  |
| Schwartz 2020 ^220^ | | Iran | 18 | | 2 | | 10 | | 18 | | 18 |  |  | |  | |  | |  |
| Hashemi 2020 ^221^ | | Iran | 5 | | 5 | | N/A | |  | |  |  |  | |  | |  | |  |
| Merza 2020 ^222^ | | Iraq | 4 | | 0 | | 0 | | 0 | | 1 | 0 | 1 | | 1 | | 1 | |  |
| Hussein 2020 ^223^ | | Iraq | 125 | | 0 | | N/A | |  | |  |  |  | |  | |  | |  |
| Linnane 2020 ^224^ | | Ireland | 1 | | 0 | | 0 | |  | |  |  |  | | 1 | |  | |  |
| Jacobi 2020 ^225^ | | Israel | 1 | | 0 | | 0 | |  | |  | 1 |  | |  | |  | |  |
| Lopian 2020 ^226^ | | Israel | 1 | | 0 | | 0 | | 1 | |  |  |  | |  | |  | |  |
| Stein-Zamir 2020 ^227^ | | Isreal | 153 | | 0 | | N/A | |  | |  |  |  | | 74 | | 79 | |  |
| Nunziata 2020 ^228^ | | Italy | 102 | | 0 | | 0 | | 1 | | 16 |  |  | |  | |  | |  |
| Vergine 2020 ^229^ | | Italy | 194 | | 0 | | 1 | |  | | 24 | 29 | 37 | |  | |  | |  |
| Parri 2020 ^230^ | | Italy | 170 | | 0 | | 1 | |  | | 61 | 29 | 32 | |  | |  | |  |
| Parri 2020 ^231^ | | Italy | 100 | | 0 | | 9 | | N/A | | 40 | 14 | 17 | | 20 | | 9 | |  |
| Bellino 2020 ^232^ | | Italy | 3836 | | 4 | | 18 | | N/A | | 528 |  |  | |  | |  | |  |
| Garazzino 2020 ^233^ | | Italy | 168 | | 0 | | 2 | | 15 | | 66 | 38 | 24 | |  | |  | |  |
| Irie 2020 ^234^ | | Japan | 2 | | 0 | | 0 | |  | |  |  |  | | 1 | | 1 | |  |
| Kawamura 2020 ^235^ | | Japan | 4 | | 0 | | 0 | | 0 | | 2 | 1 |  | | 1 | |  | |  |
| Kakuya 2020 ^236^ | | Japan | 3 | | 0 | | 0 | |  | |  |  | 2 | | 1 | |  | |  |
| Ishii 2020 ^237^ | | Japan | 13 | | 0 | | 0 | |  | |  |  |  | |  | |  | |  |
| Kasuga 2020 ^238^ | | Japan | 2 | | 0 | | 0 | |  | |  |  |  | | 2 | |  | |  |
| Higuchi 2020 ^238^ | | Japan | 2 | | 0 | | 0 | |  | |  |  |  | |  | |  | |  |
| Sano 2020 ^240^ | | Japan | 1 | | 0 | | 1 | | 0 | | 1 |  |  | |  | |  | |  |
| Alkhatatbeh 2020 ^241^ | | Jordan | 53 | | 0 | | 0 | |  | |  |  |  | |  | |  | |  |
| Kilani 2020 ^242^ | | Jordan | 61 | | 0 | | 0 | | N/A | | 6 |  |  | |  | |  | |  |
| Yusef 2020 ^243^ | | Jordan | 20 | | 0 | | 0 | |  | |  |  |  | |  | |  | |  |
| Semenova 2020 ^244^ | | Kazakhstan | 1000 | | 0 | | N/A | |  | |  |  |  | |  | |  | |  |
| Savic 2020 ^245^ | | Kuwait | 1 | | 0 | | 1 | |  | |  |  |  | | 1 | |  | |  |
| Ayed 2020 ^246^ | | Kuwait | 2 | | 0 | | 2 | | 2 | | 2 |  |  | |  | |  | |  |
| Alsharrah 2020 ^247^ | | Kuwait | 134 | | 0 | | 0 | |  | | 8 |  |  | |  | |  | |  |
| Mansour 2020 ^248^ | | Lebanon | 1 | | 0 | | 0 | |  | |  | 1 |  | |  | |  | |  |
| Oberweis 2020 ^249^ | | Luxembourg | 1 | | 0 | | 1 | |  | |  |  | 1 | |  | |  | |  |
| Vee 2020 ^250^ | | Malaysia | 48 | | 0 | | 0 | |  | |  |  |  | |  | |  | |  |
| See 2020 ^251^ | | Malaysia | 4 | | 0 | | 0 | | 0 | | 0 | 2 | 1 | | 1 | | 0 | |  |
| Castano-Jaramillo 2020 ^252^ | | Mexico | 1 | | 0 | | 0 | | 0 | | 1 |  |  | |  | |  | |  |
| Flores 2020 ^253^ | | Mexico | 3 | | 1 | | 1 | |  | |  | 1 | 2 | |  | |  | |  |
| Hinojosa-Velasco 2020 ^254^ | | Mexico | 1 | | 0 | | 1 | | 1 | | 1 |  |  | |  | |  | |  |
| Olivar-Lopez 2020 ^255^ | | Mexico | 79 | | 1 | | 11 | |  | |  |  |  | |  | |  | |  |
| Lahfaoui 2020 ^256^ | | Morocco | 1 | | 1 | | 1 | | 0 | | 0 | 1 | 0 | | 0 | | 0 | |  |
| Chekhlabi 2020 ^257^ | | Morocco | 15 | | 0 | | N/A | |  | |  |  |  | |  | |  | |  |
| Nassih 2020 ^258^ | | Morocco | 1 | | 0 | | 0 | |  | |  | 1 |  | |  | |  | |  |
| Fakiri 2020 ^259^ | | Morocco | 74 | | 0 | | 0 | |  | |  |  |  | |  | |  | |  |
| Janah 2020 ^260^ | | Morocco | 1 | | 0 | | 0 | |  | |  |  |  | |  | | 1 | |  |
| deSanctis 2020 ^261^ | | Multicenter | 1 | | 0 | | 0 | |  | |  |  |  | | 1 | |  | |  |
| Caro-Dominguez 2020 ^262^ | | Multinational | 91 | | 1 | | 10 | |  | |  |  |  | |  | |  | |  |
| Sola 2020 ^263^ | | Multinational | 6 | | 0 | | N/A | | 6 | | 6 |  |  | |  | |  | |  |
| Slaats 2020 ^264^ | | Netherlands | 1 | | 0 | | 0 | | 1 | |  |  |  | |  | |  | |  |
| Soumana 2020 ^265^ | | Niger | 1 | | 1 | | N/A | | 0 | | 1 |  |  | |  | |  | |  |
| Ibrahim 2020 ^266^ | | Nigeria | 5 | | 0 | | 0 | | 0 | | 1 | 2 | 2 | |  | |  | |  |
| Adedeji 2020 ^267^ | | Nigeria | 53 | | 0 | | 0 | |  | |  | 2 | 8 | | 22 | | 21 | |  |
| Stordal 2020 ^268^ | | Norway | 493 | | 0 | | N/A | |  | | 7 | 50 | 58 | | 125 | | 253 | |  |
| Kristoffersen 2020 ^269^ | | Norway | 1 | | 0 | | 0 | | 0 | | 1 |  |  | |  | |  | |  |
| Alwardi 2020 ^270^ | | Oman | 3 | | 0 | | 3 | | 3 | | 3 |  |  | |  | |  | |  |
| Moazzam 2020 ^271^ | | Pakistan | 1 | | 0 | | 0 | | 0 | | 1 |  |  | |  | |  | |  |
| Alzamora 2020 ^272^ | | Peru | 1 | | 0 | | 1 | | 1 | | 1 | 0 | 0 | | 0 | | 0 | |  |
| Conto-Palomino 2020 ^273^ | | Peru | 1 | | 1 | | 0 | |  | |  |  |  | | 1 | |  | |  |
| Rodriguez-Portilla 2020 ^274^ | | Peru | 1 | | 1 | | 1 | |  | |  |  | 1 | |  | |  | |  |
| Montoya 2020 ^275^ | | Peru | 69 | | 7 | | 3 | |  | |  |  |  | |  | |  | |  |
| Gujski 2020 ^276^ | | Poland | 87 | | 0 | | N/A | |  | |  |  |  | |  | |  | |  |
| Jarmolinski 2020 ^277^ | | Poland | 1 | | 0 | | 0 | |  | |  |  | 1 | |  | |  | |  |
| Correia 2020 ^278^ | | Portugal | 1 | | 0 | | 1 | | 1 | | 1 |  |  | |  | |  | |  |
| PicaodeCarvalho 2020 ^279^ | | Portugal | 103 | | 0 | | 1 | |  | | 22 |  |  | |  | |  | |  |
| Omrani 2020 ^280^ | | Qatar | 131 | | 0 | | 0 | |  | |  |  |  | |  | |  | |  |
| AlKuwari 2020 ^281^ | | Qatar | 302 | | 0 | | N/A | |  | |  |  |  | |  | |  | |  |
| Soliman 2020 ^282^ | | Qatar | 1 | | 0 | | 1 | | 0 | | 1 |  |  | |  | |  | |  |
| Marginean 2020 ^283^ | | Romania | 2 | | 0 | | 1 | | 0 | | 1 |  |  | |  | | 1 | |  |
| Dima 2020 ^284^ | | Romania | 3 | | 0 | | 0 | | 3 | | 3 |  |  | |  | |  | |  |
| Pshenisnov 2020 ^285^ | | Russia | 2 | | 0 | | 2 | | 0 | | 1 |  |  | |  | | 1 | |  |
| Vashukova 2020 ^286^ | | Russia | 1 | | 0 | | 0 | | 1 | | 1 |  |  | |  | |  | |  |
| Spichak 2020 ^287^ | | Russia | 1 | | 0 | | 0 | |  | |  |  |  | |  | | 1 | |  |
| Olisova 2020 ^288^ | | Russia | 1 | | 0 | | 0 | |  | |  |  |  | | 1 | |  | |  |
| Rusinova 2020 ^289^ | | Russia | 100 | | 0 | | 0 | |  | | 3 |  |  | |  | |  | |  |
| Dondurey 2020 ^290^ | | Russia | 674 | | 1 | | 11 | |  | |  |  |  | |  | |  | |  |
| Meskina 2020 ^291^ | | Russia | 1047 | | 0 | | N/A | |  | |  |  |  | |  | |  | |  |
| Balashov 2020 ^292^ | | Russia | 1 | | 0 | | 0 | | 0 | | 1 |  |  | |  | |  | |  |
| Mazankova 2020 ^293^ | | Russia | 472 | | 0 | | 3 | |  | |  |  |  | |  | |  | |  |
| Uskov 2020 ^204^ | | Russia |  | | 12 | |  | |  | |  |  |  | |  | |  | |  |
| Elbehery 2020 ^295^ | | Saudi Arabia | 1 | | 0 | | 1 | | 0 | | 1 |  |  | |  | |  | |  |
| Algadeeb 2020 ^296^ | | Saudi Arabia | 3 | | 1 | | 1 | | 3 | | 3 |  |  | |  | |  | |  |
| Khalifa 2020 ^297^ | | Saudi Arabia | 1 | | 0 | | 1 | |  | |  |  |  | | 1 | |  | |  |
| Khalifa 2020 ^298^ | | Saudi Arabia | 1 | | 0 | | 0 | |  | |  |  |  | | 1 | |  | |  |
| Alsuwailem 2020 ^299^ | | Saudi Arabia | 1 | | 0 | | 0 | |  | |  | 1 |  | |  | |  | |  |
| Al-Hebshi 2020 ^300^ | | Saudi Arabia | 2 | | 0 | | 0 | |  | |  |  |  | | 2 | |  | |  |
| Haroon 2020 ^301^ | | Saudi Arabia | 1 | | 0 | | 0 | |  | |  |  |  | |  | | 1 | |  |
| Faqeeh 2020 ^302^ | | Saudi Arabia | 1 | | 0 | | 1 | |  | |  |  |  | |  | | 1 | |  |
| Al-Omari 2020 ^303^ | | Saudi Arabia | 16 | | 0 | | 0 | |  | |  |  |  | |  | |  | |  |
| Wong 2020 ^304^ | | Singapore | 3 | | 0 | | 0 | | 0 | | 1 | 2 | 0 | | 0 | | 0 | |  |
| Ng 2020 ^305^ | | Singapore | 1 | | 0 | | 0 | | 0 | | 0 | 0 | 0 | | 0 | | 1 | |  |
| Kam 2020 ^306^ | | Singapore | 1 | | 0 | | 0 | | 0 | | 1 |  |  | |  | |  | |  |
| Li 2020 ^307^ | | Singapore | 39 | | 0 | | 0 | |  | |  | 8 | 17 | | 14 | |  | |  |
| Goussard 2020 ^308^ | | South Africa | 1 | | 0 | | 0 | |  | |  | 1 |  | |  | |  | |  |
| Lee 2020 ^309^ | | South Korea | 2 | | 0 | | 0 | |  | |  |  | 2 | |  | |  | |  |
| Kim 2020 ^310^ | | South Korea | 2 | | 0 | | 0 | |  | |  |  | 2 | |  | |  | |  |
| Yoo 2020 ^311^ | | South Korea | 1 | | 0 | | 0 | |  | |  |  | 1 | |  | |  | |  |
| Cho 2020 ^312^ | | South Korea | 2 | | 0 | | 0 | |  | |  | 1 |  | | 1 | |  | |  |
| Park 2020 ^313^ | | South Korea | 1 | | 0 | | 0 | | 0 | | 0 | 0 | 0 | | 1 | | 0 | |  |
| Han 2020 ^314^ | | South Korea | 12 | | 0 | | 0 | | 1 | | 3 | 1 | 3 | | 4 | | 1 | |  |
| Yoon 2020 ^315^ | | South Korea | 1 | | 0 | | N/A | |  | |  |  |  | |  | |  | |  |
| Han 2020 ^316^ | | South Korea | 91 | | 0 | | N/A | | N/A | | 6 | 13 | 23 | | 31 | | 18 | |  |
| Sanchez Tierraseca 2020 ^317^ | | Spain | 1 | | 0 | | 0 | |  | |  |  |  | | 1 | |  | |  |
| Gine 2020 ^318^ | | Spain | 1 | | 0 | | 1 | |  | |  |  |  | | 1 | |  | |  |
| VelascoPuyo 2020 ^319^ | | Spain | 1 | | 0 | | N/A | | 0 | | 1 |  |  | |  | |  | |  |
| Garcia-Salido 2020 ^320^ | | Spain | 24 | | 0 | | 7 | | N/A | | N/A |  | N/A | | N/A | |  | |  |
| DeCeano-Vivas 2020 ^321^ | | Spain | 58 | | 1 | | 5 | | N/A | | N/A |  |  | |  | |  | |  |
| Giesen 2020 ^322^ | | Spain | 2 | | 0 | | 0 | |  | |  |  |  | |  | |  | |  |
| Melgosa 2020 ^323^ | | Spain | 16 | | 0 | | 0 | | N/A | | 2 |  |  | |  | |  | |  |
| Cabrero-Hernandez 2020 ^324^ | | Spain | 2 | | 0 | | 2 | |  | |  |  | 1 | | 1 | |  | |  |
| deRojas 2020 ^325^ | | Spain | 15 | | 0 | | 0 | | 1 | | 1 | 1 | 5 | | 6 | | 2 | |  |
| VegaHernandez 2020 ^326^ | | Spain | 1 | | 0 | | N/A | |  | |  |  |  | | 1 | |  | |  |
| Díaz 2020 ^327^ | | Spain | 1 | | 0 | | 1 | | 1 | | 1 |  |  | |  | |  | |  |
| Climent 2020 ^328^ | | Spain | 1 | | 1 | | 1 | | 0 | | 1 |  |  | |  | |  | |  |
| Chacón-Aguilar 2020 ^329^ | | Spain | 1 | | 0 | | 0 | | 1 | | 1 | 0 | 0 | | 0 | | 0 | |  |
| Mondejar-Lopez 2020 ^330^ | | Spain | 2 | | 0 | | 0 | |  | |  |  | 1 | |  | | 1 | |  |
| Perez-Suarez 2020 ^331^ | | Spain | 1 | | 0 | | 0 | |  | |  |  |  | |  | | 1 | |  |
| Martinez-Castano 2020 ^332^ | | Spain | 1 | | 0 | | 0 | | 0 | | 1 |  |  | |  | |  | |  |
| Poblador-Plou 2020 ^333^ | | Spain | 6 | | 0 | | N/A | |  | |  |  |  | |  | |  | |  |
| FernandezColomer 2020 ^334^ | | Spain | 40 | | 0 | | 8 | | 40 | | 40 |  |  | |  | |  | |  |
| Vicent 2020 ^335^ | | Spain | 8 | | 1 | | 2 | |  | |  | 1 | 2 | | 5 | |  | |  |
| Faura 2020 ^336^ | | Spain | 47 | | 2 | | 4 | | N/A | | 6 | 9 | 15 | | 13 | | 4 | |  |
| GonzalezCortes 2020 ^337^ | | Spain | 23 | | 0 | | 23 | |  | |  |  |  | |  | |  | |  |
| Gimeno-Costa 2020 ^338^ | | Spain | 1 | | 0 | | 1 | |  | |  |  |  | |  | | 1 | |  |
| Hildenwall 2020 ^339^ | | Sweden | 63 | | 1 | | 1 | |  | |  |  |  | |  | |  | |  |
| Nyholm 2020 ^340^ | | Sweden | 1 | | 0 | | 1 | | 0 | | 1 |  |  | |  | |  | |  |
| Rahmanzade 2020 ^341^ | | Switzerland | 1 | | 0 | | 1 | |  | |  |  |  | |  | | 1 | |  |
| Andre 2020 ^342^ | | Switzerland | 1 | | 0 | | 1 | | 0 | | 1 |  |  | |  | |  | |  |
| Dantonello 2020 ^343^ | | Switzerland | 1 | | 0 | | 0 | |  | |  | 1 |  | |  | |  | |  |
| Posfay-Barbe 2020 ^344^ | | Switzerland | 40 | | 0 | | 0 | |  | |  |  |  | |  | |  | |  |
| Masmejan 2020 ^345^ | | Switzerland | 1 | | 0 | | 0 | |  | |  |  |  | |  | | 1 | |  |
| Yang 2020 ^346^ | | Taiwan | 1 | | 0 | | 0 | |  | |  |  |  | | 1 | |  | |  |
| Anurathapan 2020 ^347^ | | Thailand | 1 | | 0 | | 0 | |  | |  |  |  | |  | |  | |  |
| Moolasart 2020 ^348^ | | Thailand | 1 | | 0 | | 0 | | 0 | | 1 |  |  | |  | |  | |  |
| Wongsawat 2020 ^349^ | | Thailand | 3 | | 0 | | 0 | |  | |  | 1 | 2 | |  | |  | |  |
| Yasri 2020 ^350^ | Thailand | | | 2 | | 0 | | 0 | |  |  | 1 | | 1 | |  | |  | |
| Yarali 2020 ^351^ | Turkey | | | 30 | | 0 | | 0 | |  |  |  | |  | |  | |  | |
| Akcabelen 2020 ^352^ | Turkey | | | 1 | | 0 | | 0 | |  |  |  | |  | | 1 | |  | |
| Kesici 2020 ^353^ | Turkey | | | 1 | | 1 | | 1 | |  |  | 1 | |  | |  | |  | |
| CuraYayla 2020 ^354^ | Turkey | | | 220 | | 2 | | 3 | |  |  |  | |  | |  | |  | |
| Korkmaz 2020 ^355^ | Turkey | | | 81 | | 0 | | 2 | |  |  |  | |  | |  | |  | |
| Yilmaz 2020 ^356^ | Turkey | | | 105 | | 0 | | 3 | |  | 13 |  | |  | |  | |  | |
| Sarbay 2020 ^357^ | Turkey | | | 1 | | 0 | | 0 | |  |  |  | |  | |  | | 1 | |
| Palabiyik 2020 ^358^ | Turkey | | | 59 | | 0 | | 3 | |  |  |  | |  | |  | |  | |
| Koker 2020 ^359^ | Turkey | | | 1 | | 0 | | 0 | |  |  |  | |  | | 1 | |  | |
| Yildirim 2020 ^360^ | Turkey | | | 1 | | 1 | | 1 | |  |  |  | | 1 | |  | |  | |
| Tuncer 2020 ^361^ | Turkey | | | 21 | | 0 | | 0 | |  |  |  | |  | |  | |  | |
| Onal 2020 ^362^ | Turkey | | | 37 | | 0 | | 10 | |  |  |  | |  | |  | |  | |
| Sik 2020 ^363^ | Turkey | | | 45 | | 0 | | 0 | |  | 6 | 10 | | 9 | |  | |  | |
| Gorkem 2020 ^364^ | Turkey | | | 1 | | 0 | | 0 | |  |  |  | |  | |  | | 1 | |
| Soysal 2020 ^365^ | Turkey | | | 237 | | 0 | | 3 | |  | 27 | 63 | |  | |  | | 38 | |
| Kanburoglu 2020 ^366^ | Turkey | | | 37 | | 1 | | 27 | | 37 |  |  | |  | |  | |  | |
| ElDannan 2020 ^367^ | UAE | | | 5 | | 0 | | 0 | |  |  | 1 | | 1 | | 3 | |  | |
| Kirenga 2020 ^368^ | Uganda | | | 8 | | 0 | | 0 | |  |  |  | |  | |  | |  | |
| Swann 2020 ^369^ | UK | | | 651 | | 6 | | 116 | | 53 | 225 | 108 | | 92 | | 94 | | 132 | |
| Gale 2020 ^370^ | UK | | | 66 | | 1 | | 24 | | 66 | 66 |  | |  | |  | |  | |
| Barsoum 2020 ^371^ | UK | | | 1 | | 0 | | 0 | |  |  |  | |  | | 1 | |  | |
| Patel 2020 ^372^ | USA | | | 1 | | 0 | | 1 | |  |  |  | |  | | 1 | |  | |
| Stokes 2020 ^373^ | USA | | | 2 | | 0 | | 2 | |  |  |  | |  | |  | | 2 | |
| Simpson 2020 ^374^ | USA | | | 7 | | 1 | | 5 | |  | 5 |  | |  | |  | | 2 | |
| Samies 2020 ^375^ | USA | | | 1 | | 0 | | 1 | |  |  |  | |  | |  | | 1 | |
| Wahlster 2020 ^376^ | USA | | | 1 | | 0 | | 0 | |  |  |  | |  | |  | | 1 | |
| Diercks 2020 ^377^ | USA | | | 1 | | 0 | | 0 | |  |  | 1 | |  | |  | |  | |
| Shaw 2020 ^378^ | USA | | | 1 | | 0 | | 1 | |  |  | 1 | |  | |  | |  | |
| Wardell 2020 ^379^ | USA | | | 4 | | 0 | | 2 | | 4 | 4 |  | |  | |  | |  | |
| Agha 2020 ^380^ | USA | | | 22 | | 0 | | N/A | |  | 10 | 4 | |  | |  | |  | |
| Mithal 2020 ^381^ | USA | | | 18 | | 0 | | 0 | |  | 19 |  | |  | |  | |  | |
| Rossoff 2020 ^382^ | USA | | | 6 | | 0 | | 0 | |  |  |  | | 4 | |  | | 2 | |
| Mannheim 2020 ^383^ | USA | | | 64 | | 0 | | 7 | |  | 8 | 15 | | 11 | | 10 | |  | |
| Jones 2020 ^384^ | USA | | | 1 | | 0 | | 0 | |  |  |  | |  | | 1 | |  | |
| Danley 2020 ^385^ | USA | | | 1 | | 0 | | 0 | | 0 | 1 |  | |  | |  | |  | |
| White 2020 ^386^ | USA | | | 3 | | 0 | | 0 | | 3 | 3 |  | |  | |  | |  | |
| DeBiasi 2020 ^387^ | USA | | | 165 | | 0 | | 5 | |  | 43 | 26 | | 23 | | 36 | | 37 | |
| Patek 2020 ^388^ | USA | | | 1 | | 0 | | 1 | | 1 | 1 |  | |  | |  | |  | |
| Bush 2020 ^389^ | USA | | | 1 | | 0 | | 0 | |  |  |  | |  | | 1 | |  | |
| Coronado Munoz 2020 ^390^ | USA | | | 1 | | 0 | | 1 | | 1 | 1 |  | |  | |  | |  | |
| Bhumbra 2020 ^391^ | USA | | | 24 | | 1 | | 7 | |  |  |  | |  | |  | |  | |
| Severance 2020 ^392^ | USA | | | 1 | | 0 | | 0 | |  |  | 1 | |  | |  | |  | |
| Mehta 2020 ^393^ | USA | | | 1 | | 0 | | 1 | | 1 | 1 |  | |  | |  | |  | |
| Precit 2020 ^394^ | USA | | | 1 | | 0 | | 1 | | 1 | 1 |  | |  | |  | |  | |
| Dumpa 2020 ^395^ | USA | | | 1 | | 0 | | 0 | | 1 | 1 |  | |  | |  | |  | |
| Pierce-Williams 2020 ^396^ | USA | | | 1 | | 0 | | 0 | | 1 | 1 |  | |  | |  | |  | |
| Lara 2020 ^397^ | USA | | | 1 | | 0 | | 1 | |  |  |  | |  | | 1 | |  | |
| Craver 2020 ^398^ | USA | | | 1 | | 1 | | 0 | |  |  |  | |  | |  | | 1 | |
| Paret 2020 ^399^ | USA | | | 2 | | 0 | | 0 | | 1 | 2 |  | |  | |  | |  | |
| Acker 2020 ^400^ | USA | | | 5 | | 0 | | 1 | | 2 |  |  | | 2 | | 1 | |  | |
| Kalyanaraman 2020 ^401^ | USA | | | 1 | | 0 | | 1 | | 0 | 1 |  | |  | |  | |  | |
| Lee 2020 ^402^ | USA | | | 3 | | 0 | | 0 | |  |  | 1 | |  | |  | | 1 | |
| Lagana 2020 ^403^ | USA | | | 1 | | 0 | | 1 | | 0 | 1 |  | |  | |  | |  | |
| Dugue 2020 ^404^ | USA | | | 1 | | 0 | | 0 | | 0 | 1 | 0 | | 0 | | 0 | | 0 | |
| Derespina 2020 ^405^ | USA | | | 70 | | 2 | | 70 | |  |  |  | |  | |  | |  | |
| Feld 2020 ^406^ | USA | | | 3 | | 0 | | 0 | | 1 | 3 |  | |  | |  | |  | |
| Enner 2020 ^407^ | USA | | | 1 | | 0 | | 1 | |  |  |  | |  | | 1 | |  | |
| Kainth 2020 ^408^ | USA | | | 69 | | 1 | | 23 | |  |  |  | |  | |  | |  | |
| Gefen 2020 ^409^ | USA | | | 1 | | 0 | | 0 | |  |  |  | |  | |  | | 1 | |
| Perez 2020 ^410^ | USA | | | 2 | | 0 | | 0 | |  |  |  | |  | |  | | 2 | |
| Appiah-Kubi 2020 ^411^ | USA | | | 5 | | 0 | | 1 | |  |  | 1 | |  | | 2 | | 2 | |
| Lewis 2020 ^412^ | USA | | | 1 | | 0 | | 1 | |  |  |  | |  | |  | | 1 | |
| Heinz 2020 ^413^ | USA | | | 1 | | 0 | | 1 | | 0 | 1 |  | |  | |  | |  | |
| Krishnan 2020 ^414^ | USA | | | 1 | | 0 | | N/A | |  |  | 1 | |  | |  | |  | |
| Kihira 2020 ^415^ | USA | | | 1 | | N/A | | 1 | |  |  |  | | 1 | |  | |  | |
| Chao 2020 ^416^ | USA | | | 67 | | 1 | | 13 | |  |  |  | |  | |  | |  | |
| Almassi 2020 ^417^ | USA | | |  | |  | |  | |  |  |  | |  | |  | |  | |
| Gampel 2020 ^418^ | USA | | |  | |  | |  | |  |  |  | |  | |  | |  | |
| Choi 2020 ^419^ | USA | | | 1 | | 0 | | 0 | |  |  |  | |  | |  | | 1 | |
| Trogen 2020 ^420^ | USA | | | 1 | | 0 | | 1 | |  |  |  | |  | |  | | 1 | |
| Khoury 2020 ^421^ | USA | | | 6 | | 0 | | N/A | | 6 | 6 |  | |  | |  | |  | |
| McLaren 2020 ^422^ | USA | | | 7 | | 0 | | 0 | | 3 | 7 |  | |  | |  | |  | |
| Salik 2020 ^423^ | USA | | | 1 | | 0 | | 1 | | 1 | 1 |  | |  | |  | |  | |
| McAbee 2020 ^424^ | USA | | | 1 | | 0 | | N/A | | 0 | 0 | 0 | | 0 | | 1 | | 0 | |
| Farley 2020 ^425^ | USA | | | 1 | | 0 | | 1 | |  |  |  | | 1 | |  | |  | |
| SeeTsao 2020 ^426^ | USA | | | 1 | | 0 | | 0 | |  |  |  | |  | | 1 | |  | |
| Otto 2020 ^427^ | USA | | | 424 | | 2 | | 25 | |  |  |  | |  | |  | |  | |
| Kan 2020 ^428^ | USA | | | 1 | | 0 | | 0 | | 0 | 1 |  | |  | |  | |  | |
| Robbins 2020 ^429^ | USA | | | 1 | | 0 | | 0 | | 0 | 1 | 0 | | 0 | | 0 | | 0 | |
| Russell 2020 ^430^ | USA | | | 1 | | 0 | | 0 | |  |  | 1 | |  | |  | |  | |
| Bixler 2020 ^431^ | USA | | | 99 | | 99 | |  | |  | 12 | 11 | | 13 | | 14 | | 49 | |
| Woodworth 2020 ^432^ | USA | | | 16 | | 0 | | 9 | | 16 | 16 |  | |  | |  | |  | |
| Turbin 2020 ^433^ | USA | | | 2 | | 0 | | 0 | |  |  |  | |  | | 1 | | 1 | |
| Alloway 2020 ^434^ | USA | | | 1 | | 0 | | 0 | |  |  |  | | 1 | |  | |  | |
| Sisman 2020 ^435^ | USA | | | 1 | | 0 | | 1 | | 1 | 1 |  | |  | |  | |  | |
| Stokes 2020 ^436^ | USA | | | 69703 | | 46 | | 357 | |  |  |  | |  | |  | |  | |
| Team CDC COVID 2020 ^437^ | USA | | | 745-2572 | | 3 | | 15 | | N/A | 95-398 | 291 | | 388 | | 682 | | 813 | |
| Shekerdemian 2020 ^438^ | USA and Canada | | | 48 | | 2 | | 48 | |  | 8 | 6 | | 7 | |  | |  | |
| Sachdeva 2020 ^439^ | USA and Canada | | | 401 | | 7 | | 401 | |  |  |  | |  | |  | |  | |
| Kim 2020 ^440^ | Uzbekistan | | | 46 | | N/A | | 1 | |  |  |  | |  | |  | |  | |
| Le 2020 ^441^ | Vietnam | | | 1 | | 0 | | 0 | | 0 | 1 |  | |  | |  | |  | |
| Nguyen 2020 ^442^ | Vietnam | | | 6 | | 0 | | 0 | |  |  |  | |  | |  | |  | |
| Al-Waleedi 2020 ^443^ | Yemen | | | 9 | | 1 | | N/A | |  |  |  | |  | |  | |  | |

Abbreviations: N/A, not available

Some studies may contain clinically diagnosed cases without PCR confirmations and cases with MIS-C because of the inability to disaggregate them from PCR-confirmed cases

**References**

1. Khesrani LS, Chana K, Sadar FZ, Dahdouh A, Ladjad Y, Bouguermouh D. Intestinal ischemia secondary to Covid-19. Journal of pediatric surgery case reports 2020. doi: 10.1016/j.epsc.2020.101604
2. Cairoli H, Raiden S, Chiolo MJ, Di L, Sandra F, Fernando C. Patients assisted at the Department of Medicine of a pediatric hospital at the beginning of the COVID-19 pandemic in Buenos Aires, Argentina. Archivos argentinos de pediatria 2020; 118(6): 418-426.
3. Ibrahim, LF, Tosif S, McNab S, Hall S, Lee HJ, Lewena S. et al. SARS-CoV-2 testing and outcomes in the first 30 days after the first case of COVID-19 at an Australian children's hospital EMA - Emergency Medicine Australasia 2020. doi: 10.1111/1742-6723.13550
4. Macartney K, Quinn HE, Pillsbury AJ, et al. Transmission of SARS-CoV-2 in Australian educational settings: a prospective cohort study. The Lancet. Child & adolescent health 2020. doi: 10.1016/S2352-4642(20)30251-0
5. Australia Government, Department of Health. COVID-19, Australia: Epidemiology Report 20 (Fortnightly reporting period ending 5 July 2020) Communicable diseases intelligence (2018) 2020:44. doi: 10.33321/cdi.2020.44.63
6. Presler J, Fill Malfertheiner S, Kabesch M, et al. Postnatal SARS-CoV-2 infection and immunological reaction: A prospective family cohort study. Pediatric allergy and immunology 2020. Doi: 10.1111/pai.13302
7. Saha S, Ahmed ANU, Sarkar PK, et al. The Direct and Indirect Impact of SARS-CoV-2 Infections on Neonates: A Series of 26 Cases in Bangladesh. The Pediatric infectious disease journal 2020; 39(12): e398-e405.
8. Piersigilli F, Carkeek K, Hocq C, et al. COVID-19 in a 26-week preterm neonate. The Lancet Child and Adolescent Health 2020;4(6):476-8. doi: 10.1016/S2352-4642%2820%2930140-1
9. Garau G, Joachim S, Duliere GL, et al. Sudden cardiogenic shock mimicking fulminant myocarditis in a surviving teenager affected by severe acute respiratory syndrome coronavirus 2 infection. ESC heart failure 2020. doi: 10.1002/ehf2.13049
10. Escalera-Antezana JP, Lizon-Ferrufino NF, Maldonado-Alanoca A, et al. Risk factors for mortality in patients with Coronavirus Disease 2019 (COVID-19) in Bolivia: An analysis of the first 107 confirmed cases. Le infezioni in medicina 2020;28(2):238-42.
11. Escalera-Antezana JP, Lizon-Ferrufino NF, Maldonado-Alanoca A, et al. Clinical features of the first cases and a cluster of Coronavirus Disease 2019 (COVID-19) in Bolivia imported from Italy and Spain. Travel Medicine and Infectious Disease 2020:101653. doi: 10.1016/j.tmaid.2020.101653
12. Arapovic J, Skocibusic S. The first two months of the COVID-19 pandemic in Bosnia and Herzegovina: Single-center experience. Bosnian journal of basic medical sciences. 2020;20(3):396-400. doi: 10.17305/bjbms.2020.4838
13. Aragao MT, Santos Junior ELD, Ataide TD, Alves Neto JS, Aragao NVBT. COVID-19 presenting as an exanthematic disease: a case report. Revista da Sociedade Brasileira de Medicina Tropical 2020. doi: 10.1590/0037-8682-0533-2020
14. Frank CHM, Almeida TVR, Marques EA, et al. Guillain-Barre Syndrome Associated with SARS-CoV-2 Infection in a Pediatric Patient. Journal of tropical pediatrics 2020. doi: 10.1093/tropej/fmaa044
15. Schaan CW, Vieira VS, Miller C, et al. HOSPITAL PHYSICAL THERAPY MANAGEMENT IN PEDIATRIC PATIENTS WITH COVID-19: CASE REPORTS. Revista paulista de pediatria : orgao oficial da Sociedade de Pediatria de Sao Paulo 2020. doi: 10.1590/1984-0462/2021/39/2020238
16. Florencio FKZ, Tenorio MO, Junior ARAM, de Lima SG. Aspirin with or without statin in the treatment of endotheliitis, thrombosis, and ischemia in coronavirus disease. Revista da Sociedade Brasileira de Medicina Tropical 2020. doi: 10.1590/0037-8682-0472-2020
17. de Miranda Henriques-Souza AM, de Melo ACMG, de Aguiar Coelho Silva Madeiro B et al. Acute disseminated encephalomyelitis in a COVID-19 pediatric patient. Neuroradiology 2020. doi: 10.1007/s00234-020-02571-0
18. Bezerra RF, Franchi SM, Khader H, et al. COVID-19 as a confounding factor in a child submitted to staged surgical palliation of hypoplastic left heart syndrome: One of the first reports of SARS-CoV-2 infection in patients with congenital heart disease. Journal of Thoracic and Cardiovascular Surgery 2020. doi: 10.1016/j.jtcvs.2020.05.081
19. Carvalho WB, Gibelli MAC, Krebs VLJ, Calil VMLT, Nicolau CM, Johnston C. Neonatal SARS-CoV-2 infection. Clinics (Sao Paulo, Brazil) 2020;75:e1996. doi: 10.6061/clinics/2020/e1996
20. Almeida FJ, Olmos RD, Oliveira DBL, et al. Hematuria associated with SARS-CoV-2 infection in a child. Pediatric Infectious Disease Journal 2020:E161. doi: 10.1097/INF.0000000000002737
21. Giorno EPC, De Paulis M, Sameshima YT et al. Point-of-care lung ultrasound imaging in pediatric COVID-19. The ultrasound journal 2020. doi: 10.1186/s13089-020-00198-z
22. Rabha AC, Oliveira Junior FI, Oliveira TA eet al. CLINICAL MANIFESTATIONS OF CHILDREN AND ADOLESCENTS WITH COVID-19: REPORT OF THE FIRST 115 CASES FROM SABARA HOSPITAL INFANTIL. Revista paulista de pediatria : orgao oficial da Sociedade de Pediatria de Sao Paulo 2020. doi: 10.1590/1984-0462/2021/39/2020305
23. Silva JH, Oliveira EC, Hattori TY, Lemos ERS, Tercas-Trettel ACP. Description of COVID-19 cluster: isolation and testing in asymptomatic individuals as strategies to prevent local dissemination in Mato Grosso state, Brazil, 2020. Epidemiologia e servicos de saude : revista do Sistema Unico de Saude do Brasil 2020. doi: 10.5123/s1679-49742020000400005
24. Prata-Barbosa A, Lima-Setta F, Santos GRD et al. Pediatric patients with COVID-19 admitted to intensive care units in Brazil: a prospective multicenter study. Jornal de pediatria 2020; 96(5): 582-592.
25. Hillesheim D, Tomasi YT, Figueiro TH, Paiva KM. Severe Acute Respiratory Syndrome due to COVID-19 among children and adolescents in Brazil: profile of deaths and hospital lethality as at Epidemiological Week 38, 2020. Epidemiologia e servicos de saude : revista do Sistema Unico de Saude do Brasil 2020. doi: 10.1590/S1679-49742020000500021
26. Magalhaes JJFD, Mendes RPG, Silva CTAD, Silva SJRD, Guarines KM, Pena L. Epidemiological and clinical characteristics of the first 557 successive patients with COVID-19 in Pernambuco state, Northeast Brazil. Travel Medicine and Infectious Disease 2020. doi: 10.1016/j.tmaid.2020.101884
27. de Siqueira Alves Lopes A, Cristina Fontes Vieira S, Lima Santos Porto R, et al. Coronavirus Disease-19 deaths among children and adolescents in an area of Northeast, Brazil: why so many? Tropical medicine & international health: TM & IH 2020. doi: 10.1111/tmi.13529
28. Wong J, Koh WC, Momin RN, Alikhan MF, Fadillah N, Naing L. Probable causes and risk factors for positive SARS-CoV-2 test in recovered patients: Evidence from Brunei Darussalam. Journal of Medical Virology 2020; 92(11): 2847-2851.
29. George S, Ansari MS, Kalliath A, et al. COVID-19 in children in Brunei Darussalam: Higher incidence but mild manifestations. Journal of Medical Virology 2020. doi: 10.1002/jmv.26310
30. Wong J, Chaw L, Koh WC, et al. Epidemiological Investigation of the First 135 COVID-19 Cases in Brunei: Implications for Surveillance, Control, and Travel Restrictions. The American journal of tropical medicine and hygiene 2020; 103(4): 1608-1613.
31. Ladha MA, Dupuis EC. SARS-CoV-2-related chilblains. Canadian Medical Association journal. 2020;192(28):E804. doi: 10.1503/cmaj.201348
32. Kirtsman M, Diambomba Y, Poutanen SM, et al. Probable congenital sars-cov-2 infection in a neonate born to a woman with active sars-cov-2 infection. CMAJ 2020;192(24):E647-E650. doi: 10.1503/cmaj.200821
33. Diaz-Corvillon P, Monckeberg M, Barros A, et al. Routine screening for SARS CoV-2 in unselected pregnant women at delivery. PloS one 2020. doi: 10.1371/journal.pone.0239887
34. Geng J, Yu J, Lu T, Wang Y, Cao Y. A Silent Infection Pandemic of COVID-19: Epidemiological Investigation and Hypothetical Models. The Canadian journal of infectious diseases & medical microbiology 2020:5120253. doi: 10.1155/2020/5120253
35. Xu YH, Dong JH, An WM et al. Clinical and computed tomographic imaging features of novel coronavirus pneumonia caused by SARS-CoV-2. The Journal of infection. 2020; 80(4): 394–400.
36. Zhang W, Tian S, Wang Y, Chen H, Zhang J. Analysis of family cluster infection with Novel Coronavirus Pneumonia. Chinese Journal of Emergency Medicine. 2020; 32(3): 1–4,9.
37. Chang D, Lin M, Wei L et al. Epidemiologic and Clinical Characteristics of Novel Coronavirus Infections Involving 13 Patients Outside Wuhan, China. JAMA. 2020; 323(11): 1092–3.
38. Sun Y, Dong, Y, Wang L et al. Characteristics and prognostic factors of disease severity in patients with COVID-19: The Beijing experience. Journal of Autoimmunity. 2020; 102473.
39. Zhao W, Wang Y, Tang Y, et al. Characteristics of Children With Reactivation of SARS-CoV-2 Infection After Hospital Discharge. Clinical pediatrics 2020;59:929-932. doi: 10.1177/0009922820928057
40. Gao Y, Zhang D, Sui S, Xu R. Clinical Features and Treatment Protocol in Eleven Chinese Children with Mild COVID-19. Indian Journal of Pediatrics 2020. doi: 10.1007/s12098-020-03352-6
41. Liu J, Guo JH, Mei J et al. Clinical and CT features of familiar clustered Novel Corona virus Disease 2019. Radio Practice (Chinese). 2020; 35(4): 437–9.
42. Liu L, Hong Xiuqin, Su Xin, et al. Optimizing screening strategies for coronavirus disease 2019: A study from Middle China. Journal of infection and public health 2020;13(6):868-872. doi: 10.1016/j.jiph.2020.05.003
43. Jiang Y, Niu W, Wang Q, Zhao H, Meng L, Zhang C. Characteristics of a family cluster of Severe Acute Respiratory Syndrome Coronavirus 2 in Henan, China[published online April 18, 2020]. Journal of Infection. doi: 10.1016/j.jinf.2020.04.028
44. Lu T, Pu H. Rare CT manifestions of an asymptomatic girl with COVID-19. Pediatric Pulmonology 2020;55(8): 1882-1883. doi: 10.1002/ppul.24904
45. Bai K. Liu W, Liu C, et al. Clinical Analysis of 25 COVID-19 Infections in Children. The Pediatric infectious disease journal. 2020;39(7):e100-e103. doi: 10.1097/INF.0000000000002740
46. Wei W, Yuan C, Liu X, Zheng M. An infant case of asymptomatic infection of a novel coronavirus with fecal positive nucleic acid. Acad J Chin PLA Med Sch 2020: 41(3).
47. Zhang B, Liu S, Dong Y et al. Positive rectal swabs in young patients recovered from coronavirus disease 2019 (COVID-19) [published online April 28, 2020]. Journal of Infection. doi: 10.1016/j.jinf.2020.04.023
48. Liu S, Cheng F, Yang XY et al. A study of laboratory confirmed cases between laboratory indexes and clinical classification of 342 cases with Corona Virus Disease 2019 in Ezhou[published online April 2, 2020]. Laboratory Medicine. doi: 10.3969/j.issn.1673-8640.2019.00.000
49. Ren JG. Li DY, Wang CF, et al. Positive RT-PCR in urine from an asymptomatic patient with novel coronavirus 2019 infection: a case report. Infectious Diseases 2020;52(8):571-574. doi: 10.1080/23744235.2020.1766105
50. Xu J, Han M, Zhao F, Zhang T, Ma L. Clinical manifestations and sero-immunological characteristics of 155 patients with COVID-19. Chin J Nosocomiol. 2020; 30(15): 961–5.
51. Song X, Wu Q. Pharmaceutical Care Practice of Clinical Pharmacist Participating in Antiviral Therapy for Children with COVID-19. Chin J Mod Appl Pharm 2020; 37: 4.
52. Yang XN, Deng J, Li XF et al. Distribution of Traditional Chinese Medicine Syndromes in Children with Coronavirus Disease 2019: An Analysis of 11 Cases[published online March 19, 2020]. Journal of Guangzhou University of Traditional Chinese Medicine. doi: 10.13359/j.cnki.gzxbtcm.2020.06.002
53. Zhong ZF, Huang J, Yang X, et al. Epidemiological and clinical characteristics of COVID-19 patients in Hengyang, Hunan Province, China. World Journal of Clinical Cases 2020;8(12):2554-2565. doi: 10.12998/wjcc.v8.i12.2554
54. Zhao M, Liu Z, Li Z, Chang C, Zhao X, Geng X. Epidemiological investigation of a cluster associated w ith corona virus disease 2019 in Jinan City, China. Journal of Shandong University 2020;58(4):1-4.
55. Yang L, Li Z, Xu H, et al. Epidemiological and clinical characteristics of 10 children with coronavirus disease (COVID-19) in Jinan City. Journal of Shandong University 2020;58(4):36-9.
56. Gao Q, Hu Y, Dai Z, Xiao F, Wang J, Wu J. The epidemiological characteristics of 2019 novel coronavirus diseases (COVID-19) in Jingmen, Hubei, China. Medicine 2020;99(23):e20605. doi: 10.1097/MD.0000000000020605
57. Zhao C, Xu Y, Zhang X, et al. Public health initiatives from hospitalized patients with COVID-19, China. Journal of Infection and Public Health 2020. doi: 10.1016/j.jiph.2020.06.013
58. Li X, Zhang T, Tang M, Wang Y. COVID-19 pneumonia report of the first child in Yunnan[published online March 31, 2020]. Journal of Chongqing Medical University. doi: 10.13406/j.cnki.cyxb.002419
59. Jiao S, Yang W, Pan D. Analysis of medication for an infant case of mild novel coronavirus pneumonia. Journal of Pediatric Pharmacy 2020;26:4.
60. Chen C, Chen M, Cheng C, et al. A special symptom of olfactory dysfunction in coronavirus disease 2019: report of three cases. Journal of neurovirology 2020;26(3):456-458. doi: 10.1007/s13365-020-00849-w
61. Sun C, Zhang XB, Dai Y, Xu XZ, Zhao J. Clinical analysis of 150 cases of 2019 novel coronavirus infection in Nanyang City, Henan Province. Zhonghua Jie He He Hu Xi Za Zhi. 2020; 43(0): E042–E042.
62. Chen Y, Li X, Jiang Y, Wang Y, Yu W. Clinical features and treatment of 11 cases of COVID-19. Modern Practical Medicine 2020;32(2):150-153,202. doi: 10.3969/j.issn.1671-0800.2020.02.004
63. Qian G, Yang N, Ma A, et al. A COVID-19 Transmission within a family cluster by presymptomatic infectors in China. Clinical infectious diseases 2020. doi: 10.1093/cid/ciaa316
64. Xing YH. Ni W, Wu Q et al. Prolonged viral shedding in feces of pediatric patients with coronavirus disease 2019[published online March 28, 2020]. Journal of microbiology, immunology, and infection. doi: 10.1016/j.jmii.2020.03.021
65. Jia R, Wang X, Liu P, et al. Mild Cytokine Elevation, Moderate CD4+ T Cell Response and Abundant Antibody Production in Children with COVID-19. Virologica Sinica 2020. doi: 10.1007/s12250-020-00265-8
66. Wang F, Qu M, Zhou X, et al. The timeline and risk factors of clinical progression of COVID-19 in Shenzhen, China Journal of Translational Medicine 2020;18(1):270. doi: 10.1186/s12967-020-02423-8
67. Wang Z, Chen X, Lu Y, Chen F, Zhang W. Clinical characteristics and therapeutic procedure for four cases with 2019 novel coronavirus pneumonia receiving combined Chinese and Western medicine treatment. Bioscience trends 2020;14(1):64-68. doi: 10.5582/bst.2020.01030
68. Liu L, Lei X, Xiao X, et al. Epidemiological and Clinical Characteristics of Patients With Coronavirus Disease-2019 in Shiyan City, China. Frontiers in Cellular and Infection Microbiology 2020;10:284. doi: 10.3389/fcimb.2020.00284
69. Ji T, Chen Q, Chen F et al. Clinical Characteristics and Drug Therapy of 4 Cases of Children with Corona Virus Disease 2019. Journal of Pediatric Pharmacy. 2020; 26(4): 24-27.
70. Feng XH, Li ZH, Ke CH, Jiang XQ. Analysis of re-admission of patients with coronavirus disease 2019 after treatment. Chin J Nosocomiol 2020; 30(11): 1610–6.
71. Li C, Luo F, Wu, B.A 3-month-old child with COVID-19: A case report. Medicine 2020;99(23):e20661. doi: 10.1097/MD.0000000000020661
72. Zhang T, Cui X, Zhao X et al. Detectable SARS-CoV-2 viral RNA in feces of three children during recovery period of COVID-19 pneumonia[published online March 29, 2020]. Journal of medical virology. doi: 10.1002/jmv.25795
73. Lei S, Wang G, Zhou G et al. Comparative observation between SARS ⁃CoV ⁃2 gene detection and CT imaging of patients with COVID⁃19. J Mol Diagn Ther 2020; 12(3): 270–274.
74. Wu YT, Liu J, Xu JJ, et al . Neonatal outcome in 29 pregnant women with COVID-19: A retrospective study in Wuhan, China. PLoS medicine 2020;17(7) e1003195. doi: 10.1371/journal.pmed.1003195
75. Wu H, Zhu H, Yuan C, et al . Clinical and Immune Features of Hospitalized Pediatric Patients With Coronavirus Disease 2019 (COVID-19) in Wuhan, China. JAMA network open 2020;3(6):e2010895. doi: 10.1001/jamanetworkopen.2020.10895
76. Liu X, Xie Rong, Li W, et al. Clinical and epidemiological features of 46 children under 1 year old with coronavirus disease 2019 (COVID-19) in Wuhan, China: a descriptive study. The Journal of infectious diseases 2020. doi: 10.1093/infdis/jiaa472
77. Lan L, Xu D, Xia C, Wang S, Yu M, Xu H. Early CT findings of coronavirus disease 2019 (COVID-19) in asymptomatic children: A single-center experience. Korean Journal of Radiology 2020;21(7):919-924. doi: 10.3348/kjr.2020.0231
78. Sun D, Li H, Lu XX et al. Clinical features of severe pediatric patients with coronavirus disease 2019 in Wuhan: a single center's observational study[published online March 19, 2020]. World journal of pediatrics. doi: 10.1007/s12519-020-00354-4
79. Lu X, Zhang L, Du H et al. SARS-CoV-2 Infection in Children[published online March 18, 2020]. The New England journal of medicine. doi: 10.1056/NEJMc2005073
80. Zeng L, Xia S, Yuan W et al. Neonatal Early-Onset Infection With SARS-CoV-2 in 33 Neonates Born to Mothers With COVID-19 in Wuhan, China[published online March 26, 2020]. JAMA pediatrics. doi: 10.1001/jamapediatrics.2020.0878
81. Jiang S, Liu P, Xiong G et al. Coinfection of SARS-CoV-2 and multiple respiratory pathogens in children[published online April 5, 2020]. Clinical chemistry and laboratory medicine. doi: 10.1515/cclm-2020-0434
82. Liu W, Zhang Q, Chen J et al. Detection of Covid-19 in Children in Early January 2020 in Wuhan, China. The New England journal of medicine. 2020; 382(14): 1370–1371.
83. Wang X, Fang J, Zhu Y et al. Clinical characteristics of non-critically ill patients with novel coronavirus infection (COVID-19) in a Fangcang Hospital. Clinical microbiology and infection [published online April 3, 2020]. doi: 10.1016/j.cmi.2020.03.032
84. Wang Y, Zhu F, Wang C, et al. Children hospitalized with severe COVID-19 in Wuhan. Pediatric Infectious Disease Journal 2020:E91-E94. doi: 10.1097/INF.0000000000002739
85. Gao Y, Shi C, Chen Y et al. A cluster of the Corona Virus Disease 2019 caused by incubation period transmission in Wuxi, China[published online April 10, 2020]. The Journal of infection. doi: 10.1016/j.jinf.2020.03.042
86. Zhang GX, Zhang AM, Huang L et al. Twin girls infected with SARS-CoV-2. Chin J Contemp Pediatr. 2020; 22(43): 221–225.
87. Li Y, Cao J, Zhang X, Liu G, Wu X, Wu B. Chest CT imaging characteristics of COVID-19 pneumonia in preschool children: A retrospective study. BMC Pediatrics 2020;20(1):227. doi: 10.1186/s12887-020-02140-7
88. Chen M, Fan P, Liu Z, et al. A SARS-CoV-2 familial cluster infection reveals asymptomatic transmission to children. Journal of Infection and Public Health 2020;13(6):883-886. doi: 10.1016/j.jiph.2020.05.018
89. Zheng H, Yu F, Wang Yi. Epidemiological investigation of corona virus disease 2019 in Xianyang area. Shaanxi Medical Journal 2020; 49(4): 387–391.
90. Li M, Xu M, Zhan W, Han T, Zhang G, Lu Y. Report of the first cases of mother and infant infections with 2019 novel coronavirus in Xinyang City Henan Province[published online February 7, 2020]. Chinese Journal of Infectious Diseases. doi: 10.3760/cma.j.issn.1000-6680.2020.02.000
91. Li F, Yang Y, Wang D, et al. Clinical laboratory monitoring and analysis of COVID-19. Journal of Yanan University 2020;18:1
92. Wu J, Li W, Shi X et al. Early antiviral treatment contributes to alleviate the severity and improve the prognosis of patients with novel coronavirus disease (COVID-19) [published online March 27, 2020]. Journal of internal medicine. doi: 10.1111/joim.13063
93. Li J, Xu Q, Wang Y et al. Analysis in characteristics of asymptomatic infection patients with coronavirus disease 2019 in Yangzhou City of Jiangsu Province[published online March 18, 2020]. Journal of Clinical Medicine in Practice. doi: 10.7619/jcmp.202005003
94. Wu P, Liang L, Chen C, Nie SQ. A child confirmed COVID-19 with only symptoms of conjunctivitis and eyelid dermatitis. Graefe's Archive for Clinical and Experimental Ophthalmology[published online April 24, 2020]. doi:10.1007/s00417-020-04708-6
95. Li Y, Hu Y, Yu Y, et al. Positive result of Sars-Cov-2 in faeces and sputum from discharged patient with COVID-19 in Yiwu, China . Journal of medical virology 2020. doi: 10.1002/jmv.25905
96. Wei Y, Zeng W, Huang X, et al. Clinical characteristics of 276 hospitalized patients with coronavirus disease 2019 in Zengdu District, Hubei Province: a single-center descriptive study. BMC infectious diseases 2020;20(1):549. doi: 10.1186/s12879-020-05252-8
97. Sun M, Xu G, Yang Y, et al. Evidence of mother-to-newborn infection with COVID-19. British journal of anaesthesia 2020;125(2):e245-e247. doi: 10.1016/j.bja.2020.04.066
98. Li Y, Guo F, Cao Y, Li L, Guo YJ. Insight into COVID-2019 for pediatricians. Pediatric Pulmonology. 2020; 55(5): E1–E4.
99. Yu ZY, Xue W, Feng YJ et al. Transport, diagnosis and treatment of a newborn with severe SARS-CoV-2 infection: A case report. Chin J Evid Based Pediatr. 2020; 15(1): 37–41.
100. Tang A, Tong Z, Wang H, et al. Detection of Novel Coronavirus by RT-PCR in Stool Specimen from Asymptomatic Child, China. Emerging infectious diseases 2020;26:6. doi: 10.3201/eid2606.200301
101. Li W, Fang Y, Liao J, et al. Clinical and CT features of the COVID-19 infection: comparison among four different age groups. European Geriatric Medicine 2020. doi: 10.1007/s41999-020-00356-5
102. Zhu Y, Gao ZH, Liu YL et al. Clinical and CT imaging features of 2019 novel coronavirus disease (COVID-19) [published online April 14, 2020]. The Journal of infection. doi: 10.1016/j.jinf.2020.03.033
103. Zheng G, Wang B, Zhang H, et al. Clinical characteristics of acute respiratory syndrome with SARS-CoV-2 infection in children in South China. Pediatric Pulmonology 2020. http://dx.doi.org/10.1002/ppul.24921
104. Fu B, Fu X. Clinical characteristics of 11 asymptomatic patients with COVID-19. Medicina Clinica 2020; 155(2): 87-88. doi: 10.1016/j.medcli.2020.04.013.
105. Yuxia C, Maolu T, Dong H et al. 55-Day-Old Female Infant infected with COVID 19: presenting with pneumonia, liver injury, and heart damage; The Journal of Infectious Diseases; Oxford Academic[published online March 17, 2020]. The Journal of Infectious Diseases. doi: 10.1093/infdis/jiaa113
106. Zhu L, Wang J, Huang R et al. Clinical characteristics of a case series of children with coronavirus disease 2019[published online April 8, 2020]. Pediatric pulmonology. doi: 10.1002/ppul.24767
107. Wu HP, Li BF, Chen X et al. Clinical features of coronavirus disease 2019 in children aged <18 years in Jiangxi, China: an analysis of 23 cases. Chin J Contemp Pediatr. 2020; 22(5): 1–6.
108. Wang D, Ju XL, Xie F et al. Clinical analysis of 31 cases of 2019 novel coronavirus infection in children from six provinces (autonomous region) of northern China. Chinese journal of pediatrics 2020; 58(4): E011.
109. Lin J, Wu M, Wu H, Zhang T, Wu C, Li F. Epidemiological characteristics of coronavirus disease 2019 in Zhejiang Province. Prev Med. 2020; 32(3): 217–221,225.
110. Song R, Han B, Song M, et al. Clinical and epidemiological features of COVID-19 family clusters in Beijing, China. J. infect 2020;81(2):e26-e30.
111. Tan YP, Tan BY, Pan J, Wu J, Zeng SZ, Wei HY. Epidemiologic and clinical characteristics of 10 children with coronavirus disease 2019 in Changsha, China. Journal of clinical virology. 2020; 127: 104353.
112. Xu Y, Li X, Zhu B, et al. Characteristics of pediatric SARS-CoV-2 infection and potential evidence for persistent fecal viral shedding. Nature medicine 2020;26(4):502-505. https://dx.doi.org/10.1038/s41591-020-0817-4
113. Lei P, Huang Z, Liu G, et al. Clinical and computed tomographic (CT) images characteristics in the patients with COVID-19 infection: What should radiologists need to know? Journal of X-ray science and technology 2020. doi: 10.3233/XST-200670
114. Huang L, Zhang X, Wei Z, et al. Rapid asymptomatic transmission of COVID-19 during the incubation period demonstrating strong infectivity in a cluster of youngsters aged 16-23 years outside Wuhan and characteristics of young patients with COVID-19: A prospective contact-tracing study. Journal of Infection 2020. doi: 10.1016/j.jinf.2020.03.006
115. Su L, Ma X, Yu H, et al. The different clinical characteristics of corona virus disease cases between children and their families in China - the character of children with COVID-19. Emerging microbes & infections 2020;9(1):707-713. doi: 10.1080/22221751.2020.1744483
116. Du W, Yu J, Wang H et al. Clinical characteristics of COVID-19 in children compared with adults in Shandong Province, China[published online April 16, 2020]. Infection. doi: 10.1007/s15010-020-01427-2
117. Fan Q, Pan Y, Wu Q et al. Anal swab findings in an infant with COVID-19. Pediatric investigation 2020; 4(1): 48–50.
118. Wang L, Duan Y, Zhang W et al. Epidemiologic and Clinical Characteristics of 26 Cases of COVID-19 Arising from Patient-to-Patient Transmission in Liaocheng, China. Clinical epidemiology. 2020; 12: 387–391.
119. Chan JFW, Yuan S, Kok KH, et al. A familial cluster of pneumonia associated with the 2019 novel coronavirus indicating person-to-person transmission: a study of a family cluster. The Lancet 2020;395(10223):514-523. doi: 10.1016/S0140-6736%2820%2930154-9
120. Guiqing, HE, Sun W, Jing WU, Cai J. Serial Computed Tomography Manifestations in a Child with Coronavirus Disease (COVID-19) Pneumonia. Indian pediatrics 2020. PII: S097475591600158
121. Zheng F, Liao Chun, Fan QH et al. Clinical Characteristics of Children with Coronavirus Disease 2019 in Hubei, China. Current Medical Science. 2020; 40(2): 275–280.
122. Chen Q, Quan B, Li X, et al. A report of clinical diagnosis and treatment of nine cases of coronavirus disease 2019. Journal of Medical Virology 2020. [doi: 10.1002/jmv.25755](http://dx.doi.org/10.1002/jmv.25755)
123. Liu J, Xiao Y, Shen Y et al. Detection of SARS-CoV-2 by RT-PCR in anal from patients who have recovered from coronavirus disease 2019[published online April 14, 2020]. Journal of medical virology. doi: 10.1002/jmv.25875
124. Han YN, Feng ZW, Sun LN et al. A comparative-descriptive analysis of clinical characteristics in 2019-coronavirus-infected children and adults[published online April 6, 2020]. Journal of medical virology. doi: 10.1002/jmv.25835
125. Song W, Li J, Zou N, Guan W, Pan J, Xu W. Clinical features of pediatric patients with coronavirus disease (COVID-19) [published online April 24, 2020]. Journal of Clinical Virology. doi: 10.1016/j.jcv.2020.104377
126. Lou XX, Shi CX, Zhou CC, Tian YS. Three children who recovered from novel coronavirus 2019 pneumonia[published online March 22, 2020]. Journal of Paediatrics and Child Health. doi: 10.1111/jpc.14871
127. Li P, Fu JB, Li KF et al. Transmission of COVID-19 in the terminal stage of incubation period: a familial cluster[published online March 16, 2020]. International journal of infectious diseases. doi: 10.1016/j.ijid.2020.03.027
128. Li W, Cui H, Li K, Fang Y, Li S. Chest computed tomography in children with COVID-19 respiratory infection. Pediatric radiology 2020. doi: 10.1007/s00247-020-04656-7
129. Lin J, Duan, J, Tan T, Fu Z, Dai J. The isolation period should be longer: Lesson from a child infected with SARS-CoV-2 in Chongqing, China. Pediatric pulmonology 2020. doi: 10.1002/ppul.24763
130. Xu R, Du M, Li L, Zhen Z, Wang H, Hu X. CT imaging of one extended family cluster of corona virus disease 2019 (COVID-19) including adolescent patients and "silent infection" Quantitative imaging in medicine and surgery 2020;10(3):800-804. doi: 10.21037/qims.2020.02.13
131. Lo I, Long L, Chon F, Cheong, et al. Evaluation of SARS-CoV-2 RNA shedding in clinical specimens and clinical characteristics of 10 patients with COVID-19 in Macau. International journal of biological sciences 2020;16(10):1698-1707. doi: 10.7150/ijbs.45357
132. Dong Y, Mo X, Hu Y et al. Epidemiological Characteristics of 2143 Pediatric Patients With 2019 Coronavirus Disease in China. Pediatrics. doi: 10.1542/peds.2020-0702
133. Lendorf ME, Boisen MK, Kristensen PL, et al. Thea Characteristics and early outcomes of patients hospitalised for COVID-19 in North Zealand, Denmark. Danish medical journal 2020;67(9).
134. Ali RMM, Ghonimy MBI. Radiological findings spectrum of asymptomatic coronavirus (COVID-19) patients. Egyptian Journal of Radiology and Nuclear Medicine 2020. doi: 10.1186/s43055-020-00266-3
135. Nofal A, Fawzy MM, Sharaf ED, Sherif M, El-Hawary EE. Herpes zoster ophthalmicus in COVID-19 patients. International journal of dermatology 2020: 59(12): 1545-1546.
136. Evelyn C, Susana Z, Mireya G, Rhina D, Ministerio de salud, Instituto Nacional de Salud. Epidemiología y manifestaciones clínicas de COVID-19 en niñez. Ministeriodesalud. 2020; 1: 6–6.
137. Gotzinger F, Santiago-Garcia B, Noguera-Julian A, et al. COVID-19 in children and adolescents in Europe: a multinational, multicentre cohort study. The Lancet Child and Adolescent Health 2020. doi: 10.1016/S2352-4642%2820%2930177-2
138. Martenot A, Labbassi I, Delfils-Stern A et al. Favorable outcomes among neonates not separated from their symptomatic SARS-CoV-2-infected mothers. Pediatric research 2020. doi: 10.1038/s41390-020-01226-3
139. Tchidjou HK, Romeo B. Infant Case of Co-infection with SARS-CoV-2 and Citrobacter koseri Urinary Infection. Journal of tropical pediatrics 2020. doi: 10.1093/tropej/fmaa032
140. Tchidjou HK, Caron F, Ferec A et al. Severe hyperphosphatasemia and severe acute respiratory syndrome coronavirus 2 infection in children. Blood coagulation & fibrinolysis : an international journal in haemostasis and thrombosis 2020; 31(8): 575-577.
141. Danis K, Epaulard O, Benet T, et al. Cluster of coronavirus disease 2019 (Covid-19) in the French Alps, 2020 Clinical infectious diseases 2020. doi: 10.1093/cid/ciaa424
142. Le Roux P, Millardet E, Duquenoy A, Labbe F, Vandendriessche A. Pleuropneumonia resulting from varicella and COVID-19 co-infection in a 10-month-old infant. Archives de pediatrie : organe officiel de la Societe francaise de pediatrie 2020; 27(8): 509-510.
143. Morand A, Roquelaure B, Colson P, et al. Child with liver transplant recovers from COVID-19 infection. A case report. Archives de Pediatrie 2020;27(5):275-276. doi: 10.1016/j.arcped.2020.05.004
144. Aherfi S, Gautret P, Chaudet H, Raoult D, La Scola B. Clusters of COVID-19 associated with Purim celebration in the Jewish community in Marseille, France, March 2020. International journal of infectious diseases : IJID : official publication of the International Society for Infectious Diseases 2020. doi: 10.1016/j.ijid.2020.08.049
145. Colson P, Tissot-Dupont H, Morand A, et al. Children account for a small proportion of diagnoses of SARS-CoV-2 infection and do not exhibit greater viral loads than adults. European journal of clinical microbiology & infectious diseases : official publication of the European Society of Clinical Microbiology 2020: 39(10): 1983-1987.
146. Abasse S, Essabar L, Costin T, et al. Neonatal COVID-19 Pneumonia: Report of the First Case in a Preterm Neonate in Mayotte, an Overseas Department of France. Children (Basel, Switzerland) 2020;7:8. doi: 10.3390/children7080087
147. Heilbronner C, Berteloot L, Tremolieres P, et al. Patients with sickle cell disease and suspected COVID-19 in a paediatric intensive care unit. British Journal of Haematology 2020;190(1):e21-e24. doi: 10.1111/bjh.16802
148. Vivanti AJ, Vauloup-Fellous C, Prevot S, et al. Transplacental transmission of SARS-CoV-2 infection. Nature Communications 2020;11(1):3572. doi: 10.1038/s41467-020-17436-6
149. Nathan N, Prevost B, Corvol H. Atypical presentation of COVID-19 in young infants. The Lancet 2020;395:1481. doi: 10.1016/S0140-6736(20)30980-6
150. Fischer Q, Brillat-Savarin N, Ducrocq Gregory, Ou P. Case report of an isolated myocarditis due to COVID-19 infection in a paediatric patient. European heart journal. Case reports 2020. doi: 10.1093/ehjcr/ytaa180
151. Nathan N, Prevost B, Sileo C et al. The Wide Spectrum of COVID-19 Clinical Presentation in Children. Journal of clinical medicine 2020. doi; 10.3390/jcm9092950
152. Oualha M. Bendavid M. Berteloot L, et al. Severe and fatal forms of COVID-19 in children Archives de pediatrie 2020;27(5):235-238. doi: 10.1016/j.arcped.2020.05.010
153. Gaborieau L, Delestrain C, Bensaid P, et al. Epidemiology and clinical presentation of children hospitalized with SARS-CoV-2 infection in suburbs of Paris. Journal of Clinical Medicine 2020;9:7. doi: 10.3390/jcm9072227
154. Meslin P, Guiomard C, Chouakria M, et al. Coronavirus disease 2019 in newborns and very young infants: A series of six patients in France. Pediatric Infectious Disease Journal 2020:E145-E147. doi: 10.1097/INF.0000000000002743
155. Loron G, Tromeur T, Venot P, et al. COVID-19 Associated With Life-Threatening Apnea in an Infant Born Preterm: A Case Report. Frontiers in pediatrics 2020. doi: 10.3389/fped.2020.00568
156. Nazon C, Velay A, Radosavljevic M, Fafi-Kremer S, Paillard C. Coronavirus disease 2019 3 months after hematopoietic stem cell transplant: A pediatric case report. Pediatric Blood and Cancer 2020;67(9):e28545. doi: 10.1002/pbc.28545
157. Lorenz N, Treptow A, Schmidt S, et al. Neonatal Early-Onset Infection with SARS-CoV-2 in a Newborn Presenting with Encephalitic Symptoms. Pediatric Infectious Disease Journal 2020:E212. doi: 10.1097/INF.0000000000002735
158. Olfe J, Grafmann M, Kozlik-Feldmann R. A teenager with CHD and coronavirus disease 2019. Cardiology in the young 2020. doi: 10.1017/S1047951120002127
159. Wehl G, Laible M, Rauchenzauner M. Co-infection of SARS CoV-2 and influenza A in a Pediatric Patient in Germany. Klinische Padiatrie 2020;232(4):217-218. doi: 10.1055/a-1163-7385
160. Kim-Hellmuth S, Hermann M, Eilenberger J, et al. SARS-CoV-2 triggering severe ARDS and secondary HLH in a 3-year-old child with Down syndrome. Journal of the Pediatric Infectious Diseases Society 2020. doi: 10.1093/jpids/piaa148
161. Farber K, Stabler P, Getzinger T, Uhlig T. Suspected sepsis in a 10-week-old infant and SARS-CoV-2 detection in cerebrospinal fluid and pharynx. Monatsschrift fur Kinderheilkunde 2020. doi: 10.1007/s00112-020-00942-8
162. Koczulla RA, Sczepanski B, Koteczki A, et al et al. SARS-CoV-2 infection in two patients following recent lung transplantation. American Journal of Transplantation 2020. doi: 10.1111/ajt.15998
163. Wolf GK, Glueck T, Huebner J, et al. Clinical and Epidemiological Features of a Family Cluster of Symptomatic and Asymptomatic Severe Acute Respiratory Syndrome Coronavirus 2 Infection. Journal of the Pediatric Infectious Diseases Society 2020;9(3):362-365. doi: 10.1093/jpids/piaa060
164. Armann JP, Diffloth N, Simon A, et al. Hospital Admission in Children and Adolescents With COVID-19. Deutsches Arzteblatt international 2020;117 21:373-374. doi: 10.3238/arztebl.2020.0373
165. Ayisi-Boateng NK, Egblewogbe D, Owusu-Antwi R, Essuman A, Spangenberg K. Exploring the illness experiences amongst families living with 2019 coronavirus disease in Ghana: Three case reports. African journal of primary health care & family medicine 2020; 12(1): e1-e3
166. Maltezou HC, Magaziotou I, Dedoukou X et al. Children and Adolescents With SARS-CoV-2 Infection: Epidemiology, Clinical Course and Viral Loads. The Pediatric infectious disease journal 2020; 39(12): e388-e392
167. Maltezou HC, Vorou R, Papadima K, et al. Transmission dynamics of SARS-CoV-2 within families with children in Greece: A study of 23 clusters. Journal of Medical Virology 2020. doi: 10.1002/jmv.26394
168. Majachani N, Francois JLM, Fernando AK, Zuberi J. A Case of a Newborn Baby Girl Infected with SARS-CoV-2 Due to Transplacental Viral Transmission. The American journal of case reports 2020. doi: 10.12659/AJCR.925766
169. Mak PQ, Chung KS, Wong JSC, Shek CC, Kwan MYW. Anosmia and Ageusia: Not an Uncommon Presentation of COVID-19 Infection in Children and Adolescents. Pediatric Infectious Disease Journal 2020:E199-E200. doi: 10.1097/INF.0000000000002718
170. Rost G, Bartha FA, Bogya N, et al. Early phase of the COVID-19 outbreak in hungary and post-lockdown scenarios. Viruses 2020;12(7):708. doi: 10.3390/v12070708
171. Dosi R, Jain G, Mehta A. Clinical Characteristics, Comorbidities,and Outcome among 365 Patients of Coronavirus Disease 2019 at a Tertiary Care Centre in Central India. The Journal of the Association of Physicians of India 2020; 68(9): 20-23.
172. Singh MV, Shrivastava A, Maurya M, Tripathi A, Sachan R, Siddiqui SA. Vertical Transmission of SARS-CoV-2 from an Asymptomatic Pregnant Woman in India. Journal of tropical pediatrics 2020. doi: 10.1093/tropej/fmaa048
173. Kant R, Zaman K, Shankar P, Yadav R. A preliminary study on contact tracing & transmission chain in a cluster of 17 cases of severe acute respiratory syndrome coronavirus 2 infection in Basti, Uttar Pradesh, India. The Indian journal of medical research 2020. doi: 10.4103/ijmr.IJMR_2914_20
174. Meghwal B, Behera S, Dhariwal AC, Saxena D, Singh R, Kumar S. Insights from COVID-19 cluster containment in Bhilwara District, Rajasthan. Indian journal of public health 2020;64:S177-S182. doi: 10.4103/ijph.IJPH_489_20
175. Mohakud NK, Yerru HJ, Rajguru M, Naik SS. An Assumed Vertical Transmission of SARS-CoV-2 During Pregnancy: A Case Report and Review of Literature. Cureus 2020. doi: 10.7759/cureus.10659
176. Radhakrishnan V, Ovett J, Rajendran A, et al. COVID19 in children with cancer in low- and middle-income countries: Experience from a cancer center in Chennai, India. Pediatric hematology and oncology 2020. doi: 10.1080/08880018.2020.1831113
177. Daniel S, Gadhiya B, Parikh A, Joshi P. COVID-19 in a Child With Diabetic Ketoacidosis: An Instigator, a Deviator or a Spectator. Indian pediatrics 2020. PII: S097475591600211
178. Nayak AH, Kapote DS, Fonseca M, et al. Impact of the Coronavirus Infection in Pregnancy: A Preliminary Study of 141 Patients. Journal of Obstetrics and Gynecology of India. 2020;70(4):256-261. doi: 10.1007/s13224-020-01335-3
179. Kalamdani P, Kalathingal T, Manerkar S, Mondkar J. Clinical Profile of SARS-CoV-2 Infected Neonates From a Tertiary Government Hospital in Mumbai, India. Indian pediatrics 2020. PII: S097475591600250
180. Gupta N, Agrawal S, Ish P. Clinical and epidemiologic profile of the initial COVID-19 patients at a tertiary care centre in India. Monaldi archives for chest disease 2020;90:1. doi: 10.4081/monaldi.2020.1294
181. Bandyopadhyay T, Sharma A, Kumari P, Maria A, Choudhary R. Possible Early Vertical Transmission of COVID-19 from an Infected Pregnant Female to Her Neonate: A Case Report. Journal of tropical pediatrics 2020. doi: 10.1093/tropej/fmaa094
182. Anand P, Yadav A, Debata P, Bachani S, Gupta N, Gera R. Clinical profile, viral load, management and outcome of neonates born to COVID 19 positive mothers: a tertiary care centre experience from India. European journal of pediatrics 2020. doi: 10.1007/s00431-020-03800-7
183. Shankar R, Radhakrishnan N, Dua S, et al. Convalescent plasma to aid in recovery of COVID-19 pneumonia in a child with acute lymphoblastic leukemia. Transfusion and apheresis science : official journal of the World Apheresis Association : official journal of the European Society for Haemapheresis 2020. doi: 10.1016/j.transci.2020.102956
184. Kulkarni R, Rajput U, Dawre R, et al. Early-onset symptomatic neonatal COVID-19 infection with high probability of vertical transmission. Infection 2020. doi: 10.1007/s15010-020-01493-6
185. Kulkarni RK, Kinikar AA, Jadhav T. Fatal Covid-19 in a Malnourished Child with Megaloblastic Anemia. Indian Journal of Pediatrics 2020. doi: 10.1007/s12098-020-03408-7
186. Sarangi B, Reddy VS, Oswal JS, et al. Epidemiological and Clinical Characteristics of COVID-19 in Indian Children in the Initial Phase of the Pandemic. Indian pediatrics 2020. PII: S097475591600218
187. Saraswathi S, Mukhopadhyay A, Shah H, Ranganath TS. Social network analysis of COVID-19 transmission in Karnataka, India. Epidemiology and infection 2020. doi: 10.1017/S095026882000223X
188. Gupta ML, Gothwal S, Gupta RK, et al. Duration of Viral Clearance in Children With SARS-CoV-2 Infection in Rajasthan, India. Indian pediatrics 2020. PII: S097475591600255
189. Laxminarayan R, Wahl B, Dudala SR, et al. Epidemiology and transmission dynamics of COVID-19 in two Indian states. Science (New York, N.Y.) 2020; 370(6517): 691-697.
190. Banerjee S, Guha A, Das A, Nandy M, Monda, R. A Preliminary Report of COVID-19 in Children in India. Indian pediatrics 2020. PII: S097475591600217
191. Marhaeni W, Wijaya AB, Kusumaningtyas P, Mapianto RS. Thalassemic Child Presenting with Anosmia due to COVID-19. Indian Journal of Pediatrics 2020. doi: 10.1007/s12098-020-03370-4
192. Sumarni N, Dewiyanti L, Kusmanto MH, Pramana C. A case of 2019 novel coronavirus infection in a preterm infant with severe respiratory failure. International Journal of Pharmaceutical Research 2020; 12(4): 1935-1938
193. Esmaeili D, Mohammadreza MS, Sorkhi H, et al. COVID-19 and Digestive System in Children: A Retrospective Study. Archives of Iranian medicine 2020; 23(11): 782-786.
194. Mirzaee S, Mohammad M, Goncalves FG, Mohammadifard M, Tavakoli SM, Vossough A. Focal Cerebral Arteriopathy in a COVID-19 Pediatric Patient. Radiology 2020:202197. doi: 10.1148/radiol.2020202197
195. Hashemi SA, Safamanesh S, Ghasemzadeh-Moghaddam H, et al. Report of death in children with SARS-CoV-2 and human metapneumovirus (hMPV) coinfection: Is hMPV the trigger? Journal of Medical Virology 2020. doi: 10.1002/jmv.26401
196. Noghabi ME, Baniasad A, Heidari E, Davoudian N, Malekzadeh F. A 35-day old infant with COVID-19. Iranian Journal of Pediatrics 2020. doi: 10.5812/ijp.103807
197. Gharekhanloo F, Sedighi I, Khazaei S. Variety of radiological findings in a family with COVID-19: a case report. Egyptian Journal of Radiology and Nuclear Medicine 2020. doi: 10.1186/s43055-020-00293-0
198. Norooznezhad AH, Najafi F, Riahi P, Moradinazar M, Shakiba E, Mostafaei S. Primary Symptoms, Comorbidities, and Outcomes of 431 Hospitalized Patients with Confirmative RT-PCR Results for COVID-19. The American journal of tropical medicine and hygiene 2020; 103(2): 834-837.
199. Mamishi S, Heydari H, Aziz-Ahari A, et al. Novel coronavirus disease 2019 (COVID-19) outbreak in children in Iran: Atypical CT manifestations and mortality risk of severe COVID-19 infection. Journal of microbiology, immunology, and infection = Wei mian yu gan ran za zhi 2020. doi: 10.1016/j.jmii.2020.07.019
200. Moradveisi B, Ataee P, Ghaffarieh A, Karimi A, Fattahi N, Nasseri K. Diarrhea as a Presenting Symptom of Coronavirus Disease 2019 in Children. Advanced biomedical research 2020. doi: 10.4103/abr.abr_90_20
201. Navaeifar MR, Poudineh Ghazaghi M, Shahbaznejad L, et al. Fever with Rash is One of the First Presentations of COVID-19 in Children: A Case Report. International medical case reports journal 2020. doi: 10.2147/IMCRJ.S262935
202. Malekhosseini SA, Nikoupour H, Gholami S, et al. A Report of 85 Cases of COVID-19 and Abdominal Transplantation From a Single Center: What Are the Associated Factors With Death Among Organ Transplantation Patients. Transplantation 2020. doi: [10.1097/TP.0000000000003470](https://dx.doi.org/10.1097/TP.0000000000003470)
203. Emami A, Fadakar N, Akbari A, et al. Seizure in patients with COVID-19. Neurological sciences : official journal of the Italian Neurological Society and of the Italian Society of Clinical Neurophysiology 2020; 41(11): 3057-3061.
204. Nikoupour H, Kazemi K, Arasteh P, et al. Pediatric liver transplantation and COVID-19: a case report. BMC surgery 2020; 20(1): 224.
205. Saeed A, Shorafa E. Status epilepticus as a first presentation of COVID-19 infection in a 3 years old boy; Case report and review the literature. IDCases 2020. doi: 10.1016/j.idcr.2020.e00942
206. Saeed A, Shorafa E, Shahramian I, Afshari M, Salahifard M, Parooie F. An 11-year-old boy infected with COVID-19 with presentation of acute liver failure. Hepatitis Monthly 2020. doi: 10.5812/hepatmon.104415
207. Ekbatani MS, Hassani SA, Tahernia L, et al. Atypical and novel presentations of Coronavirus Disease 2019: a case series of three children. British journal of biomedical science 2020. doi: 10.1080/09674845.2020.1785102
208. Nikpouraghdam M, Jalali FA, Alishiri GH, et al. Epidemiological characteristics of coronavirus disease 2019 (COVID-19) patients in IRAN: A single center study. Journal of clinical virology : the official publication of the Pan American Society for Clinical Virology 2020;127:104378. doi: 10.1016/j.jcv.2020.104378
209. Jafari R, Cegolon L, Torkaman M, et al. A 6 months old infant with fever, dyspnea and poor feeding, diagnosed with COVID-19. Travel Medicine and Infectious Disease 2020; 36:101789. doi: 10.1016/j.tmaid.2020.101789
210. Eghbali A, Shokrollahi S, Mahdavi NS, Mahdavi SA, Dabbagh A. COVID-19 in pediatric patients: A case series. Journal of Cellular and Molecular Anesthesia 2020; 5: 1. doi: 10.22037/jcma.v5i1.29690
211. Karimi A, Tabatabaei SR, Khalili M, et al. COVID-19 and chickenpox as a viral co-infection in a 12-year-old patient, a case report. Archives of Pediatric Infectious Diseases 2020; 8: 3. doi: 10.5812/pedinfect.105591
212. Rabizadeh S, Hajmiri M, Rajab A, Emadi Kouchak H, Nakhjavani M. Severe diabetic ketoacidosis and coronavirus disease 2019 (COVID-19) infection in a teenage patient with newly diagnosed diabetes. Journal of pediatric endocrinology & metabolism : JPEM 2020; 33(9): 1241-1243
213. Rohani P, Karimi A, Tabatabaie SR, Khalili M, Sayyari A. Protein losing enteropathy and pneumatosis intestinalis in a child with COVID 19 infection. Journal of pediatric surgery case reports 2020. doi: [10.1016/j.epsc.2020.101667](https://dx.doi.org/10.1016/j.epsc.2020.101667)
214. Haji Esmaeil Memar E, Mamishi S, Sharifzadeh Ekbatani M, et al. Fulminant hepatic failure: A rare and devastating manifestation of Coronavirus disease 2019 in an 11-year-old boy. Archives de pediatrie : organe officiel de la Societe francaise de pediatrie 2020; 27(8): 502-505.
215. Dorgalaleh A, Baghaipour MR, Tabibian S, et al. Gastrointestinal bleeding in a newborn infant with congenital factor X deficiency and COVID-19-A common clinical feature between a rare disorder and a new, common infection. International journal of laboratory hematology 2020. doi: 10.1111/ijlh.13318
216. Mahmoudi S, Rostamyan M, Aziz-Ahari A, Pourakbari B, Ghaffari M, Mamishi S. The First Confirmed Case of Coronavirus Disease 2019 (COVID-19) in an Adolescent with Abdominal Pain in Iran. Tanaffos 2020; 19(2): 156-159.
217. Kalantari H, Tabrizi AHH, Foroohi F. Determination of COVID-19 prevalence with regards to age range of patients referring to the hospitals located in western Tehran, Iran. Gene Reports 2020. doi: 10.1016/j.genrep.2020.100910
218. Nasimfar A, Valizadeh R, Nanbakhsh M. Do we trust the polymerase chain reaction test result in children to diagnose COVID-19? A case report of COVID-19. Open Access Macedonian Journal of Medical Sciences. 2020;8(T1):32-35. doi: 10.3889/oamjms.2020.4742
219. Soltani J, Sedighi I, Shalchi Z, Sami G, Moradveisi B, Nahidi S. Pediatric coronavirus disease 2019 (COVID-19): An insight from west of Iran. Northern clinics of Istanbul. 2020;7(3):284-291. doi: 10.14744/nci.2020.90277
220. Schwartz DA, Mohagheghi P, Beigi B, Zafaranloo N, Moshfegh F, Yazdani A. Spectrum of neonatal COVID-19 in Iran: 19 infants with SARS-CoV-2 perinatal infections with varying test results, clinical findings and outcomes. The journal of maternal-fetal & neonatal medicine : the official journal of the European Association of Perinatal Medicine, the Federation of Asia and Oceania Perinatal Societies, the International Society of Perinatal Obstetricians 2020. doi: 10.1080/14767058.2020.1797672
221. Hashemi SA, Safamanesh S, Zadeh-Moghaddam HG, Ghafouri M, Amir, A. High incidence of SARS-CoV-2 and influenza A virus (H1N1) co-infection in dead patients in Northeastern Iran. Journal of medical virology 2020. doi: 10.1002/jmv.26364
222. Merza MA, Haleem AAA, Mohammed HM, Abdulah DM. COVID-19 outbreak in Iraqi Kurdistan: The first report characterizing epidemiological, clinical, laboratory, and radiological findings of the disease. Diabetes and Metabolic Syndrome: Clinical Research and Reviews 2020;14(4):547-554. doi: 10.1016/j.dsx.2020.04.047
223. Hussein NR, Naqid IA, Saleem ZSM. A retrospective descriptive study characterizing coronavirus disease epidemiology among people in the Kurdistan Region, Iraq. Mediterranean Journal of Hematology and Infectious Diseases 2020. doi: 10.4084/MJHID.2020.061
224. Linnane N, Cox DW, James A. A case of COVID-19 in a patient with a univentricular heart post total cavopulmonary connection (Fontan) surgery. Cardiology in the young 2020. doi: 10.1017/S1047951120001882
225. Jacobi M, Lancrei HM, Brosh-Nissimov T, Yeshayahu Y. PURPURONA: A NOVEL REPORT OF COVID-19-RELATED HENOCH-SCHONLEIN PURPURA IN A CHILD. The Pediatric infectious disease journal 2020. doi: 10.1097/INF.0000000000003001
226. Lopian M, Kashani-Ligumsky L, Czeiger S, et al. Safety of vaginal delivery in women infected with COVID-19. Pediatrics and neonatology 2020. doi: 10.1016/j.pedneo.2020.10.010
227. Stein-Zamir C, Abramson N, Shoob H, et al. A large COVID-19 outbreak in a high school 10 days after schools' reopening, Israel, May 2020. Euro surveillance 2020; 25: 29. doi: 10.2807/1560-7917.ES.2020.25.29.2001352
228. Nunziata F, Bruzzese E, Poeta M, et al. Health-care organization for the management and surveillance of SARS-CoV-2 infection in children during pandemic in Campania region, Italy. Italian journal of pediatrics 2020. doi: 10.1186/s13052-020-00928-y
229. Vergine G, Fantini M, Marchetti F, et al. Home Management of Children With COVID-19 in the Emilia-Romagna Region, Italy. Frontiers in pediatrics 2020. doi: 10.3389/fped.2020.575290
230. Parri N, Lenge M, Cantoni B, et al. COVID-19 in 17 Italian Pediatric Emergency Departments. Pediatrics 2020. doi: 10.1542/peds.2020-1235
231. Parri N, Lenge M, Buonsenso D. Children with Covid-19 in pediatric emergency departments in Italy. New England Journal of Medicine 2020;383(2):187-190. doi: 10.1056/NEJMc2007617
232. Bellino S, Punzo O, Rota MC, et al. COVID-19 Disease Severity Risk Factors for Pediatric Patients in Italy. Pediatrics 2020. doi: 10.1542/peds.2020-009399
233. Garazzino S, Montagnani C, Dona D, et al. Multicentre Italian study of SARS-CoV-2 infection in children and adolescents, preliminary data as at 10 April 2020. Eurosurveillance 2020;25:18. doi: 10.2807/1560-7917.ES.2020.25.18.2000600
234. Irie Y, Nakae H, Fukui S. Three mild cases of coronavirus disease 2019 treated with saikatsugekito, a Japanese herbal medicine. Traditional and Kampo Medicine 2020. doi: 10.1002/tkm2.1261
235. Kawamura Y, Higashimoto Y, Miura H, et al. Immune response against SARS-CoV-2 in pediatric patients including young infants. Journal of medical virology 2020. doi: 10.1002/jmv.26493
236. Kakuya F, Okubo H, Fujiyasu H, Wakabayashi I, Syouji M, Kinebuchi T. The first pediatric patients with coronavirus disease 2019 (COVID-19) in Japan; The risk of co-infection with other respiratory viruses. Japanese journal of infectious diseases 2020. doi: 10.7883/yoken.JJID.2020.181
237. Ishii M, Terai H, Kabata H, et al. Clinical characteristics of 345 patients with coronavirus disease 2019 in Japan: A multicenter retrospective study. Journal of Infection 2020. doi: 10.1016/j.jinf.2020.08.052
238. Kasuga Y, Kanezawa K, Shimizu S, et al. What is the difference in severity of pediatric coronavirus disease 2019? Acta paediatrica (Oslo, Norway : 1992) 2020. doi: 10.1111/apa.15499
239. Higuchi T, Nishida T, Iwahashi H, et al. Early Clinical Factors Predicting the Development of Critical Disease in Japanese Patients with COVID-19: A Single-Center, Retrospective, Observational Study. Journal of medical virology 2020. doi: 10.1002/jmv.26599
240. Sano F, Yagasaki H, Kojika S, et al. Severe Apparent Life-threatening Event (ALTE) in an Infant with SARS-CoV 2 Infection. Japanese journal of infectious diseases 2020. doi: 10.7883/yoken.JJID.2020.572
241. Alkhatatbeh H, Alzaghari D, Alkhashman A, Azab M, Edwan GMA, Abufaraj M. Does severe acute respiratory syndrome coronavirus-2 (SARS-CoV-2) cause orchitis in patients with coronavirus disease 2019 (COVID-19)? Arab Journal of Urology 2020; 18(3): 129-133.
242. Kilani MM, Odeh MM, Shalabi M, Al Qassieh R, Al-Tamimi M. Clinical and laboratory characteristics of SARS-CoV2-infected paediatric patients in Jordan: serial RT-PCR testing until discharge. Paediatrics and international child health 2020. doi: 10.1080/20469047.2020.1804733
243. Yusef D, Hayajneh W, Awad S, et al. Large outbreak of coronavirus disease among wedding attendees, Jordan. Emerging Infectious Diseases 2020; 26(9): 2165-2167.
244. Semenova Y, Glushkova N, Pivina L, et al. Epidemiological Characteristics and Forecast of COVID-19 Outbreak in the Republic of Kazakhstan. Journal of Korean medical science 2020; 35(24): e227. doi: 10.3346/jkms.2020.35.e227
245. Savic D, Alsheikh TM, Alhaj AK, et al. Ruptured cerebral pseudoaneurysm in an adolescent as an early onset of COVID-19 infection: case report. Acta Neurochirurgica 2020. doi: 10.1007/s00701-020-04510-7
246. Ayed A, Embaireeg A, Benawadh A, et al. Maternal and perinatal characteristics and outcomes of pregnancies complicated with COVID-19 in Kuwait. BMC pregnancy and childbirth 2020. doi: 10.1186/s12884-020-03461-2
247. Alsharrah D, Alhaddad F, Alyaseen M, et al. Clinical characteristics of pediatric SARS-CoV-2 infection and coronavirus disease 2019 (COVID-19) in Kuwait. Journal of medical virology 2020. doi: 10.1002/jmv.26684
248. Mansour A, Atoui R, Kanso K, Mohsen R, Fares Y, Fares J. First Case of an Infant with COVID-19 in the Middle East. Cureus 2020;12(4):e7520. doi: 10.7759/cureus.7520
249. Oberweis ML, Codreanu A, Boehm W, et al. Pediatric Life-Threatening Coronavirus Disease 2019 With Myocarditis. The Pediatric infectious disease journal 2020;39(7):e147-e149. doi: 10.1097/INF.0000000000002744
250. Vee ST, Muhamad DB, Nordin NB, Ali NFBN, Abdullah NB. Clinical characteristics of severe acute respiratory syndrome coronavirus 2 (Sars-cov2) patients in hospital tengku ampuan afzan. Medical Journal of Malaysia 2020; 70(5): 479-484.
251. See KC, Liew SM, Ng David CE, et al. COVID-19: Four Paediatric Cases in Malaysia. International journal of infectious diseases 2020. doi: 10.1016/j.ijid.2020.03.049
252. Castano-Jaramillo LM, Yamazaki-Nakashimada MA, Scheffler Mendoza SC, Bustamante-Ogando JC, Espinosa-Padilla SE, Lugo Reyes SO. A male infant with COVID-19 in the context of ARPC1B deficiency. Pediatric allergy and immunology : official publication of the European Society of Pediatric Allergy and Immunology 2020. doi: 10.1111/pai.13322
253. Flores V, Miranda R, Merino L, et al. SARS-CoV-2 infection in children with febrile neutropenia. Annals of Hematology 2020;99(8):1941-1942. doi: 10.1007/s00277-020-04115-1
254. Hinojosa-Velasco A, de Oca PVBM, Garcia-Sosa LE, et al. A case report of newborn infant with severe COVID-19 in Mexico: Detection of SARS-CoV-2 in human breast milk and stool. International journal of infectious diseases : IJID : official publication of the International Society for Infectious Diseases 2020. doi: 10.1016/j.ijid.2020.08.055
255. Olivar-Lopez V, Leyva-Barrera A, Lopez-Martinez B, Parra-Ortega I, Marquez-Gonzalez H. Clinical risk profile associated with SARS-CoV-2 infection and complications in the emergency area of a pediatric COVID-19 center. Boletin medico del Hospital Infantil de Mexico 2020; 77(5): 221-227
256. Lahfaoui M, Azizi M, Elbakkaoui M, El AR, Kamaoui I, Benhaddou H. Syndrome de détresse respiratoire aiguë secondaire à une infection à SARS-COV-2 chez un nourrisson. [published online April 27, 2020] Revue des Maladies Respiratoires. doi: 10.1016/j.rmr.2020.04.009
257. Chekhlabi N, Kettani CE, Haoudar A, et al. The epidemiological and clinical profile of covid-19 in children: Moroccan experience of the cheikh khalifa university center. Pan African Medical Journal 2020;35:2(Supplement). doi: 10.11604/pamj.2020.35.2.23571
258. Nassih H, El Fakiri K, Sab IA. Absence of Evidence of Transmission of Coronavirus Disease 2019 from a Young Child to Mother Despite Prolonged Contact. Indian journal of pediatrics 2020. doi: 10.1007/s12098-020-03382-0
259. Fakiri KE, Nassih H, Sab IA, Draiss G, Bouskraoui M. Epidemiology and Clinical Features of Coronavirus Disease 2019 in Moroccan Children. Indian pediatrics 2020. https://www.indianpediatrics.net/COVID29.03.2020/RP-00207.pdf
260. Janah H, Zinebi A, Elbenaye J. Atypical erythema multiforme palmar plaques lesions due to Sars-Cov-2. Journal of the European Academy of Dermatology and Venereology 2020;34(8):e373-e375. doi: 10.1111/jdv.16623
261. de Sanctis,V, Canatan D, Corrons JLV, et al. Preliminary Data on COVID-19 in Patients with Hemoglobinopathies: A Multicentre ICET-A Study. Mediterranean journal of hematology and infectious diseases 2020;12(1):e2020046. doi: 10.4084/MJHID.2020.046
262. Caro-Dominguez P, Shelmerdine SC, Toso S, et al. Thoracic imaging of coronavirus disease 2019 (COVID-19) in children: a series of 91 cases. Pediatric Radiology 2020. doi: 10.1007/s00247-020-04747-5
263. Sola A, Rodriguez S, Cardetti M, Davila C. Perinatal COVID-19 in Latin America. 2020;44:e47. doi: 10.26633/RPSP.2020.47
264. Slaats MALJ, Versteylen M, Gast KB, at al. Case report of a neonate with high viral SARSCoV-2 loads and long-term virus shedding. Journal of infection and public health 2020. doi: 10.1016/j.jiph.2020.10.013
265. Soumana A, Samaila A, Moustapha LM, et al. A Fatal Case of COVID-19 in an Infant with Severe Acute Malnutrition Admitted to a Paediatric Ward in Niger. Case reports in pediatrics 2020. doi: 10.1155/2020/8847415
266. Ibrahim OR, Suleiman BM, Sanda A, et al. Covid-19 in children: A case series from Nigeria. Pan African Medical Journal 2020;35:2 (Supplement 2). doi: 10.11604/pamj.2020.35.2.23597
267. Adedeji IA, Abdu YM, Bashir MF, et al. Profile of children with COVID-19 infection: a cross sectional study from North-East Nigeria. The Pan African medical journal 2020. doi: 10.11604/pamj.supp.2020.35.145.25350
268. Stordal K, Bakken IJ, Greve-Isdahl M, et al. SARS-CoV-2 in children and adolescents in Norway: confirmed infection, hospitalisations and underlying conditions. Tidsskrift for den Norske laegeforening : tidsskrift for praktisk medicin, ny raekke 2020. doi: 10.4045/tidsskr.20.0457
269. Kristoffersen AW, Knudsen PK, Moller T, et al. SARS-CoV-2 infection in an infant with severe dilated cardiomyopathy. Cardiology in the young 2020. doi: 10.1017/S1047951120004060
270. Alwardi TH, Ramdas V, Al Yahmadi M, et al. Is Vertical Transmission of SARS-CoV-2 Infection Possible in Preterm Triplet Pregnancy? A Case Series. The Pediatric infectious disease journal 2020; 39(12): e456-e458.
271. Moazzam Z, Salim A, Ashraf A, Jehan F, Arshad M. Intussusception in an infant as a manifestation of COVID-19. Journal of Pediatric Surgery Case Reports 2020;59:101533. doi: 10.1016/j.epsc.2020.101533
272. Alzamora MC, Paredes T, Caceres D, Webb CM, Valdez LM, La Rosa M. Severe COVID-19 during Pregnancy and Possible Vertical Transmission. American journal of perinatology 2020. doi: 10.1055/s-0040-1710050
273. Conto-Palomino NM, Cabrera-Bueno ML, Vargas-Ponce KG, Rondon-Abuhadba EA, Atamari-Anahui N. Encephalitis associated with COVID-19 in a 13-year-old girl: A case report. Medwave 2020. doi: 10.5867/medwave.2020.07.7984
274. Rodriguez-Portilla RE, Munaico-Abanto ME, Paredes-Zevallos RP, Quispe-Flores GA. Cardiac tamponade and intracerebral hemorrhage in a child with COVID-19: Case report. Revista de la Facultad de Medicina Humana 2020; 20(4): 743-747.
275. Montoya J, Ugaz C, Alarcon S, et al. COVID-19 in pediatric cancer patients in a resource-limited setting: National data from Peru. Pediatric Blood and Cancer 2020. doi: 10.1002/pbc.28610
276. Gujski M, Raciborski F, Jankowski M, Nowicka PM, Rakocy K, Pinkas J. Epidemiological analysis of the first 1389 cases of COVID-19 in Poland: A preliminary report. Medical Science Monitor 2020;26:e924702-1. doi: 10.12659/MSM.924702
277. Jarmolinski T, Matkowska-Kocjan A, Rosa M, et al. SARS-CoV-2 viral clearance during bone marrow aplasia after allogeneic hematopoietic stem cell transplantation-A case report. Pediatric transplantation 2020. doi: 10.1111/petr.13875
278. Correia CR, Marcal M, Vieira F, et al. Congenital SARS-CoV-2 Infection in a Neonate With Severe Acute Respiratory Syndrome. The Pediatric infectious disease journal 2020; 39(12): e439-e443.
279. Picao de Carvalho C, Castro C, Sampaio Graca I, et al. Case Series of 103 Children with SARS-CoV-2 Infection in Portugal. Acta medica portuguesa 2020. doi: 10.20344/amp.14537
280. Omrani AS, Almaslamani MA, Daghfal J, et al. The first consecutive 5000 patients with Coronavirus Disease 2019 from Qatar; a nation-wide cohort study. BMC infectious diseases 2020. doi: 10.1186/s12879-020-05511-8
281. Al Kuwari HM, Abdul Rahim HF, Abu-Raddad LJ, et al. Epidemiological investigation of the first 5685 cases of SARS-CoV-2 infection in Qatar, 28 February-18 April 2020. BMJ open 2020. doi: 10.1136/bmjopen-2020-040428
282. Soliman AT, Al-Amri M, Alleethy K, Alaaraj N, Hamed N, De Sanctis V. Newly-onset type 1 diabetes mellitus precipitated by COVID-19 in an 8-month-old infant. Acta bio-medica : Atenei Parmensis 2020. doi: 10.23750/abm.v91i3.10074
283. Marginean CO, Melit LE, Sasaran MO. The Discrepancies of COVID-19 Clinical Spectrum Between Infancy and Adolescence - Two Case Reports and a Review of the Literature. Frontiers in pediatrics 2020. doi: 10.3389/fped.2020.577174
284. Dima M, Enatescu I, Craina M, Petre I, Iacob ER, Iacob D. First neonates with severe acute respiratory syndrome coronavirus 2 infection in Romania: Three case reports. Medicine 2020. doi; 10.1097/MD.0000000000021284
285. Pshenisnov KV, Aleksandrovich YS, Kaziakhmedov VA, Kostik MM, Kondrashev IA. Features of current and intensive therapy of new coronavirus infection in children with comorbidities (clinical cases). Jurnal Infektologii 2020; 12(3): 80-89.
286. Vashukova MA, Zinserling VA, Semenova NY, Lugovskaya NA, Narkevich TA, Sukhanova YV. Is perinatal CoVID-19 possible: first results. Jurnal Infektologii 2020; 12(3): 51-55.
287. Spichak II, Moiseeva LV, Karimova IP, Babik RK, Kireeva GN. Reported case of asymptomatic SARS-CoV-2-carriage in the child with severe chronic disease. Pediatricheskaya Farmakologiya 2020. doi: 10.15690/pf.v17i3.2125
288. Olisova OY, Anpilogova EM, Shnakhova LM. Cutaneous manifestations in COVID-19: a skin rash in a child. Dermatologic Therapy 2020. doi: 10.1111/dth.13712
289. Rusinova DS, Nikonov EL, Glazkova LSN-BGP, Vishneva EA, Kaytukova EV, Privalova TE. Primary Observational Results on Children Who Have Been Exposed to COVID-19 in Moscow. Pediatricheskaya Farmakologiya 2020; 17(2): 95-102.
290. Dondurey EA, Isankina LN, Afanasyeva OI, et al. Characteristics of CoVID-19 in children: The first experience in the hospital of st. Petersburg. Jurnal Infektologii 2020; 12(3): 56-63.
291. Meskina ER. Preliminary Clinical and Epidemiological Analysis of the First 1,000 Pediatric COVID-19 Cases in Moscow Region. Journal of Microbiology, Epidemiology and Immunobiology 2020; 97(3): 202-213.
292. Balashov D, Trakhtman P, Livshits A, et al. SARS-CoV-2 convalescent plasma therapy in pediatric patient after hematopoietic stem cell transplantation. Transfusion and apheresis science : official journal of the World Apheresis Association : official journal of the European Society for Haemapheresis 2020. doi: 10.1016/j.transci.2020.102983
293. Mazankova LN, Osmanov IM, Samitova ER, Nedostoev AA, Kotenko SO, Kulichkina OS. The course of coronavirus pneumonia in a teenager from the focus of infection. Children Infections 2020; 19(3): 68-72.
294. Uskov AN, Lobzin YV, Rychkova SV, et al. Course of a new coronavirus infection in children: some aspects of monitoring and analysis of mortality. Jurnal Infektologii 2020. doi: 10.22625/2072-6732-2020-12-3-12-20
295. Elbehery M, Munshi FA, Alzahrani A, Bakhsh M, Alariefy M. COVID-19 in an Intrauterine Growth Restriction (IUGR) Infant with Congenital Heart Disease: Case Report and Literature Review. Cureus 2020. doi: 10.7759/cureus.10294
296. Algadeeb KB, AlMousa HH, AlKadhem SM, Alduhilan MO, Almatawah Y. A Novel Case of Severe Respiratory Symptoms and Persistent Pulmonary Hypertension in a Saudi Neonate With SARS-CoV-2 Infection. Cureus 2020. doi; 10.7759/cureus.10472
297. Khalifa M, Zakaria F, Ragab Y, et al. Guillain-Barre Syndrome Associated with SARS-CoV-2 Detection and a COVID-19 Infection in a Child Journal of the Pediatric Infectious Diseases Society 2020. doi: 10.1093/jpids/piaa086
298. Khalifa M, Zakaria F, Ragab Y, et al. Guillain-Barre Syndrome Associated With Severe Acute Respiratory Syndrome Coronavirus 2 Detection and Coronavirus Disease 2019 in a Child. Journal of the Pediatric Infectious Diseases Society 2020; 9(4): 510-513
299. Alsuwailem AB, Turkistani R,Alomari M. Complicated Appendicitis in a Pediatric Patient With COVID-19: A Case Report. Cureus 2020;12(6):e8677. doi: 10.7759/cureus.8677
300. Al-Hebshi A, Zolaly M, Alshengeti A, et al. A Saudi family with sickle cell disease presented with acute crises and COVID-19 infection Pediatric Blood and Cancer 2020;67(9):e28547. doi: 10.1002/pbc.28547
301. Haroon A, Alnassani M, Aljurf M, et al. COVID-19 post hematopoietic cell transplant, a report of 11 cases from a single center. Mediterranean Journal of Hematology and Infectious Diseases 2020. doi: 10.4084/MJHID.2020.070
302. Faqeeh S, Madkhali R. Acute reversible renal failure requiring temporary dialysis in a patient with COVID-19. Radiology Case Reports 2020; 15(11): 2449-2452.
303. Al-Omari A, Alhuqbani WN, Zaidi ARZ, et al. Clinical characteristics of non-intensive care unit COVID-19 patients in Saudi Arabia: A descriptive cross-sectional study. Journal of infection and public health 2020; 13(11): 1639-1644.
304. Wong JEL, Leo YS, Tan CC. COVID-19 in Singapore - Current Experience: Critical Global Issues That Require Attention and Action. JAMA 2020; 323(13): 1243-1244. doi: 10.1001/jama.2020.2467
305. Ng OT, Marimuthu K, Chia PY, et al. SARS-CoV-2 infection among travelers returning from Wuhan, China New England Journal of Medicine.2020; 382(15): 1476-1478. doi: 10.1056/NEJMc2003100
306. Kam KQ, Yung CF, Cui L, et al. A Well Infant With Coronavirus Disease 2019 With High Viral Load. Clinical infectious diseases : an official publication of the Infectious Diseases Society of America 2020; 71(15): 847-849.
307. Li J, Thoon KC, Chong CY, et al. Comparative Analysis of Symptomatic and Asymptomatic SARS-CoV-2 Infection in Children. Annals of the Academy of Medicine, Singapore 2020; 49(8): 530-537
308. Goussard P, Solomons RS, Andronikou S, Mfingwana L, Verhagen LM, Rabie H. COVID-19 in a child with tuberculous airway compression . Pediatric Pulmonology 2020. doi: 10.1002/ppul.24927
309. Lee M, Eun Y, Park K, Heo J, Son H. Follow-up investigation of asymptomatic COVID-19 cases at diagnosis in Busan, Korea. Epidemiology and health 2020. doi: 10.4178/epih.e2020046
310. Kim SE, Jeong HS, Yu Y, et al. Viral kinetics of SARS-CoV-2 in asymptomatic carriers and presymptomatic patients. International Journal of Infectious Diseases 2020;95:441-443. doi: 10.1016/j.ijid.2020.04.083
311. Yoo SY, Lee Y, Lee GH, Kim DH. Reactivation of SARS-CoV-2 after recovery. Pediatrics International 2020;62(7):879-881. doi: 10.1111/ped.14312
312. Cho YJ, Song KH, Lee Y, et al. Lung ultrasound for early diagnosis and severity assessment of pneumonia in patients with coronavirus disease 2019. Korean Journal of Internal Medicine 2020;35(4):771-781. doi: 10.3904/KJIM.2020.180
313. Park JY, Han MS, Park KU, Kim JY, Choi EH. First Pediatric Case of Coronavirus Disease 2019 in Korea. Journal of Korean medical science 2020;35(11):e124. doi: 10.3346/jkms.2020.35.e124
314. Han MS, Seong M, Kim N, et al. Viral RNA Load in Mildly Symptomatic and Asymptomatic Children with COVID-19, Seoul. Emerging infectious diseases 2020;26:10. doi: 10.3201/eid2610.202449
315. Yoon Y, Choi GJ, Kim JY, et al. Childcare Exposure to Severe Acute Respiratory Syndrome Coronavirus 2 for 4-Year-Old Presymptomatic Child, South Korea. Emerging infectious diseases 2020. doi; 10.3201/eid2702.203189
316. Han MS, Choi EH, Chang SH, et al. Clinical Characteristics and Viral RNA Detection in Children With Coronavirus Disease 2019 in the Republic of Korea. JAMA pediatrics 2020. doi: 10.1001/jamapediatrics.2020.3988
317. Sanchez TM, Balmaseda SEM, Hernandez-Berto T. Exclusive gastrointestinal manifestations as a form of presentation of coronavirus infection (COVID-19). Anales de Pediatría 2020;93(1):72-73. doi: 10.1016/j.anpedi.2020.04.021
318. Gine C, Lain A, Garcia L, Lopez M. Thoracoscopic Bullectomy for Persistent Air Leak in a 14-Year-Old Child with COVID-19 Bilateral Pulmonary Disease. Journal of laparoendoscopic & advanced surgical techniques. Part A 2020. doi: 10.1089/lap.2020.0289
319. Velasco PP, Moreno L, Diaz de Heredia C, Riviere JG, Soler PP. Tocilizumab in a child with acute lymphoblastic leukaemia and COVID-19-related cytokine release syndrome. Anales de Pediatria 2020;93(2):132-133. doi: 10.1016/j.anpedi.2020.05.002
320. Garcia-Salido A, Leoz-Gordillo I, Martinez de Azagra-Garde A, et al. Children in Critical Care Due to Severe Acute Respiratory Syndrome Coronavirus 2 Infection: Experience in a Spanish Hospital. Pediatric critical care medicine 2020. doi: 10.1097/PCC.0000000000002475
321. De Ceano-Vivas M, Martin-Espin I, Del Rosal T, et al. SARS-CoV-2 infection in ambulatory and hospitalised Spanish children. Archives of Disease in Childhood 2020;105(8):808-809. doi: 10.1136/archdischild-2020-319366
322. Giesen C, Diez-Izquierdo L, Saa-Requejo CM, et al. Epidemiological characteristics of the COVID-19 outbreak in a secondary hospital in Spain. American journal of infection control 2020. doi: 10.1016/j.ajic.2020.07.014
323. Melgosa M, Madrid A, Alvarez O, et al. SARS-CoV-2 infection in Spanish children with chronic kidney pathologies. Pediatric nephrology (Berlin, Germany) 2020;35(8):1521-1524. doi: 10.1007/s00467-020-04597-1
324. Cabrero-Hernandez M, Garcia-Salido A, Leoz-Gordillo I, et al. Severe SARS-CoV-2 Infection in Children with Suspected Acute Abdomen: A Case Series from a Tertiary Hospital in Spain. Pediatric Infectious Disease Journal 2020: E195-E198. doi: 10.1097/INF.0000000000002777
325. de Rojas T, Perez-Martinez A, Cela E, et al. COVID-19 infection in children and adolescents with cancer in Madrid. Pediatric Blood and Cancer 2020;67(7): e28397. doi: 10.1002/pbc.28397
326. Vega HP, Borges RY, Ortega SE, et al. Autoimmune Hemolytic Anemia in a Pediatric Patient With Severe Acute Respiratory Syndrome Coronavirus 2 Infection. The Pediatric infectious disease journal 2020. doi: 10.1097/INF.0000000000002809
327. DÃ­az CA, Maestro ML, Pumarega MTM Antin BF, Alonso CP. First case of neonatal infection due to COVID 19 in Spain. Anales de Pediatrica 2020. doi: 10.1016/j.anpede.2020.03.002
328. Climent FJ, Calvo C, Garcia-Guereta L, et al. Fatal outcome of COVID-19 disease in a 5-month infant with comorbidities. Revista Espanola de Cardiologia 2020. doi: 10.1016/j.rec.2020.04.011
329. Rocío C, Juana MO, Isabel S, Carolina G, Juan L, B. Pérez-Moneo. COVID-19: Fever syndrome and neurological symptoms in a neonate. An Pediatr 2020. doi: 10.1016/j.anpede.2020.04.001
330. Mondejar-Lopez P, Quintana-Gallego E, Giron-Moreno RM, et al. Impact of SARS-CoV-2 infection in patients with cystic fibrosis in Spain: Incidence and results of the national CF-COVID19-Spain survey. Respiratory Medicine 2020;170:106062. doi: 10.1016/j.rmed.2020.106062
331. Perez-Suarez B, Martinez-Menchon T, Cutillas-Marco E. Skin findings in the COVID-19 pandemic in the Region of Murcia. Medicina Clínica 2020;155(1):41-42. doi: 10.1016/j.medcli.2020.05.001
332. Martinez-Castano I, Calabuig-Barbero E, Gonzalvez-Pinera J, Lopez-Ayala JM. COVID-19 infection is a diagnostic challenge in infants with ileocecal intussusception. Pediatric Emergency Care 2020;36(6): e368. doi: 10.1097/PEC.0000000000002155
333. Poblador-Plou B, Carmona-Pirez J, Ioakeim-Skoufa I, et al. Baseline chronic comorbidity and mortality in laboratory-confirmed COVID-19 cases: Results from the PRECOVID study in Spain. International Journal of Environmental Research and Public Health 2020;17:14. doi: 10.3390/ijerph17145171
334. Fernandez Colomer B, Sanchez-Luna M, de Alba Romero C, et al. Neonatal Infection Due to SARS-CoV-2: An Epidemiological Study in Spain. Frontiers in pediatrics 2020. doi: 10.3389/fped.2020.580584
335. Vicent MG, Martinez AP, Trabazo del Castillo M, et al. COVID-19 in pediatric hematopoietic stem cell transplantation: The experience of Spanish Group of Transplant (GETMON/GETH) Pediatric Blood and Cancer. 2020; 67(9): e28514. doi: 10.1002/pbc.28514
336. Faura A, Rives S, Lassaletta A, et al. Initial report on Spanish pediatric oncologic, hematologic, and post stem cell transplantation patients during SARS-CoV-2 pandemic. Pediatric Blood and Cancer 2020; 67(9): e28557. doi: 10.1002/pbc.28557
337. Gonzalez CR, Garcia-Salido A, Roca PD, Slocker BM, de Carlos Vicente JC. A multicenter national survey of children with SARS-CoV-2 infection admitted to Spanish Pediatric Intensive Care Units. Intensive care medicine 2020. doi: 10.1007/s00134-020-06146-8
338. Gimeno-Costa R, Barrios M, Heredia T, Garcia C, de Hevia L. COVID-19 respiratory failure: ECMO support for children and young adult patients. Anales de pediatria (Barcelona, Spain : 2003) 2020. doi: 10.1016/j.anpedi.2020.05.007
339. Hildenwall H, Luthander J, Rhedin S, et al. Paediatric COVID-19 admissions in a region with open schools during the two first months of the pandemic. Acta Paediatrica, International Journal of Paediatrics 2020. doi: 10.1111/apa.15432
340. Nyholm S, Edner A, Myrelid A, Janols H, Dorenberg R, Diderholm B. Invasive mechanical ventilation in a former preterm infant with COVID-19. Acta Paediatrica, International Journal of Paediatrics 2020. doi: 10.1111/apa.15437
341. Rahmanzade R, Rahmanzadeh R, Hashemian S, Mohammad R. Respiratory Distress in Postanesthesia Care Unit: First Presentation of Coronavirus Disease 2019 in a 17-Year-Old Girl: A Case Report. A&A practice 2020; 14(7): e01227. doi: 10.1213/XAA.0000000000001227
342. Andre MC, Patzug K, Bielicki J, Gualco G, Busi I, Hammer J. Can SARS-CoV-2 cause life-threatening bronchiolitis in infants? Pediatric Pulmonology 2020; 55(11): 2842-2843
343. Dantonello TM, Kartal-Kaess M, Aebi C, et al. SARS-CoV-2 Infection During Induction Chemotherapy in a Child With High-risk T-Cell Acute Lymphoblastic Leukemia (T-ALL). Journal of pediatric hematology/oncology 2020. doi; 10.1097/MPH.0000000000001943
344. Posfay-Barbe KM, Wagner N, Gauthey M, et al. COVID-19 in Children and the Dynamics of Infection in Families. Pediatrics 2020. doi: 10.1542/peds.2020-1576
345. Masmejan S, Pomar L, Favre G, et al. Vertical transmission and materno-fetal outcomes in 13 patients with coronavirus disease 2019. Clinical microbiology and infection : the official publication of the European Society of Clinical Microbiology and Infectious Diseases 2020; 26(11): 1585-1587.
346. Yang MC, Hung PP, Wu YK, Peng MY, Chao YC, Su WL. A three-generation family cluster with COVID-19 infection: should quarantine be prolonged? Public Health 2020;185:31-33. doi: 10.1016/j.puhe.2020.05.043
347. Anurathapan U, Apiwattanakul N, Pakakasama S, et al. Hematopoietic stem cell transplantation from an infected SARS-CoV2 donor sibling. Bone Marrow Transplantation 2020; 55(12): 2359-2360.
348. Moolasart V, Wongsawat J, Phokhom P, Thienthong V et al. Favipiravir-based regimen for coronavirus disease 2019 pneumonia for a 47-day-old male newborn. SAGE open medical case reports 2020. doi; 10.1177/2050313X20964046
349. Wongsawat J, Moolasart V, Srikirin P, et al. Risk of novel coronavirus 2019 transmission from children to caregivers: A case series . Journal of Paediatrics and Child Health 2020; 56(6): 984-985. doi: 10.1111/jpc.14965
350. Yasri Sora, Wiwanitkit Viroj. Clinical features in pediatric COVID-19. Pediatric pulmonology 2020; 55(5): 1097. doi: 10.1002/ppul.24737
351. Yarali N, Akcabelen YM, Unal Y, Parlakay AN. Hematological parameters and peripheral blood morphologic abnormalities in children with COVID-19. Pediatric blood & cancer 2020: e28596. doi: 10.1002/pbc.28596
352. Akcabelen YM, Koca YA, Parlakay AN, Yarali N. COVID-19 in a child with severe aplastic anemia. Pediatric Blood and Cancer 2020;67(8):e28443. doi: 10.1002/pbc.28443
353. Kesici S, Aykan HH, Orhan D, Bayrakci B. Fulminant COVID-19-related myocarditis in an infant. European heart journal 2020. doi: 10.1093/eurheartj/ehaa515
354. Cura Yayla BC, Ozsurekci Y, Aykac K, et al. Characteristics and Management of Children with COVID-19 in Turkey. Balkan medical journal 2020; 37(6): 341-347.
355. Korkmaz MF, Ture E, Dorum BA, Kilic ZB. The Epidemiological and Clinical Characteristics of 81 Children with COVID-19 in a Pandemic Hospital in Turkey: an Observational Cohort Study. Journal of Korean medical science 2020; 35(25): e236. doi: 10.3346/jkms.2020.35.e236
356. Yilmaz K, Gozupirinccioglu A, Aktar F, et al. Evaluation of the novel coronavirus disease in Turkish children: Preliminary outcomes. Pediatric pulmonology 2020; 55(12): 3587-3594.
357. Sarbay H, Atay A, Malbora B. COVID-19 Infection in a Child With Thalassemia Major After Hematopoietic Stem Cell Transplant. Journal of pediatric hematology/oncology 2020. doi: 10.1097/MPH.0000000000001895
358. Palabiyik F, Kokurcan SO, Hatipoglu N, Cebeci SO, Inci E. Imaging of COVID-19 pneumonia in children. The British journal of radiology 2020:20200647. doi: 10.1259/bjr.20200647
359. Koker O, Demirkan FG, Kayaalp G, Cakmak F, et al. Does immunosuppressive treatment entail an additional risk for children with rheumatic diseases? A survey-based study in the era of COVID-19. Rheumatology International 2020. doi: 10.1007/s00296-020-04663-9
360. Yildirim AI, Karaagac AT. COVID-19 in a Young Girl with Restrictive Cardiomyopathy and Chronic Lung Disease. Indian Pediatrics 2020;57(6):577-578. doi: 10.1007/s13312-020-1863-1
361. Tuncer T, Karaci M, Boga A, Durmaz H, Guven S. QT Interval Evaluation Associated With Use of Hydroxychloroquine with Combined Use of Azithromycin Among Hospitalized Children Positive for COVID-19. Cardiology in the young 2020. doi: 10.1017/S1047951120002425
362. Onal P, Kilinc AA, Aygun F, Durak C, Cokugras H. COVID-19 IN Turkey: A tertiary center experience. Pediatrics international : official journal of the Japan Pediatric Society 2020. doi: 10.1111/ped.14549
363. Sik N, Ozlu C, Karaoglu Asrak H, et al. Evaluation of SARS-CoV-2 PCR Positive Cases in the Pediatric Emergency Department. Mikrobiyoloji bulteni 2020; 54(4): 629-637.
364. Gorkem SB, Cetin BS. COVID-19 pneumonia in a Turkish child presenting with abdominal complaints and reversed halo sign on thorax CT. Diagnostic and interventional radiology (Ankara, Turkey) 2020. doi :10.5152/dir.2020.20361
365. Soysal A, Gonullu E, Arslan H, et al. Comparison of clinical and laboratory features and treatment options of 237 Comparison of clinical and laboratory features and treatment options of 237 symptomatic and asymptomatic children infected with SARS-CoV-2 in the early phase of the COVID-19 pande. Japanese journal of infectious diseases 2020. doi: 10.7883/yoken.JJID.2020.781
366. Kanburoglu MK, Tayman C, Oncel MY, et al. A Multicentered Study on Epidemiologic and Clinical Characteristics of 37 Neonates With Community-acquired COVID-19. The Pediatric infectious disease journal 2020; 39(10): e297-e302.
367. El Dannan H, Al Hassani M, Ramsi M. Clinical course of COVID-19 among immunocompromised children: a clinical case series. BMJ case reports 2020. doi: 10.1136/bcr-2020-237804
368. Kirenga B, Muttamba W, Kayongo A, at al. Characteristics and outcomes of admitted patients infected with SARS-CoV-2 in Uganda. BMJ Open Respiratory Research 2020. doi: 10.1136/bmjresp-2020-000646
369. Swann OV, Holden KA, Turtle L, et al. Clinical characteristics of children and young people admitted to hospital with covid-19 in United Kingdom: prospective multicentre observational cohort study. BMJ (Clinical research ed.) 2020. doi: 10.1136/bmj.m3249
370. Gale C, Quigley MA, Placzek A, et al. Characteristics and outcomes of neonatal SARS-CoV-2 infection in the UK: a prospective national cohort study using active surveillance. The Lancet. Child & adolescent health 2020. doi: 10.1016/S2352-4642(20)30342-4
371. Barsoum Z. Pediatric Asthma & Coronavirus (COVID-19)-Clinical Presentation in an Asthmatic Child-Case Report. SN Comprehensive Clinical Medicine 2020; 2(6): 700-702. doi: 10.1007/s42399-020-00310-3
372. Patel PA, Chandrakasan S, Mickells GE, Yildirim I, Kao CM, Bennett CM. Severe Pediatric COVID-19 Presenting With Respiratory Failure and Severe Thrombocytopenia. Pediatrics 2020; 146: 1. doi: 10.1542/peds.2020-1437
373. Stokes CL, Patel PA, Sabnis HS, Mitchell SG, Yildirim IB, Pauly MG. Severe COVID-19 disease in two pediatric oncology patients. Pediatric Blood and Cancer 2020; 67(9): e28432. doi: 10.1002/pbc.28432
374. Simpson M, Collins C, Nash DB, Panesar LE, Oster ME. COVID-19 Infection in Children with Pre-existing Heart Disease. The Journal of pediatrics 2020. doi: 10.1016/j.jpeds.2020.07.069
375. Samies,NL, Pinninti S, James SH. Rhabdomyolysis and Acute Renal Failure in an Adolescent with COVID-19. Journal of the Pediatric Infectious Diseases Society 2020. doi: 10.1093/jpids/piaa083
376. Wahlster L, Weichert-Leahey N, Trissal M, Grace RF, Sankaran VG. COVID-19 presenting with autoimmune hemolytic anemia in the setting of underlying immune dysregulation. Pediatric Blood and Cancer 2020; 67(9): e28382. doi: 10.1002/pbc.28382
377. Diercks GR, Park BJ, Myers LB, Kwolek CJ. Asymptomatic COVID-19 infection in a child with nasal foreign body. International Journal of Pediatric Otorhinolaryngology. 2020; 135: 110092.
378. Shaw R, Tighe N, Odegard KC, Alexander P, Emani S, Yuki K. Intubation precautions in a pediatric patient with severe COVID-19 . Journal of Pediatric Surgery Case Reports. 2020; 58: 101495.
379. Wardell H, Campbell JI, VanderPluym C, Dixit A. SARS-CoV-2 Infection in Febrile Neonates. Journal of the Pediatric Infectious Diseases Society. [doi: 10.1093/jpids/piaa084](https://dx.doi.org/10.1093/jpids/piaa084)
380. Agha R, Kojaoghlanian T, Avner JR. Initial Observations of COVID-19 in US Children. Hospital pediatrics. doi: 10.1542/hpeds.2020-000257
381. Mithal LB, Machut KZ, Muller WJ, Kociolek LK. SARS-CoV-2 Infection in Infants Less than 90 Days Old. The Journal of pediatrics 2020. doi: 10.1016/j.jpeds.2020.06.047
382. Rossoff J, Patel AB, Muscat E, Kociolek LK, Muller WJ. Benign course of SARS-CoV-2 infection in a series of pediatric oncology patients. Pediatric Blood and Cancer 2020; 67(9): e28504. doi: 10.1002/pbc.28504
383. Mannheim J, Gretsch S, Layden JE, Fricchione MJ. Characteristics of Hospitalized Pediatric COVID-19 Cases - Chicago, Illinois, March - April 2020. Journal of the Pediatric Infectious Diseases Society 2020. doi: 10.1093/jpids/piaa070
384. Jones BA, Slater BJ. Non-operative management of acute appendicitis in a pediatric patient with concomitant COVID-19 infection. Journal of Pediatric Surgery Case Reports 2020; 59: 101512. doi: 10.1016/j.epsc.2020.101512
385. Danley K, Kent P. 4-month-old boy coinfected with COVID-19 and adenovirus. BMJ Case Reports 2020; 13(6): e236264. doi: 10.1136/bcr-2020-236264
386. White A, Mukherjee P, Stremming J, et al. Neonates Hospitalized with Community-Acquired SARS-CoV-2 in a Colorado Neonatal Intensive Care Unit. Neonatology 2020. doi: 10.1159/000508962
387. DeBiasi RL, Song X, Delaney M, et al. Severe Coronavirus Disease-2019 in Children and Young Adults in the Washington, DC, Metropolitan Region. Journal of Pediatrics 2020; 223: 199. doi: 10.1016/j.jpeds.2020.05.007
388. Patek P, Corcoran J, Adams L, Khandhar P. SARS-CoV-2 Infection in a 2-Week-Old Male With Neutropenia. Clinical pediatrics. doi: [10.1177/0009922820920014](https://dx.doi.org/10.1177/0009922820920014)
389. Bush R, Johns F, Acharya R, Upadhyay K. Mild COVID-19 in a pediatric renal transplant recipient. American Journal of Transplantation 2020. doi: 10.1111/ajt.16003
390. Coronado M, Alvaro N, Upulie MM et al. Late-Onset Neonatal Sepsis in a Patient with Covid-19. The New England journal of medicine. doi: [10.1056/NEJMc2010614](https://dx.doi.org/10.1056/NEJMc2010614)
391. Bhumbra S, Malin S, Kirkpatrick L, et al. Clinical Features of Critical Coronavirus Disease 2019 in Children. Pediatric critical care medicine 2020. doi: 10.1097/PCC.0000000000002511
392. Severance TS, Rahim MQ, French J,et al. COVID-19 and hereditary spherocytosis: A recipe for hemolysis. Pediatric Blood and Cance 2020. doi: 10.1002/pbc.28548
393. Mehta H, Ivanovic S, Cronin A et al. Novel coronavirus-related acute respiratory distress syndrome in a patient with twin pregnancy: A case report. Case Reports in Women's Health. 2020; 27: e00220.
394. Precit MR, Yee R, Anand V, Mongkolrattanothai K, Pandey U, Dien Bard J. A Case Report of Neonatal Acute Respiratory Failure Due to Severe Acute Respiratory Syndrome Coronavirus-2. Journal of the Pediatric Infectious Diseases Society 2020; 9(3): 390-392. doi: 10.1093/jpids/piaa064
395. Dumpa V, Kamity R, Vinci AN, Noyola E, Noor A. Neonatal Coronavirus 2019 (COVID-19) Infection: A Case Report and Review of Literature. Cureus. 2020; 12(5): e8165.
396. Pierce-Williams RAM, Burd J, Felder L, et al. Clinical course of severe and critical coronavirus disease 2019 in hospitalized pregnancies: a United States cohort study. American Journal of Obstetrics and Gynecology MFM 2020. doi: 10.1016/j.ajogmf.2020.100134
397. Lara D, Young T, Del Toro K, et al. Acute Fulminant Myocarditis in a Pediatric Patient With COVID-19 Infection. Pediatrics 2020; 146: 2. doi: 10.1542/peds.2020-1509
398. Craver R, Huber S, Sandomirsky M, McKenna D, Schieffelin J, Finger L. Fatal Eosinophilic Myocarditis in a Healthy 17-Year-Old Male with Severe Acute Respiratory Syndrome Coronavirus 2 (SARS-CoV-2c). Fetal and Pediatric Pathology 2020. doi: 10.1080/15513815.2020.1761491
399. Paret M, Lighter J, Pellett M et al. SARS-CoV-2 infection (COVID-19) in febrile infants without respiratory distress. Clinical infectious diseases : an official publication of the Infectious Diseases Society of America 2020. doi: [10.1093/cid/ciaa452](https://dx.doi.org/10.1093/cid/ciaa452)
400. Acker KP, Schertz K, Abramson EL, DeLaMora P, Salvatore CM, Han JY. Infectious Diseases Diagnoses of Children Admitted With Symptoms of Coronavirus Disease 2019 During an Outbreak in New York City. Clinical Pediatrics 2020. doi: [10.1177/0009922820944399](http://dx.doi.org/10.1177/0009922820944399)
401. Kalyanaraman M, McQueen D, Morparia K, Bergel M. ARDS in an ex-premature infant with bronchopulmonary dysplasia and COVID-19 Pediatric pulmonology 2020. doi: [10.1002/ppul.24989](https://dx.doi.org/10.1002/ppul.24989)
402. Lee H, Mantell BS, Richmond ME et al. Varying Presentations of COVID-19 in Young Heart Transplant Recipients: A Case Series Pediatric Transplantation 2020. doi: [10.1111/petr.13780](http://dx.doi.org/10.1111/petr.13780)
403. Lagana SM, De MS, Lee MJ, et al. COVID-19 Associated Hepatitis Complicating Recent Living Donor Liver Transplantation. Archives of pathology & laboratory medicine 2020. doi: [10.5858/arpa.2020-0186-SA](https://dx.doi.org/10.5858/arpa.2020-0186-SA)
404. Dugue R, Cay-Martinez KC, Thakur Ki et al. Neurologic manifestations in an infant with COVID-19. Neurology 2020. doi: [10.1212/WNL.0000000000009653](https://dx.doi.org/10.1212/WNL.0000000000009653)
405. Derespina KR, Kaushik S, Plichta A et al. Clinical Manifestations and Outcomes of Critically Ill Children and Adolescents with COVID-19 in New York City. The Journal of pediatrics 2020. doi: [10.1016/j.jpeds.2020.07.039](http://dx.doi.org/10.1016/j.jpeds.2020.07.039)
406. Feld L, Belfer J, Kabra R et al. A case series of the 2019 novel coronavirus (SARS-CoV-2) in 3 febrile infants in New York. Pediatrics 2020; 146(1): e20201056.
407. Enner S, Hormozdyaran S, Varughese R, et al. Central Apnea in an Adolescent With COVID-19. Pediatric Neurology 2020. doi: [10.1016/j.pediatrneurol.2020.05.012](http://dx.doi.org/10.1016/j.pediatrneurol.2020.05.012)
408. Kainth MK, Goenka PK, Williamson KA, et al. Early Experience of COVID-19 in a US Children' Hospital, Pediatrics 2020. doi: [10.1542/peds.2020-003186](http://dx.doi.org/10.1542/peds.2020-003186)
409. Gefen AM, Palumbo N, Nathan SK, Singer PS, Castellanos-Reyes LJ, Sethna CB. Pediatric COVID-19-associated rhabdomyolysis: a case report. Pediatric Nephrology. 2020; 35(8): 1517-1520.
410. Perez A, Kogan-Liberman D, Sheflin-Findling S, Raizner A, Ahuja KL, Ovchinsky N. Presentation of SARS-CoV-2 Infection As Cholestatic Jaundice in Two Healthy Adolescents. The Journal of pediatrics 2020. doi: [10.1016/j.jpeds.2020.07.054](http://dx.doi.org/10.1016/j.jpeds.2020.07.054)
411. Appiah-Kubi A, Acharya S, Fein Levy C, et al. Varying presentations and favourable outcomes of COVID-19 infection in children and young adults with sickle cell disease: an additional case series with comparisons to published cases. British Journal of Haematology 2020. doi: [10.1111/bjh.17013](http://dx.doi.org/10.1111/bjh.17013)
412. Lewis D, Fisler G, Schneider J, et al. Veno-venous extracorporeal membrane oxygenation for COVID-19-associated pediatric acute respiratory distress syndrome. Perfusion (United Kingdom) 2020. doi: [10.1177/0267659120939757](http://dx.doi.org/10.1177/0267659120939757)
413. Heinz N, Griesemer A, Kinney J, et al. A case of an Infant with SARS-CoV-2 hepatitis early after liver transplantation. Pediatric Transplantation 2020. doi: [10.1111/petr.13778](http://dx.doi.org/10.1111/petr.13778)
414. Krishnan US, Krishnan SS, Jain S, et al. SARS-CoV-2 Infection in Patients with Down Syndrome, Congenital Heart Disease, and Pulmonary Hypertension: Is Down Syndrome a Risk Factor? The Journal of pediatrics 2020. doi: [10.1016/j.jpeds.2020.06.076](http://dx.doi.org/10.1016/j.jpeds.2020.06.076)
415. Kihira S, Morgenstern PF, Raynes H, Naidich TP, Belani P. Fatal cerebral infarct in a child with COVID-19. Pediatric Radiology 2020. doi: [10.1007/s00247-020-04779-x](http://dx.doi.org/10.1007/s00247-020-04779-x)
416. Chao JY, Derespina KR, Herold BC, et al. Characteristics and Outcomes of Hospitalized and Critically Ill Children and Adolescents with Coronavirus Disease 2019 at a Tertiary Care Medical Center in New York City. Journal of Pediatrics 2020; 223(14).
417. Almassi N, Mulhall JP, Funt SA, Sheinfeld J. Case of the Month from Memorial Sloan Kettering Cancer Center, New York: Managing newly-diagnosed metastatic testicular germ cell tumor in a COVID-19 positive patient. BJU International 2020. doi: [10.1111/bju.15157](http://dx.doi.org/10.1111/bju.15157)
418. Gampel B, Troullioud Lucas AG, Broglie L et al. COVID-19 disease in New York City pediatric hematology and oncology patients. Pediatric blood & cancer 2020. doi: [10.1002/pbc.28420](https://dx.doi.org/10.1002/pbc.28420)
419. Choi NH, Silver ES, Fremed M, Liberman L. COVID-19 Reveals Brugada Pattern in an Adolescent Patient. Cardiology in the young 2020. doi: [10.1017/S1047951120002619](https://dx.doi.org/10.1017/S1047951120002619)
420. Trogen B, Gonzalez FJ, Shust GF. COVID-19-Associated Myocarditis in an Adolescent. Pediatric Infectious Disease Journal 2020. doi: [10.1097/INF.0000000000002788](http://dx.doi.org/10.1097/INF.0000000000002788)
421. Khoury R, Bernstein PS, Debolt C et al. Characteristics and Outcomes of 241 Births to Women With Severe Acute Respiratory Syndrome Coronavirus 2 (SARS-CoV-2) Infection at Five New York City Medical Centers. Obstetrics and gynecology 2020; 136(2): 273-282.
422. McLaren SH, Dayan PS, Fenster DB, et al. Novel Coronavirus Infection in Febrile Infants Aged 60 Days and Younger. Pediatrics 2020. doi: [10.1542/peds.2020-1550](https://dx.doi.org/10.1542/peds.2020-1550)
423. Salik I, Mehta B. Tetralogy of Fallot palliation in a COVID-19 positive neonate. Journal of Clinical Anesthesia 2020. doi: [10.1016/j.jclinane2020.109914](http://dx.doi.org/10.1016/j.jclinane.2020.109914)
424. McAbee GN, Brosgol Y, Pavlakis S, Agha R, Gaffoor M. Encephalitis Associated with COVID-19 Infection in an 11 Year-Old Child. Pediatric Neurology 2020. doi: 10.1016/j.pediatrneurol.2020.04.013
425. Farley M, Zuberi J. COVID-19 Precipitating Status Epilepticus in a Pediatric Patient. The American journal of case reports 2020; 21: e925776.
426. See Tsao H, HM Chason, DM Fearon. Immune Thrombocytopenia (ITP) in a SARS-CoV-2 Positive Pediatric Patient. Pediatrics 2020. doi: [10.1542/peds.2020-1419](http://dx.doi.org/10.1542/peds.2020-1419)
427. Otto WR, Geoghegan S, Posch LC, et al. The Epidemiology of SARS-CoV-2 in a Pediatric Healthcare Network in the United States. Journal of the Pediatric Infectious Diseases Society 2020. doi: [10.1093/jpids/piaa074](http://dx.doi.org/10.1093/jpids/piaa074)
428. Kan MJ, Grant LMC, Muna, MA, Greenhow TL. Fever without a source in a young infant due to SARS-CoV-2. Journal of the Pediatric Infectious Diseases Society 2020. doi: [10.1093/jpids/piaa044](https://dx.doi.org/10.1093/jpids/piaa044)
429. Robbins E, Ilahi Z, Roth P. Febrile Infant: COVID-19 in Addition to the Usual Suspects. The Pediatric infectious disease journal 2020. doi: [10.1097/INF.0000000000002693](https://dx.doi.org/10.1097/INF.0000000000002693)
430. Russell MR, Halnon NJ, Alejos JC, Salem MM, Reardon LC. COVID-19 in a pediatric heart transplant recipient: Emergence of donor-specific antibodies. Journal of Heart and Lung Transplantation 2020; 39(7): 732-733. doi: 10.1016/j.healun.2020.04.021
431. Bixler D, Miller AD, Mattison CP, et al. SARS-CoV-2-Associated Deaths Among Persons Aged <21 Years - United States, February 12-July 31, 2020. MMWR. Morbidity and mortality weekly report 2020; 69(37): 1324-1329
432. Woodworth KR, Olsen EO'M, Neelam V, et al. Birth and Infant Outcomes Following Laboratory-Confirmed SARS-CoV-2 Infection in Pregnancy - SET-NET, 16 Jurisdictions, March 29-October 14, 2020. MMWR. Morbidity and mortality weekly report 2020; 69(44): 1635-1640
433. Turbin RE, Wawrzusin PJ, Sakla NM, et al. Orbital cellulitis, sinusitis and intracranial abnormalities in two adolescents with COVID-19. Orbit (London) 2020. doi: [10.1080/01676830.2020.1768560](http://dx.doi.org/10.1080/01676830.2020.1768560)
434. Alloway BC, Yaeger SK, Mazzaccaro RJ, Villalobos T, Hardy SG. Suspected case of COVID-19-associated pancreatitis in a child. Radiology Case Reports. 2020; 15(8): 1309-1312.
435. Sisman J, Jaleel MA, Moreno W, et al. INTRAUTERINE TRANSMISSION OF SARS-COV-2 INFECTION IN A PRETERM INFANT. The Pediatric infectious disease journal 2020. doi: [10.1097/INF.0000000000002815](http://dx.doi.org/10.1097/INF.0000000000002815)
436. Stokes EK, Zambrano LD, Anderson KN, et al. Coronavirus Disease 2019 Case Surveillance - United States, January 22-May 30, 2020. MMWR. Morbidity and mortality weekly report 2020; 69(24): 759-765.
437. Team Cdc Covid- Response. Coronavirus Disease 2019 in Children - United States, February 12-April 2, 2020. MMWR. Morbidity and mortality weekly report 2020; 69(14): 422-426.
438. Shekerdemian LS, Mahmood NR, Wolfe KK, et al. Characteristics and Outcomes of Children with Coronavirus Disease 2019 (COVID-19) Infection Admitted to US and Canadian Pediatric Intensive Care Units. JAMA Pediatrics 2020. doi: [10.1001/jamapediatrics.2020.1948](http://dx.doi.org/10.1001/jamapediatrics.2020.1948)
439. Sachdeva R, Rice TB, Reisner B, et al. The Impact of Coronavirus Disease 2019 Pandemic on U.S. and Canadian PICUs. Pediatric critical care medicine: a journal of the Society of Critical Care Medicine and the World Federation of Pediatric Intensive and Critical Care Societies 2020. doi: [10.1097/PCC.0000000000002510](http://dx.doi.org/10.1097/PCC.0000000000002510)
440. Kim K, Choi JW, Moon J, et al. Clinical Features of COVID-19 in Uzbekistan. Journal of Korean medical science 2020. doi: 10.3346/jkms.2020.35.e404
441. Le HT, Nguyen LV, Tran, DM, et al. The first infant case of COVID-19 acquired from a secondary transmission in Vietnam. Lancet Child Adolesc Health 2020; 4(5): 405-406.
442. Nguyen TT, Pham TN, Van TD, et al. Genetic diversity of SARS-CoV-2 and clinical, epidemiological characteristics of COVID-19 patients in Hanoi, Vietnam. PloS one 2020. doi: 10.1371/journal.pone.0242537
443. Al-Waleedi AA, Naiene JD, Thabet AAK, et al. The first 2 months of the SARS-CoV-2 epidemic in Yemen: Analysis of the surveillance data. PloS one 2020. doi: 10.1371/journal.pone.0241260
