# Supplementary material for: The differential impact of pediatric COVID-19 between high-income countries and low- and middle-income countries: A systematic review of fatality and ICU admission in children worldwide
Source: PLoS One. 2021 Jan 29;16(1):e0246326. doi: 10.1371/journal.pone.0246326 (PMC7845974; doi:10.1371/journal.pone.0246326)
Supplement: S2 Table — (DOCX) [file pone.0246326.s007.docx]

**S2 Table. National fatality and ICU admission data in children aged 0-19 years with confirmed SARS-CoV-2 infection (Dec 7-11, 2020)**

|  | Income category | Date of Document update | Number of cases | Number of deaths | Number of ICU admissions | Deaths /1,000,000 children | Case Fatality rate | ICU admissions /1,000,000 children | ICU admission rate |
| --- | --- | --- | --- | --- | --- | --- | --- | --- | --- |
| Afghanistan ^1^ | L | 7-Dec | 2899 | 13 | N/A | 0.62 | 0.45% | N/A | N/A |
| Albania ^2,a^ | UM | 6-Dec | 2580 | 0 | N/A | 0.00 | 0.00% | N/A | N/A |
| Algeria ^3,b^ | UM | 6-Dec | 776 | 2 | N/A | 0.15 | 0.26% | N/A | N/A |
| American Samoa ^4^ | UM | 7-Dec | N/A | 0 | N/A | 0.00 | N/A | N/A | N/A |
| Andorra | H | 6-Dec | N/A | N/A | N/A | N/A | N/A | N/A | N/A |
| Angola | LM | 7-Dec | N/A | N/A | N/A | N/A | N/A | N/A | N/A |
| Antigua Barbuda ^5^ | H | 6-Dec | N/A | 0 | N/A | 0.00 | N/A | N/A | N/A |
| Argentina ^6^ | UM | 6-Dec | 124406 | 57 | N/A | 3.91 | 0.05% | N/A | N/A |
| Armenia | UM | 7-Dec | N/A | N/A | N/A | N/A | N/A | N/A | N/A |
| Aruba | H | 7-Dec | N/A | N/A | N/A | N/A | N/A | N/A | N/A |
| Australia ^7^ | H | 6-Dec | 3806 | 0 | 7 | 0.00 | 0.00% | 1.09 | 0.18% |
| Austria ^8,b^ | H | 9-Dec | 19682 | 3 | N/A | 2.31 | 0.02% | N/A | N/A |
| Azerbaijan ^c^ | UM | 6-Dec | 16135 | N/A | N/A | N/A | N/A | N/A | N/A |
| Bahamas | H | 4-Dec | N/A | N/A | N/A | N/A | N/A | N/A | N/A |
| Bahrain | H | 6-Dec | N/A | N/A | N/A | N/A | N/A | N/A | N/A |
| Bangladesh ^9,a^ | LM | 6-Dec | 48710 | 158 | N/A | 2.52 | 0.32% | N/A | N/A |
| Barbados ^10^ | H | 5-Dec | N/A | 0 | N/A | 0.00 | N/A | N/A | N/A |
| Belarus | UM | 6-Dec | N/A | N/A | N/A | N/A | N/A | N/A | N/A |
| Belgium ^11,d^ | H | 6-Dec | 71142 | 6 | N/A | 1.83 | 0.01% | N/A | N/A |
| Belize | UM | 5-Dec | N/A | N/A | N/A | N/A | N/A | N/A | N/A |
| Benin | L | 30-Nov | 434 | N/A | N/A | N/A | N/A | N/A | N/A |
| Bermuda ^12^ | H | 5-Dec | 16 | 0 | N/A | 0.00 | 0.00% | N/A | N/A |
| Bhutan ^13^ | LM | 6-Dec | N/A | 0 | N/A | 0.00 | N/A | N/A | N/A |
| Bolivia ^14,e^ | LM | 6-Dec | (1047) | (4) | N/A | N/A | N/A | N/A | N/A |
| Bosnia Herzegovina ^15^ | UM | 7-Dec | 4696 | 0 | N/A | 0.00 | 0.00% | N/A | N/A |
| Botswana | UM | 7-Dec | N/A | N/A | N/A | N/A | N/A | N/A | N/A |
| Brazil ^16^ | UM | 10-Dec | N/A | 1419 | N/A | 23.56 | N/A | N/A | N/A |
| British Virgin Islands ^17^ | H | 7-Dec | N/A | 0 | N/A | 0.00 | N/A | N/A | N/A |
| Brunei ^f^ | H | 15-Jun | 23 | 0 | 0 | 0.00 | 0.00% | 0.00 | 0.00% |
| Bulgaria | UM | 7-Dec | 23007 | N/A | N/A | N/A | N/A | N/A | N/A |
| Burkina Faso | L | 11-Dec | N/A | N/A | N/A | N/A | N/A | N/A | N/A |
| Burundi ^18^ | L | 11-Dec | N/A | 0 | N/A | 0.00 | N/A | N/A | N/A |
| Cabo Verde ^c^ | LM | 25-Nov | 2187 | N/A | N/A | N/A | N/A | N/A | N/A |
| Cambodia ^19^ | LM | 30-Nov | 9 | 0 | N/A | 0.00 | 0.00% | N/A | N/A |
| Cameroon | LM | 6-Dec | N/A | N/A | N/A | N/A | N/A | N/A | N/A |
| Canada ^20^ | H | 6-Dec | 57559 | 3 | 51 | 0.38 | 0.01% | 6.42 | 0.09% |
| Cayman Islands | H | 7-Dec | N/A | N/A | N/A | N/A | N/A | N/A | N/A |
| Central African Republic | L | 20-Oct | 379 | N/A | N/A | N/A | N/A | N/A | N/A |
| Chad | L | 21-Oct | 18 | N/A | N/A | N/A | N/A | N/A | N/A |
| Channel Islands ^21^ | H | 7-Dec | N/A | 0 | N/A | 0.00 | N/A | N/A | N/A |
| Chile ^22^ | H | 11-Nov | 57375 | 48 | N/A | 9.75 | 0.08% | N/A | N/A |
| China ^f^ | UM |  | 793 | 2 | 21 | 0.01 | 0.25% | 0.06 | 2.65% |
| Colombia ^23^ | UM | 6-Dec | 132052 | 121 | 96 | 7.79 | 0.09% | 6.18 | 0.09% |
| Comoros | LM | 10-Dec | N/A | N/A | N/A | N/A | N/A | N/A | N/A |
| Congo ^24^ | LM | 16-Nov | 268 | 2 | N/A | 0.70 | 0.75% | N/A | N/A |
| Costa Rica ^25^ | UM | 7-Dec | 12082 | 2 | N/A | 1.40 | 0.02% | N/A | N/A |
| Cote D'Ivoire | LM | 7-Dec | N/A | N/A | N/A | N/A | N/A | N/A | N/A |
| Croatia ^26^ | H | 7-Dec | 18492 | 1 | N/A | 1.19 | 0.01% | N/A | N/A |
| Cuba ^27^ | UM | 7-Dec | 1115 | 0 | N/A | 0.00 | 0.00% | N/A | N/A |
| Curaçao | H | 7-Dec | N/A | N/A | N/A | N/A | N/A | N/A | N/A |
| Cyprus | H | 11-Dec | N/A | N/A | N/A | N/A | N/A | N/A | N/A |
| Czech Republic ^28,b^ | H | 8-Dec | 45851 | 0 | N/A | 0.00 | 0.00% | N/A | N/A |
| Denmark ^29^ | H | 8-Dec | 21246 | 0 | N/A | 0.00 | 0.00% | N/A | N/A |
| Diamond princess ^30^ | N/A | 20-Feb | 6 | 0 | N/A | N/A | 0.00% | N/A | N/A |
| Djibouti | LM | 7-Dec | N/A | N/A | N/A | N/A | N/A | N/A | N/A |
| Dominica ^31^ | UM | 7-Dec | N/A | 0 | N/A | 0.00 | N/A | N/A | N/A |
| Dominican Republic ^32^ | UM | 30-Oct | 11864 | 31 | N/A | 7.88 | 0.26% | N/A | N/A |
| DRC | L | 1-Dec | N/A | N/A | N/A | N/A | N/A | N/A | N/A |
| Ecuador ^33^ | UM | 9-Dec | 10801 | 63 | N/A | 9.85 | 0.58% | N/A | N/A |
| Egypt | LM | 7-Dec | N/A | N/A | N/A | N/A | N/A | N/A | N/A |
| El Salvador ^34,g^ | LM | 10-Dec | 1740 | 7 | N/A | 3.03 | 0.40% | N/A | N/A |
| Equatorial Guinea | UM | 7-Dec | N/A | N/A | N/A | N/A | N/A | N/A | N/A |
| Eritrea ^35^ | L | 7-Dec | N/A | 0 | N/A | 0.00 | N/A | N/A | N/A |
| Estonia ^36,37^ | H | 9-Dec | 2316 | 0 | N/A | 0.00 | 0.00% | N/A | N/A |
| Eswatini ^38^ | LM | 8-Dec | 928 | 2 | N/A | 3.55 | 0.22% | N/A | N/A |
| Ethiopia | L | 7-Aug | 731 | N/A | N/A | N/A | N/A | N/A | N/A |
| Faroe islands ^39^ | H | 9-Dec | 51 | 0 | N/A | 0.00 | 0.00% | N/A | N/A |
| Fiji ^40^ | UM | 8-Dec | 3 | 0 | N/A | 0.00 | 0.00% | N/A | N/A |
| Finland ^41^ | H | 9-Dec | 4835 | 0 | N/A | 0.00 | 0.00% | N/A | N/A |
| France ^42^ | H | 14-Dec | N/A | 9 | N/A | 0.58 | N/A | N/A | N/A |
| French Polynesia | H | 7-Dec | N/A | N/A | N/A | N/A | N/A | N/A | N/A |
| Gabon | UM | 26-Aug | N/A | N/A | N/A | N/A | N/A | N/A | N/A |
| Gambia | L | 8-Dec | 333 | N/A | N/A | N/A | N/A | N/A | N/A |
| Georgia | UM | 9-Dec | N/A | N/A | N/A | N/A | N/A | N/A | N/A |
| Germany ^43^ | H | 10-Dec | 100013 | 10 | N/A | 0.63 | 0.01% | N/A | N/A |
| Ghana ^44^ | LM | 30-Nov | 2180 | 4 | N/A | 0.27 | 0.18% | N/A | N/A |
| Gibraltar ^45^ | H | 7-Dec | N/A | 0 | N/A | 0.00 | N/A | N/A | N/A |
| Greece ^46,h^ | H | 4-Dec | 6938 | 0 | N/A. | 0.00 | 0.00% | N/A | N/A |
| Greenland ^47^ | H | 7-Dec | N/A | 0 | N/A. | 0.00 | N/A | N/A | N/A |
| Grenada ^48^ | UM | 7-Dec | N/A | 0 | N/A | 0.00 | N/A | N/A | N/A |
| Guam ^49^ | H | 3-Dec | 1086 | 1 | N/A | 18.52 | 0.09% | N/A | N/A |
| Guatemala ^50^ | UM | 8-Dec | 5040 | 50 | N/A | 6.31 | 0.99% | N/A | N/A |
| Guinea | L | 9-Dec | N/A | N/A | N/A | N/A | N/A | N/A | N/A |
| Guinea-Bissau | L | 6-Nov | 302 | N/A | N/A | N/A | N/A | N/A | N/A |
| Guyana | UM | 10-Dec | N/A | N/A | N/A | N/A | N/A | N/A | N/A |
| Haiti ^51^ | L | 7-Dec | 632 | 12 | N/A. | 2.48 | 1.90% | N/A | N/A |
| Honduras ^52^ | LM | 6-Dec | 9733 | 38 | N/A | 9.33 | 0.39% | N/A | N/A |
| Hong Kong ^53^ | H | 7-Dec | 677 | 0 | N/A | 0.00 | 0.00% | N/A | N/A |
| Hungary ^54,55^ | H | 10-Dec | 11207 | 1 | N/A | 0.53 | 0.01% | N/A | N/A |
| Iceland ^56,h^ | H | 10-Dec | 492 | 0 | 0 | 0.00 | 0.00% | N/A | N/A |
| India | LM | 10-Dec | N/A | N/A | N/A. | N/A | N/A | N/A | N/A |
| Indonesia ^57,a,i^ | LM | 10-Dec | 68877 | 532 | N/A | 5.64 | 0.77% | N/A | N/A |
| Iran | UM | 10-Dec | N/A | N/A | N/A. | N/A | N/A | N/A | N/A |
| Iraq | UM | 7-Dec | N/A | N/A | N/A | N/A | N/A | N/A | N/A |
| Ireland ^58,b^ | H | 7-Dec | 6848 | 0 | 6 | 0.00 | 0.00% | 5.83 | 0.09% |
| Isle of Man | H | 3-Dec | 23 | N/A | N/A | N/A | N/A | N/A | N/A |
| Israel ^59,a^ | H | 15-Nov | 101567 | 4 | N/A | 1.30 | 0.00% | N/A | N/A |
| Italy ^60^ | H | 2-Dec | 192735 | 12 | N/A | 1.12 | 0.01% | N/A | N/A |
| Jamaica ^61^ | UM | 10-Dec | 1232 | 3 | N/A | 3.22 | 0.24% | N/A | N/A |
| Japan ^62^ | H | 9-Dec | 12629 | 0 | N/A | 0.00 | 0.00% | N/A | N/A |
| Jordan | UM | 7-Dec | N/A | N/A | N/A | N/A | N/A | N/A | N/A |
| Kazakhstan | UM | 7-Dec | N/A | N/A | N/A | N/A | N/A | N/A | N/A |
| Kenya | LM | 27-Jul | N/A | N/A | N/A | N/A | N/A | N/A | N/A |
| Kiribati ^63^ | LM | 10-Dec | 0 | 0 | 0 | 0.00 | No case | 0.00 | No case |
| Kosovo | UM | 10-Dec | N/A | N/A | N/A | N/A | N/A | N/A | N/A |
| Kuwait | H | 7-Dec | N/A | N/A | N/A | N/A | N/A | N/A | N/A |
| Kyrgyzstan | LM | 10-Dec | 227 | N/A | N/A | N/A | N/A | N/A | N/A |
| Laos ^64^ | LM | 25-Nov | 1 | 0 | 0 | 0.00 | 0.00% | 0.00 | 0.00% |
| Latvia ^65^ | H | 10-Dec | 2438 | 0 | N/A | 0.00 | 0.00% | N/A | N/A |
| Lebanon ^66^ | UM | 9-Dec | 10553 | 3 | N/A | 1.31 | 0.03% | N/A | N/A |
| Lesotho | LM | 10-Dec | N/A | N/A | N/A | N/A | N/A | N/A | N/A |
| Liberia | L | 10-Dec | N/A | N/A | N/A | N/A | N/A | N/A | N/A |
| Libya | UM | 7-Dec | N/A | N/A | N/A | N/A | N/A | N/A | N/A |
| Lichtenstein | H | 3-Dec | N/A | N/A | N/A | N/A | N/A | N/A | N/A |
| Lithuania | H | 3-Dec | 2833 | N/A | N/A | N/A | N/A | N/A | N/A |
| Luxembourg ^55,67^ | H | 9-Dec | 2269 | 0 | N/A | 0.00 | 0.00% | N/A | N/A |
| Macao ^68^ | H | 9-Dec | 9 | 0 | N/A | 0.00 | 0.00% | N/A | N/A |
| Madagascar | L | 9-Dec | N/A | N/A | N/A | N/A | N/A | N/A | N/A |
| Malawi ^69^ | L | 9-Dec | N/A | 1 | N/A | 0.10 | N/A | N/A | N/A |
| Malaysia ^70^ | UM | 9-Nov | N/A | 2 | N/A | 0.19 | N/A | N/A | N/A |
| Maldives ^71^ | UM | 9-Dec | 2067 | 0 | N/A | 0.00 | 0.00% | N/A | N/A |
| Mali | L | 29-Nov | N/A | N/A | N/A | N/A | N/A | N/A | N/A |
| Malta ^72^ | H | 7-Dec | N/A | 0 | N/A | 0.00 | N/A | N/A | N/A |
| Marshal Islands ^73^ | UM | 9-Dec | 0 | 0 | 0 | 0.00 | No case | 0.00 | No case |
| Mauritania | LM | 7-Dec | N/A | N/A | N/A | N/A | N/A | N/A | N/A |
| Mauritius ^74^ | UM | 7-Dec | N/A | 0 | N/A | 0.00 | N/A | N/A | N/A |
| Mexico ^75^ | UM | 7-Dec | 20450 | 242 | 428 | 5.44 | 1.18% | 9.61 | 2.09% |
| Micronesia ^76^ | LM | 9-Dec | 0 | 0 | 0 | 0.00 | No case | 0.00 | No case |
| Moldova ^77^ | LM | 9-Dec | 8389 | 2 | NA | 0.27 | 0.02% | N/A | N/A |
| Monaco ^78^ | H | 9-Dec | N/A | 0 | N/A | 0.00 | N/A | N/A | N/A |
| Mongolia ^79^ | LM | 9-Dec | 185 | 0 | N/A | 0.00 | 0.00% | N/A | N/A |
| Montenegro | UM | 9-Dec | 3882 | N/A | N/A | N/A | N/A | N/A | N/A |
| Morocco | LM | 7-Dec | N/A | N/A | N/A | N/A | N/A | N/A | N/A |
| Mozambique ^80,a^ | L | 9-Dec | 1416 | 7 | N/A | 0.40 | 0.49% | N/A | N/A |
| Myanmar | LM | 6-Dec | N/A | N/A | N/A | N/A | N/A | N/A | N/A |
| Namibia ^81,82^ | UM | 22-Nov | 2113 | 3 | N/A | 2.54 | 0.14% | N/A | N/A |
| Nauru ^63^ | UM | 9-Dec | 0 | 0 | 0 | 0.00 | No case | 0.00 | No case |
| Nepal ^83^ | L | 2-Dec | 10595 | 11 | N/A | 0.95 | 0.10% | N/A | N/A |
| Netherlands ^84^ | H | 8-Dec | 72791 | 2 | NA | 0.54 | 0.00% | N/A | N/A |
| New Caledonia ^f^ | H |  | 1 | 0 | 0 | 0.00 | 0.00% | 0.00 | 0.00% |
| New Zealand ^85^ | H | 9-Dec | 274 | 0 | N/A | 0.00 | 0.00% | N/A | N/A |
| Nicaragua ^86^ | LM | 9-Dec | 320 | 12 | N/A | 4.69 | 3.75% | N/A | N/A |
| Niger | L | 8-Dec | N/A | N/A | N/A | N/A | N/A | N/A | N/A |
| Nigeria ^87^ | LM | 5-Dec | 7656 | 30 | N/A | 0.26 | 0.39% | N/A | N/A |
| North Korea | L | 8-Dec | N/A | N/A | N/A | N/A | N/A | N/A | N/A |
| North Macedonia ^88,a^ | UM | 8-Dec | 4702 | 1 | N/A | 2.17 | 0.02% | N/A | N/A |
| Northen Mariana Islands ^89^ | H | 7-Dec | 26 | 0 | N/A | 0.00 | 0.00% | N/A | N/A |
| Norway | H | 8-Dec | 6639 | N/A | N/A | N/A | N/A | N/A | N/A |
| Oman | H | 7-Dec | N/A | N/A | N/A | N/A | N/A | N/A | N/A |
| Pakistan | LM | 27-Jun | N/A | N/A | N/A | N/A | N/A | N/A | N/A |
| Palau ^90^ | H | 8-Dec | 0 | 0 | 0 | 0.00 | No case | 0.00 | No case |
| Palestine | LM | 8-Dec | 16574 | N/A | N/A | N/A | N/A | N/A | N/A |
| Panama ^91^ | H | 7-Dec | 31352 | 24 | N/A | 16.00 | 0.08% | N/A | N/A |
| Papua New Guinea ^92^ | LM | 7-Dec | N/A | 0 | N/A | 0.00 | N/A | N/A | N/A |
| Paraguay ^93^ | UM | 7-Dec | 6445 | 5 | N/A | 1.84 | 0.08% | N/A | N/A |
| Peru ^94^ | UM | 7-Dec | N/A | 221 | N/A | 20.89 | N/A | N/A | N/A |
| Philippine ^95^ | LM | 8-Dec | 36823 | 167 | N/A | 3.85 | 0.45% | N/A | N/A |
| Poland ^55,96^ | H | 9-Oct | 3349 | 2 | N/A | 0.27 | 0.06% | N/A | N/A |
| Portugal ^97^ | H | 8-Dec | 45485 | 0 | N/A | 0.00 | 0.00% | N/A | N/A |
| Puerto Rico | H | 7-Dec | 7750 | N/A | N/A | N/A | N/A | N/A | N/A |
| Qatar | H | 7-Dec | N/A | N/A | N/A | N/A | N/A | N/A | N/A |
| Romania ^98,99^ | UM | 7-Dec | 28976 | 9 | N/A | 2.26 | 0.03% | N/A | N/A |
| Russia | UM | 22-Jun | N/A | N/A | N/A | N/A | N/A | N/A | N/A |
| Rwanda ^100,101^ | L | 8-Dec | 771 | 0 | N/A | 0.00 | 0.00% | N/A | N/A |
| Saint Kitts and Nevis ^102^ | H | 7-Dec | N/A | 0 | N/A | 0.00 | N/A | N/A | N/A |
| Saint Lucia ^103^ | UM | 7-Dec | N/A | 0 | N/A | 0.00 | N/A | N/A | N/A |
| Saint Maarten | H | 6-Dec | N/A | N/A | N/A | N/A | N/A | N/A | N/A |
| Saint Martin | H | 8-Dec | N/A | N/A | N/A | N/A | N/A | N/A | N/A |
| Saint Vincent and the Grenadine ^104^ | UM | 7-Dec | N/A | 0 | N/A | 0.00 | N/A | N/A | N/A |
| Samoa ^105^ | UM | 7-Dec | 0 | 0 | 0 | 0.00 | No case | 0.00 | No case |
| San Marino | H | 8-Dec | N/A | N/A | N/A | N/A | N/A | N/A | N/A |
| Sao Tome and Principe ^106^ | LM | 4-Nov | 108 | 1 | N/A | 8.55 | 0.93% | N/A | N/A |
| Saudi Arabia | H | 7-Dec | N/A | N/A | N/A | N/A | N/A | N/A | N/A |
| Senegal | LM | 7-Dec | 99 | N/A | N/A | N/A | N/A | N/A | N/A |
| Serbia | UM | 8-Dec | N/A | N/A | N/A | N/A | N/A | N/A | N/A |
| Seychelles ^107^ | H | 7-Dec | N/A | 0 | N/A | 0.00 | N/A | N/A | N/A |
| Sierra Leone | L | 8-Dec | N/A | N/A | N/A | N/A | N/A | N/A | N/A |
| Singapore ^108^ | H | 7-Dec | 133 | 0 | N/A | 0.00 | 0.00% | N/A | N/A |
| Slovakia | H | 11-Dec | N/A | N/A | N/A | N/A | N/A | N/A | N/A |
| Slovenia ^109,b^ | H | 11-Dec | 2094 | 0 | N/A | 0.00 | 0.00% | N/A | N/A |
| Solomon Islands ^110^ | LM | 11-Dec | N/A | 0 | 0 | 0.00 | N/A | N/A | N/A |
| Somalia ^111^ | L | 11-Dec | 215 | 0 | N/A | 0.00 | 0.00% | N/A | N/A |
| South Africa ^112^ | UM | 4-Dec | 75529 | 114 | 79 | 5.18 | 0.15% | 3.59 | 0.24% |
| South Korea ^113^ | H | 7-Dec | 3482 | 0 | N/A | 0.00 | 0.00% | N/A | N/A |
| South Sudan | L | 7-Dec | N/A | N/A | N/A | N/A | N/A | N/A | N/A |
| Spain ^114,b^ | H | 7-Dec | 157858 | 13 | 89 | 1.93 | 0.01% | 13.22 | 0.40% |
| Sri Lanka | UM | 7-Dec | N/A | N/A | N/A | N/A | N/A | N/A | N/A |
| Sudan | LM | 7-Dec | N/A | N/A | N/A | N/A | N/A | N/A | N/A |
| Suriname | UM | 7-Dec | N/A | N/A | N/A | N/A | N/A | N/A | N/A |
| Sweden ^115^ | H | 7-Dec | 29113 | 3 | 37 | 1.29 | 0.01% | 15.94 | 0.13% |
| Switzerland ^116^ | H | 1-Dec | 29801 | 1 | N/A | 0.58 | 0.00% | N/A | N/A |
| Syria | L | 7-Dec | N/A | N/A | N/A | N/A | N/A | N/A | N/A |
| Taiwan ^117^ | H | 7-Dec | 25 | 0 | N/A | 0.00 | 0.00% | N/A | N/A |
| Tajikistan | L | 7-Dec | N/A | N/A | N/A | N/A | N/A | N/A | N/A |
| Tanzania | L | 7-Dec | N/A | N/A | N/A | N/A | N/A | N/A | N/A |
| Thailand ^118^ | UM | 7-Dec | 248 | 0 | N/A | 0.00 | 0.00% | N/A | N/A |
| Timor-Leste ^119^ | LM | 7-Dec | N/A | 0 | N/A | 0.00 | N/A | N/A | N/A |
| Togo ^120,b^ | L | 7-Dec | 237 | 4 | N/A | 1.19 | 1.69% | N/A | N/A |
| Tonga ^63^ | UM | 7-Dec | 0 | 0 | 0 | 0.00 | No case | 0.00 | No case |
| Trinidad Tobago | H | 14-Sep | N/A | N/A | N/A | N/A | N/A | N/A | N/A |
| Tunisia | LM | 7-Dec | N/A | N/A | N/A | N/A | N/A | N/A | N/A |
| Turk and Caicos Islands | H | 7-Dec | 117 | N/A | N/A | N/A | N/A | N/A | N/A |
| Turkey ^121.a^ | UM | 25-Oct | 22333 | 16 | N/A | 0.59 | 0.07% | N/A | N/A |
| Turkmenistan | UM | 7-Dec | N/A | N/A | N/A | N/A | N/A | N/A | N/A |
| Tuvalu ^63^ | UM | 7-Dec | 0 | 0 | 0 | 0.00 | No case | 0.00 | No case |
| UAE | H | 7-Dec | N/A | N/A | N/A | N/A | N/A | N/A | N/A |
| Uganda ^122^ | L | 1-Dec | 420 | 0 | N/A | 0.00 | 0.00% | N/A | N/A |
| UK ^123-27,j^ | H | 7-Dec | 230373 | 22 | N/A | 1.40 | 0.01% | N/A | N/A |
| Ukraine ^128^ | LM | 16-Nov | 39223 | 18 | N/A | 0.20 | 0.05% | N/A | N/A |
| Uruguay ^129,b^ | H | 30-Nov | 565 | 0 | 0 | 0.00 | 0.00% | 0.00 | 0.00% |
| US Virgin islands ^130,g^ | H | 5-Dec | 115 | 0 | N/A | 0.00 | 0.00% | N/A | N/A |
| USA ^131,32,k^ | H | 3-Dec | 1305305 | 154 | 1830 | 1.88 | 0.01% | 22.30 | 0.13% |
| Uzbekistan | LM | 7-Dec | N/A | N/A | N/A | N/A | N/A | N/A | N/A |
| Vanuatu ^133^ | LM | 1-Dec | 0 | 0 | 0 | 0.00 | No case | 0.00 | No case |
| Venezuela ^134,g^ | UM | 7-Dec | 1585 | 1 | N/A | 0.10 | 0.06% | N/A | N/A |
| Vietnam ^135^ | LM | 7-Dec | 110 | 0 | N/A | 0.00 | 0.00% | N/A | N/A |
| Yemen | L | 7-Dec | N/A | N/A | N/A | N/A | N/A | N/A | N/A |
| Zambia | LM | 7-Dec | N/A | N/A | N/A | N/A | N/A | N/A | N/A |
| Zimbabwe ^136^ | LM | 2-Dec | 719 | 6 | N/A | 0.76 | 0.83% | N/A | N/A |

Abbreviations: ICU, intensive care unit; H, high-income; UM, upper middle-income; LM, lower middle-income; L, low-income; N/A, not available

National reports published after December 11 may be used in some countries because of the temporary maintenance of the national websites in the data extraction period or the time required for the data clarifications.

Some countries report the cases diagnosed by antigen and/or antibody tests. The data need to be interpreted with caution.

National reports published more than 2 months before Dec 7 are not presented, but included in the sensitivity analysis (Supplemental Table 6)

^a^ The pediatric number was calculated from the total number and the proportion of pediatric population.

^b^ Pediatric age was defined as 0-14 years

^c^ Pediatric age was defined as 0-20 years

^d^ The number of deaths was reported as 0-24 years.

^e^ Only sub-national data were available. National outcomes were unable to be calculated.

^f^ The extracted data of national articles from database search were applied (Table S1).

^g^ The age information of deaths was not reported for some duration (up to 2 months)

^h^ Pediatric age was defined as 0-17 years

^i^ Pediatric age was defined as 0-18 years

^j^ UK: The date of national report from England was November 25 for confirmed cases and November 20 for deaths

^k^ USA: The number of confirmed cases in 43 states and New York City was used for the denominator of national case fatality rate because other states did not report pediatric deaths data as of December 3.

**References**

1. Ministry of Public Health. M&E - Health Information System General Directorate. Available at https://moph-dw.gov.af/dhis-web-dashboard/index.html#/. Accessed December 7, 2020.
2. National Agency for Information Society. Coronavirus Albania. Available at https://coronavirus.al/statistika/. Accessed December 7, 2020.
3. Ministère de la santé, de la population et de la réforme hospitalière. Carte épidémiologique. Available at https://corona-dz.live/. Accessed December 7, 2020.
4. WHO American Samoa. Pacific Island Information on COVID-19. Available at https://www.who.int/americansamoa. Accessed December 7, 2020.
5. Antigua and Barbuda’s Government Information and Services. Novel Coronavirus Information. Available at https://ab.gov.ag/detail_page.php?page=42. Accessed December 7, 2020.
6. Ministerio de salud de la nación. Sala de situación coronavirus online. Available at https://www.argentina.gob.ar/salud/coronavirus-COVID-19/sala-situacion. Accessed December 7, 2020.
7. Department of health. Coronavirus disease (COVID-19) epidemiology reports, Australia, 2020. Available at https://www1.health.gov.au/internet/main/publishing.nsf/Content/novel_coronavirus_2019_ncov_weekly_epidemiology_reports_australia_2020.htm. Accessed December 7, 2020.
8. Federal ministry republic of Austria social affairs, health, care and consumer protection. Corona virus current information. Available at https://www.sozialministerium.at/Informationen-zum-Coronavirus/Neuartiges-Coronavirus-(2019-nCov).html. Accessed December 9, 2020.
9. Institute of epidemiology, disease control and research. Bangladesh covid-19 update. Available at https://www.iedcr.gov.bd/. Accessed December 7, 2020.
10. Government Information Service. COVID-19 Update. Available at https://gisbarbados.gog.bb/covid-19/. Accessed December 7, 2020.
11. Epidémiologie des maladies infectieuses. Covid-19-situation épidémiologique. Available at https://epidemio.wiv-isp.be/ID/Pages/2019-nCoV_epidemiological_situation.aspx?lcid=1036. Accessed December 7, 2020.
12. Government of Bermuda. Update COVID-19. Available at https://www.gov.bm/coronavirus. Accessed December 7, 2020.
13. Ministry of Health, Royal Government of Bhutan, Thimphu, Bhutan. COVID-19 public notice. Available at https://www.gov.bm/coronavirus. Accessed December 7, 2020.
14. Municipal Autonomous Government La Paz. COVID-19 health emergency report. Available at http://observatoriocovid19.lapaz.bo/observatorio/index.php. Accessed December 7, 2020.
15. Federalno ministarstvo zdravstva. Koronavirus (covid-19) informativni portal. Available at https://covid19.fmoh.gov.ba/. Accessed December 7, 2020.
16. Ministério da saúde. Boletim epidemiológico especial. Available at https://www.saude.gov.br/boletins-epidemiologicos. Accessed December 10, 2020.
17. Government of the Virgin Islands. Coronavirus (COVID-19) Updates. Available at https://bvi.gov.vg/. Accessed December 7, 2020.
18. Government of the Republic of Burundi. Bureau du Primiere Ministre. Available at http://www.burundi.gov.bi/#. Accessed December 11 2020.
19. Ministry of Health and World Health Organization. Cambodia Coronavirus Disease 2019 (COVID-19) Situation Report. Available at http://www.cdcmoh.gov.kh/resource-documents/who-moh-situation-report. Accessed December 11, 2020.
20. Government of Canada. Coronavirus disease (covid-19): outbreak update. Coronavirus disease 2019 (covid-19) daily epidemiology update. Available at https://www.canada.ca/content/dam/phac-aspc/documents/services/diseases/2019-novel-coronavirus-infection/surv-covid19-epi-update-eng.pdf. Accessed December 7, 2020.
21. Government of Jersey. Coronavirus (COVID-19) tests and cases in Jersey. Available at https://www.gov.je/Health/Coronavirus/Pages/CoronavirusCases.aspx. Accessed December 7 2020.
22. Ministerio de salud. Informe epidemiológico. niños con covid-19. 2020 – Minsal, Available at https://www.minsal.cl/nuevo-coronavirus-2019-ncov/informe-epidemiologico-covid-19/. Accessed December 7 2020.
23. Sistema Integral de Información SISPRO - Ministerio de Salud. Situación de COVID-19 en Colombia. Available at https://sig.sispro.gov.co/SituacionCovid/. Accessed December 7, 2020.
24. Ministerio de salud. Réponse à l'épidémie de COVID-19 au Congo. Available at /http://sante.gouv.cg/wp-content/uploads/2020/11/SITREP-N-111-COVID-19-CONGO-16-11-2020-1.pdf. Accessed December 7, 2020.
25. Ministerio de salud. Situacion nacional covid-19. Available at https://www.ministeriodesalud.go.cr/index.php/centro-de-prensa/noticias/741-noticias-2020/1532-lineamientos-nacionales-para-la-vigilancia-de-la-infeccion-por-coronavirus-2019-ncov. Accessed December 7, 2020.
26. Koronavirus.hr. Podaci. Available at https://koronavirus.hr/podaci/489. Accessed December 7, 2020.
27. Ministerio de Salud Pública de Cuba. Covid19 Cuba data. Available at https://covid19cubadata.github.io/#cuba. Accessed December 8, 2020.
28. Ministry of health of the Czech Republic. Covid‑19: overview of the current situation in the Czech Republic. (Czech) Available at https://onemocneon-aktualne.mzcr.cz/covid-19. Accessed December 8, 2020.
29. Statens serum institute. Dagens covid-19 tal for Danmark. Available at https://experience.arcgis.com/experience/aa41b29149f24e20a4007a0c4e13db1d. Accessed December 8, 2020.
30. National institute of infectious diseases. Field briefing: diamond princess covid-19 cases, 20 Feb update. Available at https://www.niid.go.jp/niid/ja/diseases/ka/corona-virus/2019-ncov/2484-idsc/9422-covid-dp-2.html. Accessed December 8, 2020.
31. Ministry of Health, Wellness. New Health Investment Response to COVID-19. Available at http://dominica.gov.dm/corona. Accessed December 7, 2020.
32. Ministry of Public Health and Social Assistance. COVID-19 Newsletters. Available at https://coronavirusrd.gob.do/documentos/boletines/. Accessed December 11, 2020.
33. Ministerio de salud pública. COVID-19 MSP. Available at https://public.tableau.com/profile/direcci.n.nacional.de.vigilancia.epidemiol.gica.msp#!/vizhome/COVID19ecu_MSP_DNVE/COVID-19MSP. Accessed December 10, 2020.
34. Gobierno de El Salvador. Situación nacional covid-19. Available at https://covid19.gob.sv/. Accessed December 10, 2020.
35. Ministry of Information. COVID-19. Available at http://www.shabait.com/home. Accessed December 7, 2020.
36. Terviseamet. Coronavirus data. Available at https://www.terviseamet.ee/et/koroonaviirus/koroonakaart. Accessed December 9 2020.
37. Republic of Estonia Health board. Current situation in Estonia. Available at https://www.terviseamet.ee/en/covid19. Accessed December 9, 2020.
38. Government of the kingdom of Eswatini. National response to covid19 updates. Available at https://datastudio.google.com/embed/reporting/b847a713-0793-40ce-8196-e37d1cc9d720/page/2a0LB. Accessed December 9, 2020.
39. The Government of the Faroe Island. COVID-19 Faroe Islands – Statistics. Available at https://corona.fo/statistics?_l=en. Accessed December 9 2020.
40. Ministry of Health & Medical Services. COVID-19 UPDATES. Available at http://www.health.gov.fj/covid-19-updates/. Accessed Devember 9, 2020.
41. Finnish institute for health and welfare. Situation update on coronavirus. Available at https://thl.fi/en/web/infectious-diseases/what-s-new/coronavirus-covid-19-latest-updates/situation-update-on-coronavirus. Accessed December 9, 2020.
42. Sante publique France. Infection au nouveau coronavirus (SARS-CoV-2), covid-19. Available at https://www.santepubliquefrance.fr/maladies-et-traumatismes/maladies-et-infections-respiratoires/infection-a-coronavirus/articles/infection-au-nouveau-coronavirus-sars-cov-2-covid-19-france-et-monde#block-242818. Accessed December 15, 2020.
43. Robert Koch institute. Coronavirus disease 2019 (covid-19) daily situation report of the Robert Koch institute. Available at https://www.rki.de/DE/Content/InfAZ/N/Neuartiges_Coronavirus/Situationsberichte/Gesamt.html. Accessed December 10, 2020.
44. UNICEF. GHANA: COVID-19 Situation Report. Available at https://www.unicef.org/search?force=0&query=GHANA+SITUATION. . Accessed December 10, 2020.
45. Public Health Gibraltar. Update on COVID-19. Available at https://healthygibraltar.org/news/update-on-wuhan-coronavirus/. Accessed December 7, 2020.
46. National public health organization. Daily epidemiological surveillance report of novel coronavirus (covid-19). (Greek) Available at https://eody.gov.gr/. Accessed December 8, 2020.
47. Landslægeembedets. Antal i Grønland. (Danish) Available at https://nun.gl/. Accessed December 7, 2020.
48. Government Information Service of Grenada. Covid-19 Update statement. Available at https://www.facebook.com/GISgrenada/photos/pcb.3241264486093241/3241262572765780/?type=3&theater. Accessed December 7, 2020.
49. Guam Homeland Security Office of Civil Defense. Information on Coronavirus (COVID-19). Available at https://ghs.guam.gov/coronavirus-covid-19. Accessed December 8, 2020.
50. Ministerio de Salud Publica y Asistencia Social. Situación de COVID-19 en Guatemala. Available at https://tablerocovid.mspas.gob.gt/. Accessed　December 8, 2020.
51. La ministre de la santé publique et de la population. Rapport de surveillance sur le Covid-19. Available at https://mspp.gouv.ht/newsite/. Accessed December 10, 2020.
52. Secretaria de salud. Edades de los diagnosticados y muertos por coronavirus. Available at https://www.elheraldo.hn/pais/1381607-466/honduras-suma-3477-infectados-y-167-muertos-por-coronavirus-hay-273-nuevos. Accessed December 10, 2020.
53. Centre for health protection department of health. Latest situation of cases of covid-19. Available at https://www.chp.gov.hk/files/pdf/local_situation_covid19_en.pdf. Accessed December 7, 2020.
54. Government Information Center. Deceased. Available at https://koronavirus.gov.hu/elhunytak. Accessed December 10, 2020.
55. European Centre for Disease Prevention and Control. Data on the 14-day age-specific notification rate of new COVID-19 cases. Available at https://www.ecdc.europa.eu/en/publications-data/download-data-hospital-and-icu-admission-rates-and-current-occupancy-covid-19. Accessed December 10, 2020.
56. The directorate of health and the department of civil protection and emergency management. Covid-19 in Iceland-statistics. Available at https://www.covid.is/data. Accessed December 10, 2020.
57. Gugus tugas percepatan penanganan COVID-19. Distribution Data. Available at https://covid19.go.id/peta-sebaran. Accessed December 10, 2020.
58. Health protection surveillance centre. Epidemiology of covid-19 in Ireland. Available at https://www.hpsc.ie/a-z/respiratory/coronavirus/novelcoronavirus/casesinireland/epidemiologyofcovid-19inireland/. Accessed December 10, 2020.
59. Israel science and technology directory. Age distribution of confirmed covid-19 infected in Israel. Available at https://www.science.co.il/medical/coronacoron/Distribution-age.php. Accessed December 10, 2020.
60. Epidemiology of Public Health. COVID-19 integrated surveillance: the main national data. Available at https://www.epicentro.iss.it/coronavirus/sars-cov-2-sorveglianza-dati. Accessed December 10, 2020.
61. Ministry of health and wellness. Dashboard. Available at https://jamcovid19.moh.gov.jm/. Accessed December 10, 2020.
62. Ministry of health, labor and welfare. Press release. Available at https://www.mhlw.go.jp/stf/covid-19/kokunainohasseijoukyou.html. Accessed December 10, 2020.
63. US Embassy Fiji, Kiribati, Nauru, Tonga, and Tuvalu. COVID-19 Information. Available at https://fj.usembassy.gov/u-s-citizen-services/covid-19-informinfor/. Accessed December 10, 2020.
64. WHO Lao People's Democratic Republic. COVID-19 situation report for Lao PDR. Available at https://www.who.int/laos/internal-publications-detail/covid-19-situation-report-for-lao-pdr-19. Accessed December 10, 2020.
65. Centre for disease prevention and control. News about covid-19. (Latvian) Available at https://arkartassituacija.gov.lv/. Accessed December 10, 2020.
66. Ministry of Public Health. Coronavirus COVID-19 Lebanon Cases. Available at https://www.moph.gov.lb/maps/covid19.php. Accessed December 10, 2020.
67. Ministry of Health. COVID-19 en chiffres. Available at https://msan.gouvergouver.lu/fr/graphiques-evolution.html#sghttps://msan.gouvernement.lu/fr/grapgraphi-evolution.html#sg. Accessed December 9, 2020.
68. Serviços de saúde do governo da região administrativa especial de Macau. Epidemics information. Available at https://www.ssm.gov.mo/apps1/PreventCOVID-19/en.aspx#clg17458. Accessed December 9, 2020.
69. Ministry of Health Malawi. Malawi COVID-19 Daily Situation Report. Available at https://www.facebook.com/malawimoh/?__tn__=k*F&tn-str=k*F. Accessed December 9, 2020.
70. Kementerian Kesihatan Malaysia. Current Situation of the COVID-19 Pandemic in Malaysia. Available at https://www.moh.gov.my/. Accessed December 9, 2020.
71. Ministry of health republic of Maldives. Health protection agency covid-19 statistics dashboard. Available at https://covid19.health.gov.mv/dashboard/. Accessed December 9, 2020.
72. Government of Malta. COVID-19 infographics. Available at https://deputypdeputyprimem.gov.mt/en/health-promotion/covid-19/Pages/covid-19-infographics.aspx. Accessed December 9, 2020.
73. RMI National Disaster Management Office. 2019 Novel Corona Situation Report. Available at https://ndmo.gov.mh/resource-library/. Accessed December 9, 2020
74. Ministry of Health and Wellness. COVID-19. Available at http://www.covic19.mu/. Accessed December 7, 2020.
75. Ministry of Health. Information regarding COVID-19 cases in Mexico. Accessed at https://datos.gob.mx/busca/dataset/informacion-referente-a-casos-covid-19-en-mexico. Accessed at December 9, 2020.
76. Federated States of Micronesia Government. Federated States of Micronesia Health Center COVID-19 Survey Summary Report. Available at https://bbph.hrsa.gov/emergency-response/coronavirus-health-center-data/fm. Accessed December 9, 2020.
77. Ministry of Health, Labor and Social Protection. COVID-19 in the Republic of Moldova: current situation. (Romanian) Available at http://gismoldova.maps.arcgis.coc/apps/opsdashboard/index.html#/d274da857ed345efa66e1fbc959b021b. Accessed December 9, 2020.
78. Gourvernement princier. Information relating to Covid-19 in the Principality of Monaco. Available at https://www.gouv.mc/Action-Gouvernementale/Coronavirus-Covid-2019. Accessed December 9, 2020.
79. Ministry of Health. COVID-19 in Mongolia. Available at https://covid19.mohs.mn/. Accessed December 9, 2020.
80. Ministry of Health. COVID-19. Available at https://www.misau.gov.mz/index.php/informacao-sobre-coronavirus-covid-19. Accessed December 9, 2020.
81. Ministry of Health and Social Service Namibia. COVI-19 in Namibia. Available at https://www.facebook.com/MoHSSNamibia/. Accessed December 9.
82. WHO Namibia. Namibia COVID-19 Situation Reports. Available at https://www.afro.who.int/publications/namibia-covid-19-situation-reports. Accessed December 9, 2020.
83. WHO Nepal. Situation Update . Available at https://www.who.int/nepal/news/detail/08-12-2020-who-nepal-situation-update. Accessed December 9, 2020.
84. National Institute for Public Health and the Environment Ministry of Health, Welfare and Sport. Epidemiologische situatie COVID-19 in Nederland. Available at https://www.rivm.nl/actuele-informatie-over-coronavirus/data. Accessed December 9, 2020.
85. Ministry of health. Covid-19-current cases. Available at https://www.health.govt.nz/our-work/diseases-and-conditions/covid-19-novel-coronavirus/covid-19-current-situation/covid-19-current-cases#age. Accessed December 9, 2020.
86. Observatoria Cuidadano COVID-19 Nacaragua. COVID-19 statistics in Nicaragua. Available at https://observatorioni.org/estadisticas-covid-19-nicaragua/. Accessed December 9, 2020.
87. Nigeria CDC. An update of COVID-19 outbreak in Nigeria. Available at https://ncdc.gov.ng/diseases/sitreps/?cat=14&name=An%20update%20of%20COVID-19%20outbreak%20in%20Nigeria. Accessed December 8, 2020.
88. Ministry of health and institute of public health. coronavirus status (covid-19) in Macedonia. (Macedonian) Available at https://gdi-sk.maps.arcgis.com/apps/opsdashboard/index.html?fbclid=IwAR0Dd9MY7njiNtDkPpPt8R2SeD4pW_6TO12axwKrT4CcegckY4P4Ezt43f4#/2096bd4b051b42948ac3f5747e80c3a5. Accessed December 8, 2020.
89. Commonwealth Healthcare Corporation. Information About COVID-19. Available at https://chcc.gov.mp/coronavirusinformation.php#gsc.tab=0. Accessed December 8, 2020.
90. Ministry of Health Republic of Palau. Coronavirus Disease 2019 (COVID--19) Situation Report. Available at http://www.palauhealth.org/. Accessed December 8, 2020.
91. Ministerio de salud de la república de Panamá. COVID-19. Available at http://www.minsa.gob.pa/destacado/coronavirus-covid-19. Accessed December 8, 2020.
92. Ministry of Health PNG. COVID-19-Pandemic.Available at http://www.heahea.gov.pg/subindex.php?news=1. Accessed December 7, 2020.
93. Ministry of Health. Daily. Available at https://www.mspbs.gov.py/reportes-covid19.html. Accessed December 8, 2020.
94. Ministerio de salud del Perú. Sala situacional covid-19 Perú. Available at https://covid19.minsa.gob.pe/. Accessed December 8, 2020.
95. Republic of the Philippines department of health. View detailed case information. Available at https://www.doh.gov.ph/covid19tracker. Accessed December 8, 2020.
96. Government Website of the Republic of Poland. Coronavirus: current information and recommendations. Available at https://www.gov.pl/web/koronawirus/wykaz-zarazen-koronawirusem-sars-cov-2. Accessed December 8, 2020..
97. Direção-geral da saúde. Ponto de Situação Atual em Portugal. Available at https://covid19.min-saude.pt/ponto-de-situacao-atual-em-portugal/. Accessed December 8, 2020.
98. Government of Romania. Covid-19: date to date official data. (Romanian) Available at https://datelazi.ro/. Accessed December 7, 2020.
99. Ministry of Health. Press releases. (Romanian) Available at http://www.ms.ro/comunicate/. Accessed December 7, 2020.
100. Rwanda Biomedical Center. COVID-19 Rwanda cases. Available at https://www.rbc.gov.rw/index.php?id=707. Accessed December 8, 2020.
101. WHO Rwanda. Rwanda News, Update COVID-19. Available at https://www.afro.who.int/countries/842/news?page=0 . Accessed December 8, 2020.
102. The official website of St. Kitts and Nevis. News. Available at https://www.gov.kn/. Accessed December 7, 2020.
103. government of Saint Lucia. Saint Lucia's COVID-19 Dashboard. Available at http://www.govt.lc/. Accessed December 7, 2020.
104. The Government of Saint Vincent and the Grenadines. Ministry of Health, Wellness and the Environment. Coronavirus (COVID-19) Updates. Available at http://health.gov.vc/health/index.php/c. Accessed December 7, 2020.
105. Government of Samoa. Social Updates. Available at https://www.samoagovt.ws/. Accessed December 7, 2020.
106. Ministério de Saúde de São Tomé e Príncipe. BOLETIM DIÁRIO COVID-19. Available at http://ms.gov.st/. Accessed December 8, 2020.
107. Department of healh Republic of Seychells. COVID-19 in Seychelles. Available at http://www.health.gov.sc/. Accessed December 7, 2020.
108. Official Update of COVID -19 Situation in Singapore. Dashboard of the COVID-19 Outbreak in Singapore. Available at https://co.vid19.sg/singapore/. Accessed December 7, 2020.
109. National institute of public health of the republic of Slovenia. Daily monitoring of SARS-CoV-2 infections (covid-19). (Slovenian) Available at https://www.nijz.si/sl/dnevno-spremljanje-okuzb-s-sars-cov-2-covid-19. Accessed December 11, 2020.
110. Solomon Islands Government. Learn about Coronavirus. Available at https://solomons.gov.sb/ministry-of-health-medical-services/essential-services/learn-about-coronavirus/. Accessed December 11, 2020.
111. WHO Somalia. COVID-19 Dashboard, Somalia. Available at https://bmgf.maps.arcgis.com/apps/opsdashboard/index.html#/d0d9a939c5fa401caa3a7447e72b2017. Accessed December 11,2020.
112. National institute of communicable diseases. COVID-19 Weekly eidemiology brief South Africa. Available at https://www.nicd.ac.za/diseases-a-z-index/covid-19/surveillance-reports/. Accessed December 7, 2020.
113. Korea centers for disease control and prevention. Press release. Available at https://www.cdc.go.kr/board/board.es?mid=a30402000000&bid=0030#. Accessed December 7, 2020.
114. Ministerio de sanidad, consumo y bienestar social. COVID-19 report. Available at https://www.mscbs.gob.es/profesionales/saludPublica/ccayes/alertasActual/nCov-China/situacionActual.htm. Accessed December 7, 2020.
115. Swedish Intensive Care Register (SIR). Confirmed cases in Sweden - daily update. (Swedish) Available at https://www.folkhalsomyndigheten.se/smittskydd-beredskap/utbrott/aktuella-utbrott/covid-19/bekraftade-fall-i-sverige/. Accessed December 7, 2020.
116. Federal office of public health. Deaths in Switzerland by age group. (German) Available at https://datawrapper.dwcdn.net/IJC8v/64/. Accessed December 7, 2020.
117. Center for diseases control, ministry of health and welfare. Area, age, and gender statistical-19Cov(day)-by date of confirmation. Available at https://www.cdc.gov.tw/En. Accessed December 7, 2020.
118. Ministry of Health. Covid-19 Infected Situation Reports. Available at httph://covid19.ddc.moph.go.th/th. Accessed December 7, 2020.
119. government of Timor-Leste. News. Available at http://timor-leste.gov.tl/?p=24027&lang=en&n=1. Accessed December 7, 2020.
120. Gouvernement de la republique Togolaise. L'evolutiond au Togo en graphique. Available at https://covid19.gouv.tg/graph-evolution/. Accessed December 7, 2020.
121. Ministry of health. COVID-19 Weekly Situation Report Turkey. Available at https://covid19.saglik.gov.tr/. Accessed December 7, 2020.
122. Ministry of health. Moh Uganda: covid-19 information portal. Available at https://covid19.gou.go.ug/. Accessed December 7, 2020.
123. Government UK. National COVID-19 surveillance reports. Available at https://www.gov.uk/government/publications/national-covid-19-surveillance-reports. Accesse December 7, 2020
124. Coronavirus (COVID-19) in the UK. The weekly surveillance report in England. Available at https://public.tableau.com/profile/public.health.wales.healhe.protection#!/vizhome/RapidCOVID-19virology-Public/Headlinesummary. Accessed December 7, 2020.
125. Public Health Scotland. Total Cases By Age and Sex. Available at https://www.opendata.nhs.scot/dataset/covid-19-in-scotland/resource/19646dce-d830-4ee0-a0a9-fcec79b5ac71. Accessed December 7, 2020
126. Department of Health Northern Ireland. Summary information. Available at https://app.powerbi.com/view?r=eyJrIjoiZGYxNjYzNmUtOTlmZS00ODAxODA1YTEtMjA0NjZhMzlmN2JmIiwidCI6IjljOWEzMGRlLWQ4ZDctNGFhNZ05NjAwLTRiZTc2MjVmZjZjNSIsImMiOjh9&pageName=ReportSection6b2d9e1a6390b690b8eb. Accessed December 7, 2020.
127. NHS Test and Trace (England) and coronavirus testing (UK) statistics: 19 November to 25 November. People tested for COVID-19, including those people testing positive, by age group, Pillars 1 and 2. Available at https://www.gov.uk/government/publications/national-covid-19-surveillance-reports. Accessed December 7, 2020.
128. Ministry of health of Ukraine. the Public Health Center of Ukraine. (Ukrainian) Available at https://app.powerbi.com/view?r=eyJrIjoiMjFkMTUzZTctOGM2Ny00ZWRkLTlkMjItYmY3Yzk4NzQ4ZTYwIiwidCI6IjRmZTBiMmZjLTMwZDUtNDcwZi04ZTY4LTVkNTc1YmFiMWVmMiIsImMiOjl9. Accessed December 7, 2020.
129. Ministry of health public. Epidemiología. Available at https://www.gub.uy/ministerio-salud-publica/tematica/epidemiologia. Accessed December 7, 2020.
130. Government of the Virgin Islands of the United States. VIDOH 2020 COVID-19 Response Report. Available at https://www.covid19usvi.com/. Accessed December 7, 2020.
131. American Academy of Pediatrics. Children and COVID-19: State-Level Data Report. Available at https://services.aap.org/en/pages/2019-novel-coronavicor-covid-19-infections/children-and-covid-19-state-level-data-report/. Accessed December 7, 2020.
132. VPS. COVID-19 Data: North American Pediatric ICUs. Available at https://covid19.myvps.org/. Accessed December 7, 2020.
133. Ministry of Health. COVID19 updates. Available at https://covid19.gov.vu/. Accessed December 7, 2020.
134. Ministerio del poder popular para la salud. Covid-19 estadísticas Venezuela. Available at https://covid19.patria.org.ve/estadisticas-venezuela/. Accessed December 7, 2020.
135. Ministry of health. Covid-19 emergency strength diseases situation site. (Vietnamese) Available at https://ncov.moh.gov.vn. Accessed December 7, 2020.
136. Ministry of Health. COVID-19 situation report. Available at http://www.mohcc.gov.zw/index.php?option=com_phocadownload&view=category&id=15&Itemid=741#. Accessed December 7, 2020.
