# Supplementary material for: The differential impact of pediatric COVID-19 between high-income countries and low- and middle-income countries: A systematic review of fatality and ICU admission in children worldwide
Source: PLoS One. 2021 Jan 29;16(1):e0246326. doi: 10.1371/journal.pone.0246326 (PMC7845974; doi:10.1371/journal.pone.0246326)
Supplement: S3 Table — (DOCX) [file pone.0246326.s008.docx]

**S3 Table Study design and risk assessment for included articles from database search**

| **Authors** | Study design | Q1 | Q2 | Q3 | Q4 | Q5 | Q6 | Q7 | Q8 | Q9 | Q10 |
| --- | --- | --- | --- | --- | --- | --- | --- | --- | --- | --- | --- |
| Khesrani 2020 ^1^ | Case report | Yes | No | Yes | Yes | Yes | Yes | Yes | Yes |  |  |
| Cairoli 2020 ^2^ | Case series | Yes | Yes | Yes | Yes | Yes | Yes | Yes | Yes | Yes | Yes |
| Ibrahim 2020 ^2^ | Case series | Yes | Yes | Yes | Yes | Yes | Yes | Yes | Yes | Yes | Yes |
| Macartney 2020 ^4^ | Case series | Yes | Yes | Yes | Yes | Yes | Yes | Yes | Yes | Yes | N/A |
| Farmer 2020 ^5^ | Case series | Yes | Yes | Unclear | Yes | Yes | Yes | Yes | Yes | Yes | N/A |
| Presler 2020 ^6^ | Case series | Yes | Yes | Yes | Yes | Yes | Yes | No | No | Yes | N/A |
| Saha 2020 ^7^ | Case series | Yes | Yes | Yes | Yes | Yes | Yes | No | Yes | Yes | Yes |
| Piersigilli 2020 ^8^ | Case report | Yes | Yes | Yes | Yes | Yes | Yes | Yes | Yes |  |  |
| Garau 2020 ^9^ | Case report | Yes | Yes | Yes | Yes | Yes | Yes | Yes | Yes |  |  |
| Escalera-Antezana 2020 ^10^ | Cross sectional study | Yes | Yes | Yes | Yes | Yes | Yes | Yes | Yes | Yes | Yes |
| Escalera-Antezana 2020 ^11^ | Case series | Yes | Yes | Yes | Yes | Yes | Yes | Yes | Unclear | Yes | N/A |
| Arapovic 2020 ^12^ | Case series | Yes | Yes | Yes | Yes | Yes | Yes | No | Yes | Yes | Yes |
| Aragao 2020 ^13^ | Case report | Yes | Yes | Yes | Yes | Yes | Yes | No | Yes |  |  |
| Frank 2020 ^14^ | Case report | Yes | Yes | Yes | Yes | Yes | Yes | Yes | Yes |  |  |
| Schaan 2020 ^15^ | Case series | Yes | Yes | Yes | Yes | Yes | Yes | Yes | Yes | Yes | N/A |
| Florencio 2020 ^16^ | Case series | Yes | Yes | Yes | No | Unclear | Yes | Yes | Yes | No | N/A |
| deMirandaHenriques-Souza 2020 ^17^ | Case report | Yes | Yes | Yes | Yes | Yes | Yes | No | Yes |  |  |
| Bezerra 2020 ^18^ | Case report | Yes | Yes | Yes | Yes | Yes | Yes | Yes | Yes |  |  |
| Carvalho 2020 ^19^ | Case report | Yes | Yes | Yes | Yes | Yes | Yes | Yes | Yes |  |  |
| Almeida 2020 ^20^ | Case report | Yes | Yes | Yes | Yes | Yes | Yes | Yes | Yes |  |  |
| Giorno 2020 ^21^ | Case series | Yes | Yes | Yes | Yes | Yes | Yes | Yes | No | Yes | N/A |
| Rabha 2020 ^22^ | Case series | Yes | Yes | Yes | Yes | Yes | Yes | Yes | No | Yes | Yes |
| Silva 2020 ^23^ | Case series | Yes | Yes | Yes | Unclear | Yes | Yes | No | No | Yes | N/A |
| Prata-Barbosa 2020 ^24^ | Case series | Yes | Yes | Yes | Yes | Yes | Yes | Yes | No | Yes | Yes |
| Hillesheim 2020 ^25^ | Cross sectional study | Yes | Yes | Yes | No | No | Yes | Yes | Yes |  |  |
| Magalhaes 2020 ^26^ | Case Series | Yes | Yes | Yes | Yes | Yes | Yes | Yes | Yes | Yes | Yes |
| deSiqueira Alves Lopes 2020 ^27^ | Cross sectional study | Yes | Yes | Yes | Yes | No | No | Yes | Yes |  |  |
| Wong 2020 ^28^ | Case series | Yes | Yes | Yes | Yes | Yes | Yes | Yes | No | Yes | N/A |
| George 2020 ^28^ | Case series | Yes | Yes | Yes | Yes | Yes | Yes | Yes | Yes | Yes | N/A |
| Wong 2020 ^30^ | Cross sectional study | Yes | Yes | Yes | Yes | No | No | Yes | Yes |  |  |
| Ladha 2020 ^31^ | Case report | Yes | Yes | Yes | Yes | Yes | Yes | No | Yes |  |  |
| Kirtsman 2020 ^32^ | Case report | Yes | Yes | Yes | Yes | Yes | Yes | Yes | Yes |  |  |
| Diaz-Corvillon 2020 ^33^ | Case series | Yes | Yes | Yes | Yes | Yes | Yes | Yes | Yes | Yes | Yes |
| Geng 2020 ^34^ | Case series | Yes | Yes | Yes | Yes | Yes | Yes | Yes | Yes | Yes | N/A |
| Xu 2020 ^35^ | Case series | Yes | Yes | Yes | Yes | Yes | Yes | Yes | No | Unclear | N/A |
| Zhang 2020 ^36^ | Case series | Yes | Yes | Yes | Yes | Yes | Yes | Yes | Yes | Yes | N/A |
| Chang 2020 ^37^ | Case series | Yes | Yes | Yes | Unclear | Unclear | Yes | Yes | Yes | Yes | N/A |
| Sun 2020 ^38^ | Case series | Yes | Yes | Yes | No | Unclear | Yes | Yes | Yes | Yes | N/A |
| Zhao 2020 ^39^ | Case series | No | Yes | Yes | Yes | Yes | Yes | Yes | Yes | Yes | Yes |
| Gao 2020 ^40^ | Case series | Yes | Yes | Yes | Yes | Yes | Unclear | Yes | Yes | No | N/A |
| Liu 2020 ^41^ | Case series | Yes | Yes | Yes | Yes | Yes | Yes | Yes | Unclear | Yes | N/A |
| Liu 2020 ^42^ | Case series | Yes | Yes | Yes | Yes | Yes | Yes | No | No | Yes | Yes |
| Jiang 2020 ^43^ | Case series | No | Yes | Yes | Yes | No | Yes | Yes | No | Yes | N/A |
| Lu 2020 ^44^ | Case report | Yes | Yes | Yes | Yes | No | Yes | Yes | Yes |  |  |
| Bai 2020 ^45^ | Case series | Yes | Yes | Yes | Yes | Yes | Yes | Yes | Yes | Yes | Yes |
| Wei 2020 ^46^ | Case report | Yes | Yes | Yes | Yes | Yes | Yes | Yes | Yes |  |  |
| Zhang 2020 ^47^ | Case series | Yes | Yes | Yes | Yes | Yes | Yes | Yes | Unclear | Yes | N/A |
| Liu 2020 ^48^ | Case series | Yes | Yes | Yes | Yes | Yes | Yes | Yes | Unclear | Yes | Yes |
| Ren 2020 ^49^ | Case report | Yes | Yes | Yes | Yes | No | Yes | Yes | Yes |  |  |
| Xu 2020 ^50^ | Case series | Yes | Yes | Yes | Yes | Yes | Yes | Yes | Unclear | Yes | Yes |
| Song 2020 ^51^ | Case report | Yes | Yes | Yes | Yes | Yes | Yes | Yes | Yes |  |  |
| Yang 2020 ^52^ | Case series | Yes | Yes | Yes | Yes | Yes | Yes | Yes | Yes | Yes | N/A |
| Zhong 2020 ^53^ | Case series | Yes | Yes | Yes | Yes | Yes | Yes | Yes | Yes | Yes | N/A |
| Zhao 2020 ^54^ | Case series | Yes | Yes | Yes | Yes | Yes | Yes | Yes | Yes | Yes | Yes |
| Yang 2020 ^55^ | Case series | Yes | Yes | Yes | Yes | Yes | Yes | Yes | Yes | Unclear | Unclear |
| Gao 2020 ^56^ | Case series | Yes | Yes | Yes | Yes | Yes | Yes | No | Yes | Yes | N/A |
| Zhao 2020 ^57^ | Case series | Yes | Yes | Yes | Yes | Yes | Yes | Yes | Yes | Yes | N/A |
| Li 2020 ^58^ | Case report | Yes | Yes | Yes | Yes | Yes | Yes | Unclear | Yes |  |  |
| Jiao 2020 ^59^ | Case report | Yes | Yes | Yes | Yes | Yes | Yes | Unclear | Yes |  |  |
| Chen 2020 ^60^ | Case series | Yes | Yes | Yes | Yes | Yes | Yes | Yes | Yes | Yes | N/A |
| Sun 2020 ^61^ | Case series | Yes | Yes | Yes | Yes | Yes | Yes | Yes | Yes | Yes | N/A |
| Chen 2020 ^62^ | Case series | Yes | Yes | Yes | Yes | Yes | Yes | Yes | Yes | Yes | Yes |
| Qian 2020 ^63^ | Case series | Yes | Yes | Yes | Yes | N/A | Yes | Yes | Yes | Yes | N/A |
| Xing 2020 ^64^ | Case series | Yes | Yes | Yes | Yes | Yes | Yes | Yes | No | Yes | N/A |
| Jia 2020 ^65^ | Case series | Yes | Yes | Yes | Yes | Yes | Yes | Yes | Yes | Yes | Yes |
| Wang 2020 ^66^ | Case series | Yes | Yes | Yes | Yes | Yes | Yes | Yes | Yes | Yes | Yes |
| Wang 2020 ^67^ | Case series | Yes | Yes | Yes | Yes | Yes | Yes | Yes | Yes | Yes | Yes |
| Liu 2020 ^68^ | Case series | Yes | Yes | Yes | Yes | Yes | Yes | Yes | Yes | Yes | Yes |
| ji 2020 ^69^ | Case series | Yes | Yes | Yes | Yes | Yes | Yes | Yes | Yes | Yes | N/A |
| Feng 2020 ^70^ | Case series | Yes | Yes | Yes | Yes | Yes | Yes | Yes | Yes | Yes | Yes |
| Li 2020 ^71^ | Case report | Yes | Yes | Yes | Yes | Yes | Yes | Yes | Yes |  |  |
| Zhang 2020 ^72^ | Case series | Unclear | Yes | Yes | Yes | Yes | Yes | Yes | Yes | N/A | N/A |
| Lei 2020 ^73^ | Case series | Yes | Yes | Yes | Yes | Yes | Yes | Unclear | Unclear | Yes | N/A |
| Wu 2020 ^74^ | Case series | Yes | Yes | Yes | Yes | Yes | Yes | Yes | Yes | Yes | N/A |
| Wu 2020 ^75^ | Case series | Yes | Yes | Yes | Yes | Yes | Yes | Yes | Yes | Yes | Yes |
| Liu 2020 ^76^ | Case series | Yes | Yes | Yes | Yes | Yes | Yes | Yes | Yes | Yes | Yes |
| Lan 2020 ^77^ | Case series | Yes | Yes | Yes | Yes | Yes | Yes | Yes | Yes | Yes | N/A |
| Sun 2020 ^78^ | Case series | Yes | Yes | Yes | Yes | Yes | Yes | Yes | Yes | Yes | N/A |
| Lu 2020 ^79^ | Case series | Yes | Yes | Yes | Yes | Yes | Yes | Yes | Yes | Yes | N/A |
| Zeng 2020 ^80^ | Case series | Yes | Yes | Yes | Yes | Yes | Yes | Yes | Yes | Yes | N/A |
| Jiang 2020 ^81^ | Case series | Yes | Yes | Yes | Yes | Yes | Yes | Yes | No | N/A | N/A |
| Liu 2020 ^82^ | Case series | Yes | Yes | Yes | Yes | Yes | Yes | Yes | Yes | Yes | N/A |
| Wang 2020 ^83^ | Case series | Yes | Yes | Yes | Yes | Yes | Yes | Yes | Yes | Yes | Yes |
| Wang 2020 ^84^ | Case series | Yes | Yes | Yes | Yes | Yes | Yes | Yes | Yes | Yes | Yes |
| Gao 2020 ^85^ | Case series | Yes | Yes | Yes | N/A | Yes | Yes | Yes | Yes | No | N/A |
| Zhang 2020 ^86^ | Case series | Yes | Yes | Yes | N/A | N/A | Yes | Yes | Yes | Yes | N/A |
| Li 2020 ^87^ | Case series | Yes | Yes | Yes | Yes | Yes | Yes | Yes | Yes | Yes | N/A |
| Chen 2020 ^88^ | Case series | Yes | Yes | No | Yes | Yes | Yes | Yes | Yes | Yes | N/A |
| Zheng 2020 ^89^ | Case series | Yes | Yes | Yes | Yes | Yes | Yes | Yes | Yes | Yes | Yes |
| Li 2020 ^89^ | Case report | Yes | Yes | Yes | Yes | Yes | Yes | No | Yes |  |  |
| Li 2020 ^91^ | Case series | Yes | Yes | Yes | Yes | Yes | Yes | Yes | Yes | Unclear | Yes |
| Wu 2020 ^92^ | Case series | Yes | Yes | Yes | Yes | Yes | Yes | Yes | Yes | Yes | Yes |
| Li 202 ^93^ | Case series | Yes | Yes | Yes | Yes | Yes | Yes | Yes | No | Yes | N/A |
| Wu 2020 ^94^ | Case report | Yes | Yes | Yes | Yes | Yes | Yes | N/A | Yes |  |  |
| Li 2020 ^95^ | Case series | Yes | Yes | Yes | Yes | Yes | Yes | Yes | Yes | Yes | Yes |
| Wei 2020 ^96^ | Case series | Yes | Yes | Yes | Yes | Yes | No | Yes | Yes | Yes | Yes |
| Sun 2020 ^97^ | Case series | Yes | Yes | Yes | Yes | Yes | Yes | Yes | Yes | Yes | N/A |
| Li 2020 ^98^ | Case series | Unclear | Yes | Yes | Unclear | Unclear | Yes | Yes | Yes | Yes | N/A |
| Yu 2020 ^99^ | Case report | Yes | Yes | Yes | Yes | Yes | Yes | Yes | Yes |  |  |
| Tang 2020 ^100^ | Case report | Yes | Yes | Yes | Yes | Yes | Yes | Yes | Yes |  |  |
| Li 2020 ^101^ | Case series | Yes | Yes | Yes | Yes | Yes | Yes | Yes | Yes | Yes | Yes |
| Zhu 2020 ^102^ | Case series | Unclear | Yes | Yes | Unclear | Unclear | Yes | Yes | No | Yes | N/A |
| Zheng 2020 ^103^ | Case series | Yes | Yes | Yes | Yes | Yes | Yes | Yes | Yes | Yes | Yes |
| Fu 2020 ^104^ | Case series | Yes | Yes | Yes | Yes | Yes | Yes | Yes | Yes | Yes | N/A |
| Yuxia Cui 2020 ^105^ | Case report | Yes | Yes | Yes | Yes | Yes | Yes | Yes | Yes |  |  |
| Zhu 2020 ^106^ | Case series | Yes | Yes | Yes | Yes | Yes | Yes | Yes | Yes | No | N/A |
| Wu 2020 ^107^ | Case series | Yes | Yes | Yes | Yes | Yes | Yes | Yes | Yes | No | N/A |
| Wang 2020 ^108^ | Case series | Yes | Yes | Yes | Yes | Yes | Yes | Yes | Yes | Unclear | N/A |
| Lin 2020 ^109^ | Case series | Yes | Yes | Yes | Yes | Yes | Yes | Yes | Unclear | Yes | Yes |
| Song 2020 ^110^ | Case series | Yes | Yes | Yes | Yes | Yes | Yes | Unclear | Yes | Yes | N/A |
| Tan 2020 ^111^ | Case series | Yes | Yes | Yes | Yes | Yes | Yes | Yes | Yes | Yes | N/A |
| Xu 2020 ^112^ | Case series | Yes | Yes | Yes | Yes | Yes | Yes | Yes | Yes | Yes | Yes |
| Lei 2020 ^113^ | Case series | Yes | Yes | Yes | Yes | Yes | Yes | Yes | No | Yes | Yes |
| Huang 2020 ^114^ | Case series | Yes | Yes | Yes | Yes | Yes | Yes | Yes | Yes | Yes | N/A |
| Su 2020 ^115^ | Case series | Yes | Yes | Yes | Yes | Yes | Yes | Yes | Yes | Yes | Yes |
| Du 2020 ^116^ | Case series | Yes | Yes | Yes | Yes | Yes | Yes | Yes | No | Yes | Yes |
| Fan 2020 ^117^ | Case report | Yes | Yes | Yes | Yes | Yes | Yes | N/A | Yes |  |  |
| Wang 2020 ^118^ | Case series | Yes | Yes | Yes | Yes | Yes | Yes | Yes | No | Yes | Yes |
| Chan 2020 ^119^ | Case series | Yes | Yes | Yes | Yes | Unclear | Yes | Yes | Yes | Yes | N/A |
| Guiqing 2020 ^120^ | Case report | Yes | Yes | Yes | Yes | Yes | Yes | Unclear | Yes |  |  |
| Zheng 2020 ^121^ | Case series | Yes | Yes | Yes | Yes | Yes | Yes | Yes | Yes | Yes | N/A |
| Chen 2020 ^122^ | Case series | Yes | Yes | Yes | Yes | Unclear | Yes | Yes | Yes | Yes | N/A |
| Liu 2020 ^123^ | Case series | Yes | Yes | Yes | Yes | Yes | Yes | Yes | Yes | Unclear | N/A |
| Han 2020 ^124^ | Case series | Unclear | Yes | Yes | Yes | Yes | Yes | Yes | Yes | Yes | Yes |
| Song 2020 ^125^ | Case series | Yes | Yes | Yes | Yes | Yes | Yes | Yes | Yes | Yes | N/A |
| Lou 2020 ^126^ | Case series | Yes | Yes | Yes | Unclear | Unclear | Yes | Yes | Yes | Yes | N/A |
| Li 2020 ^127^ | Case series | Yes | Yes | Yes | Yes | Yes | Yes | Unclear | Unclear | Yes | N/A |
| Li 2020 ^128^ | Case series | Yes | Yes | Yes | Yes | Yes | Yes | Yes | Yes | Yes | N/A |
| Lin 2020 ^129^ | Case report | Yes | Yes | Yes | Yes | Yes | No | No | Yes |  |  |
| Xu 2020 ^130^ | Case series | Unclear | Yes | Yes | Yes | Unclear | Yes | Yes | Unclear | Yes | N/A |
| Lo 2020 ^131^ | Case series | Yes | Yes | Yes | Yes | Yes | Yes | Yes | Yes | Yes | Yes |
| Dong 2020 ^312^ | Case series | Yes | Yes | Yes | Yes | Yes | Yes | No | No | Yes | Yes |
| Lendorf 2020 ^133^ | Case series | Yes | Yes | Yes | Yes | Yes | Yes | Yes | Yes | Yes | Yes |
| Ali 2020 ^134^ | Case series | Yes | Yes | Yes | Yes | Yes | Yes | Yes | Unclear | Yes | Yes |
| Nofal 2020 ^135^ | Case series | Yes | Yes | Yes | Yes | Yes | Yes | Yes | Yes | Yes | N/A |
| Ministerio de Salud 2020 ^136^ | Case series | Unclear | Unclear | Unclear | Yes | Yes | Yes | No | No | Unclear | N/A |
| Gotzinger 2020 ^137^ | Case series | Yes | Yes | Yes | Yes | Yes | Yes | Yes | Yes | Yes | Yes |
| Martenot 2020 ^138^ | Case series | Yes | Yes | Yes | Yes | Yes | Yes | Yes | Yes | Yes | N/A |
| Tchidjou 2020 ^139^ | Case report | Yes | Yes | Yes | Yes | Yes | Yes | Unclear | Yes |  |  |
| Tchidjou 2020 ^140^ | Case report | Yes | Yes | Yes | Yes | No | Yes | Yes | Yes |  |  |
| Danis 2020 ^141^ | Case series | Yes | Yes | Yes | N/A | Yes | Yes | Yes | Yes | Yes | N/A |
| LeRoux 2020 ^142^ | Case report | Yes | Yes | Yes | Yes | Yes | Yes | No | Yes |  |  |
| Morand 2020 ^143^ | Case report | Yes | Yes | Yes | Yes | Yes | Yes | No | Yes |  |  |
| Aherfi 2020 ^144^ | Case series | Yes | Yes | Yes | Yes | Yes | Yes | No | No | Yes | N/A |
| Colson 2020 ^145^ | Case series | Yes | Yes | Yes | Yes | Yes | Yes | No | No | Yes | Yes |
| Abasse 2020 ^146^ | Case report | Yes | Yes | Yes | Yes | Yes | Yes | No | Yes |  |  |
| Heilbronner 2020 ^147^ | Case series | Yes | Yes | Yes | Yes | Yes | Yes | Yes | Yes | Yes | N/A |
| Vivanti 2020 ^148^ | Case report | Yes | Yes | Yes | Yes | Yes | Yes | Yes | Yes |  |  |
| Nathan 2020 ^149^ | Case series | Yes | Yes | Yes | Yes | Yes | Yes | Yes | Yes | Yes | N/A |
| Fischer 2020 ^150^ | Case report | Yes | Yes | Yes | Yes | Yes | Yes | No | Yes |  |  |
| Nathan 2020 ^151^ | Case series | Yes | Yes | Yes | Yes | Yes | Yes | Yes | Yes | Yes | N.A |
| Oualha 2020 ^152^ | Case series | Yes | Yes | Yes | Yes | Yes | Yes | Yes | Yes | Yes | Yes |
| Gaborieau 2020 ^153^ | Case series | Yes | Yes | Yes | Yes | Yes | Yes | Yes | Yes | Yes | Yes |
| Meslin 2020 ^154^ | Case series | Yes | Yes | Yes | Yes | Yes | Yes | Yes | Yes | Yes | N/A |
| Loron 2020 ^155^ | Case report | Yes | Yes | Yes | Yes | Yes | Yes | Yes | Yes |  |  |
| Nazon 2020 ^156^ | Case report | Yes | Yes | Yes | Yes | Yes | Yes | No | Yes |  |  |
| Lorenz 2020 ^157^ | Case report | Yes | Yes | Yes | Yes | Yes | Yes | Yes | Yes |  |  |
| Olfe 2020 ^158^ | Case report | Yes | Yes | Yes | Yes | Yes | Yes | Yes | Yes |  |  |
| Wehl 2020 ^159^ | Case report | Yes | Yes | Yes | Yes | Yes | Yes | Unclear | Yes |  |  |
| Kim-Hellmuth 2020 ^160^ | Case report | Yes | Yes | Yes | Yes | Yes | Yes | No | Yes |  |  |
| Farber 2020 ^161^ | Case report | Yes | Yes | Yes | Yes | Yes | Yes | Unclear | Yes |  |  |
| Koczulla 2020 ^162^ | Case series | Yes | Yes | Yes | Yes | Yes | Yes | Yes | Yes | Yes | N/A |
| Wolf 2020 ^163^ | Case series | Yes | Yes | Yes | Yes | Yes | Yes | Yes | Yes | Yes | N/A |
| Armann 2020 ^164^ | Case series | Yes | Yes | Yes | Yes | Yes | Yes | Yes | Yes | Yes | Yes |
| Ayisi-Boateng 2020 ^165^ | Case series | Unclear | Yes | Yes | Unclear | Yes | Yes | Yes | No | Yes | N/A |
| Maltezou 2020 ^166^ | Case series | Yes | Yes | Yes | Yes | Yes | Yes | Yes | Yes | Yes | Yes |
| Maltezou 2020 ^167^ | Case Series | Yes | Yes | Yes | Yes | Yes | Yes | Yes | Yes | No | Yes |
| Majachani 2020 ^168^ | Case report | Yes | Yes | Yes | Yes | Yes | Yes | No | Yes |  |  |
| Mak 2020 ^169^ | Case series | Yes | Yes | Yes | Yes | Yes | Yes | Yes | Yes | Yes | N/A |
| Rost 2020 ^170^ | Cross sectional study | Yes | Yes | Unclear | Yes | Yes | Yes | Yes | Yes |  |  |
| Dosi 2020 ^171^ | Case series | Yes | Yes | Yes | Yes | Yes | Yes | Unclear | No | Yes | Yes |
| Singh 2020 ^172^ | Case report | Yes | Yes | Yes | Yes | Yes | Yes | No | Yes |  |  |
| Kant 2020 ^173^ | Case series | Yes | Yes | Yes | Yes | Yes | Yes | Yes | No | Yes | Yes |
| Meghwal 2020 ^174^ | Case series | Yes | Yes | Yes | Yes | Yes | Yes | Unclear | No | Yes | Yes |
| Mohakud 2020 ^175^ | Case report | Yes | Yes | Yes | Yes | Yes | Yes | No | Yes |  |  |
| Radhakrishnan 2020 ^176^ | Case series | Yes | Yes | Yes | Yes | Yes | Yes | Yes | Yes | Yes | N/A |
| Daniel 2020 ^177^ | Case report | Yes | Yes | Yes | Yes | Yes | Yes | No | Yes |  |  |
| Nayak 2020 ^178^ | Case series | Yes | Yes | Yes | Yes | Yes | Yes | Yes | Yes | Yes | N/A |
| Kalamdani 2020 ^179^ | Case series | Yes | Yes | Yes | Yes | Yes | Yes | No | Yes | Yes | Yes |
| Gupta 2020 ^180^ | Case series | Yes | Yes | Yes | Yes | Yes | Yes | Yes | Yes | Yes | N/A |
| Bandyopadhyay 2020 ^181^ | Case report | Yes | Yes | Yes | Yes | Yes | No | No | Yes |  |  |
| Anand 2020 ^182^ | Case series | Yes | Yes | Yes | Yes | Yes | Yes | Yes | Yes | Yes | N/A |
| Shankar 2020 ^183^ | Case series | Yes | Yes | Yes | Yes | Yes | Yes | Yes | Yes | Yes | N/A |
| Kulkarni 2020 ^184^ | Case report | Yes | Yes | Yes | Yes | Yes | Yes | No | Yes |  |  |
| Kulkarni 2020 ^3185^ | Case report | Yes | Yes | Yes | Yes | Yes | Yes | Unclear | Yes |  |  |
| Sarangi 2020 ^186^ | Cross sectional study | Yes | Yes | Yes | Yes | Unclear | Yes | Yes | Yes |  |  |
| Saraswathi 2020 ^187^ | Case series | Yes | Yes | Yes | Yes | Yes | Yes | No | No | Yes | Yes |
| Gupta 2020 ^188^ | Case series | Yes | Yes | Yes | Yes | Yes | Unclear | Yes | Yes | Yes | N/A |
| Laxminarayan 2020 ^189^ | Cross sectional study | Yes | Yes | Yes | Yes | Yes | Yes | Yes | Yes |  |  |
| Banerjee 2020 ^190^ | Case series | Yes | Yes | Yes | Yes | Yes | Yes | Yes | No | Yes | Yes |
| Marhaeni 2020 ^191^ | Case report | Yes | Yes | Yes | Yes | Yes | Yes | No | Yes |  |  |
| Sumarni 2020 ^192^ | Case report | Yes | Yes | Yes | Yes | Yes | Yes | No | Yes |  |  |
| EsmaeiliDooki 2020 ^193^ | Case series | Yes | Yes | Yes | Yes | Yes | Yes | No | No | Yes | Yes |
| Mirzaee 2020 ^194^ | Case report | Yes | Yes | No | Yes | Yes | Yes | No | Yes |  |  |
| Hashemi 2020 ^195^ | Case series | Unclear | Yes | Yes | Unclear | Unclear | Yes | Yes | Yes | Yes | N/A |
| Noghabi 2020 ^196^ | Case report | Yes | Yes | Yes | Yes | Yes | Yes | No | Yes |  |  |
| Gharekhanloo 2020 ^197^ | Case series | Yes | Yes | Yes | Yes | Yes | Yes | No | Yes | Yes | Yes |
| Norooznezhad 2020 ^198^ | Case series | Yes | Yes | Yes | Yes | Yes | Yes | No | Yes | Yes | Yes |
| Mamishi 2020 ^199^ | Case series | Yes | Yes | Yes | Yes | Yes | Yes | No | No | Yes | Yes |
| Moradveisi 2020 ^200^ | Case report | Yes | Yes | Yes | Yes | Yes | Yes | No | Yes |  |  |
| Navaeifar 2020 ^201^ | Case report | Yes | Yes | Yes | Yes | Yes | Yes | No | Yes |  |  |
| Malekhosseini 2020 ^202^ | Case series | Yes | Yes | Yes | Yes | Yes | Yes | No | Yes | Yes | Yes |
| Emami 2020 ^203^ | Case series | Yes | Yes | Yes | Yes | Yes | Yes | Yes | No | Yes | N/A |
| Nikoupour 2020 ^204^ | Case report | Yes | Yes | Yes | Yes | Yes | Yes | No | Yes |  |  |
| Saeed 2020 ^205^ | Case report | Yes | Yes | Yes | Yes | Yes | Yes | No | Yes |  |  |
| Saeed 2020 ^206^ | Case report | Yes | Yes | Yes | Yes | Yes | Yes | No | Yes |  |  |
| Ekbatani 2020 ^207^ | Case series | Unclear | Yes | Yes | N/A | Unclear | Yes | Yes | Yes | Yes | N/A |
| Nikpouraghdam 2020 ^208^ | Case series | Yes | Yes | Yes | Yes | Yes | Yes | Yes | Yes | Yes | Yes |
| Jafari 2020 ^209^ | Case report | Yes | Yes | Yes | Yes | Yes | Yes | N/A | Yes |  |  |
| Eghbali 2020 ^210^ | Case series | Yes | Yes | Yes | Unclear | Unclear | Yes | Yes | Yes | Yes | N/A |
| Karimi 2020 ^211^ | Case report | Yes | Yes | Yes | Yes | Yes | Yes | No | Yes |  |  |
| Rabizadeh 2020 ^212^ | Case report | Yes | Yes | Yes | Yes | Yes | Yes | No | Yes |  |  |
| Rohani 2021 ^213^ | Case report | Yes | Yes | Yes | Yes | Yes | Yes | No | Yes |  |  |
| HajiEsmaeilMemar 2020 ^214^ | Case report | Yes | Yes | Yes | Yes | Yes | Yes | Unclear | Yes |  |  |
| Dorgalaleh 2020 ^215^ | Case report | Yes | Yes | Yes | Yes | Yes | Yes | No | Yes |  |  |
| Mahmoudi 2020 ^216^ | Case report | Yes | Yes | Yes | Yes | Yes | Yes | No | Yes |  |  |
| Kalantari 2020 ^217^ | Case series | Yes | Yes | Yes | Yes | Yes | Yes | No | No | Yes | Yes |
| Nasimfar 2020 ^218^ | Case report | Yes | Yes | Yes | Yes | Yes | Yes | No | Yes |  |  |
| Soltani 2020 ^219^ | Case series | Yes | Yes | Yes | Yes | Yes | Yes | Unclear | Yes | Unclear | N/A |
| Schwartz 2020 ^220^ | Case series | Yes | Yes | Yes | Yes | Yes | Yes | Yes | Yes | Yes | N/A |
| Hashemi 2020 ^221^ | Case series | Yes | Yes | Yes | Yes | Yes | Yes | Unclear | No | Yes | Yes |
| Merza 2020 ^222^ | Case series | Yes | Yes | Yes | Yes | Yes | Yes | Yes | Yes | Yes | Yes |
| Hussein 2020 ^223^ | Case series | Yes | Yes | Yes | Yes | Yes | Yes | No | Yes | Yes | Yes |
| Linnane 2020 ^224^ | Case report | Yes | Yes | Yes | Yes | Yes | Yes | Yes | Yes |  |  |
| Jacobi 2020 ^225^ | Case report | Yes | Yes | Yes | Yes | Yes | Yes | No | Yes |  |  |
| Lopian 2020 ^226^ | Case Series | Yes | Yes | Yes | Yes | Yes | Yes | Yes | Yes | Yes | Yes |
| Stein-Zamir 2020 ^227^ | Case series | Yes | Yes | Yes | Yes | Yes | Yes | Unclear | No | Yes | Yes |
| Nunziata 2020 ^228^ | Case series | Yes | Yes | Yes | Yes | Yes | Yes | No | No | Yes | N/A |
| Vergine 2020 ^229^ | Case series | Yes | Yes | Yes | Yes | Yes | Yes | Yes | No | Yes | Yes |
| Parri 2020 ^230^ | Case Series | Yes | Yes | Yes | Yes | Yes | Yes | No | Yes | Yes | Yes |
| Parri 2020 ^231^ | Case series | Yes | Yes | Yes | Yes | Yes | Yes | Yes | Yes | Yes | Yes |
| BelliNo 2020 ^232^ | Cross sectional study | Yes | Yes | Yes | Yes | Yes | Yes | Yes | Yes |  |  |
| Garazzino 2020 ^233^ | Case series | Yes | Yes | Yes | Yes | Yes | Yes | Yes | Yes | Yes | Yes |
| Irie 2020 ^234^ | Case Series | Yes | Yes | Yes | Yes | Yes | Yes | Yes | Yes | No | N/A |
| Kawamura 2020 ^235^ | Case series | Yes | Yes | Yes | Yes | Yes | Yes | Yes | Yes | Yes | N/A |
| Kakuya 2020 ^236^ | Case series | Yes | Yes | Yes | Yes | Yes | No | Yes | Yes | Yes | N/A |
| Ishii 2020 ^237^ | Case series | Yes | Yes | Yes | Yes | Yes | Yes | Yes | No | Yes | Yes |
| Kasuga 2020 ^238^ | Case series | Yes | Yes | Yes | Yes | Yes | Yes | No | No | Yes | N/A |
| Higuchi 2020 ^238^ | Case series | Yes | Yes | Yes | Yes | Yes | Yes | Yes | Yes | Yes | N/A |
| Sano 2020 ^240^ | Case Report | Yes | Yes | Yes | Yes | Yes | Yes | No | Yes |  |  |
| Alkhatatbeh 2020 ^241^ | Case series | Yes | Yes | Yes | Yes | Yes | Yes | No | No | Yes | Yes |
| Kilani 2020 ^242^ | Case Series | Yes | Yes | Yes | Yes | Yes | Yes | No | Yes | Yes | Yes |
| Yusef 2020 ^243^ | Case series | Yes | Yes | Yes | Yes | Yes | Yes | No | No | Yes | N/A |
| Semenova 2020 ^244^ | Cross sectional study | Yes | Yes | Unclear | Yes | Yes | Yes | Yes | Yes |  |  |
| Savic 2020 ^245^ | Case report | Yes | Yes | Yes | Yes | Yes | No | Yes | Yes |  |  |
| Ayed 2020 ^246^ | Case series | Yes | Yes | Yes | Yes | Yes | Yes | Yes | Yes | Yes | N/A |
| Alsharrah 2020 ^247^ | Case Series | Yes | Yes | Yes | Yes | Yes | Yes | Yes | Yes | Yes | Yes |
| Mansour 2020 ^248^ | Case report | Yes | Yes | Yes | Yes | Yes | Yes | No | Yes |  |  |
| Oberweis 2020 ^249^ | Case report | Yes | Yes | Yes | Yes | Yes | Yes | Unclear | Yes |  |  |
| Vee 2020 ^250^ | Case series | Yes | Yes | Yes | Yes | Yes | Yes | Yes | No | Yes | Yes |
| See 2020 ^251^ | Case series | Yes | Yes | Yes | Unclear | Unclear | Yes | Yes | Yes | No | N/A |
| Castano-Jaramillo 2020 ^252^ | Case report | Yes | Yes | Yes | Yes | Yes | Yes | Yes | Yes |  |  |
| Flores 2020 ^253^ | Case series | Yes | Yes | Yes | Yes | Yes | Yes | Yes | Yes | Yes | N/A |
| Hinojosa-Velasco 2020 ^254^ | Case report | Yes | Yes | Yes | Yes | Yes | Yes | No | Yes |  |  |
| Olivar-Lopez 2020 ^255^ | Case Series | Yes | Yes | Yes | Yes | Yes | Yes | Yes | Yes | Yes | Yes |
| Lahfaoui 2020 ^256^ | Case report | Yes | Yes | Yes | Yes | Yes | Yes | Yes | Yes |  |  |
| Chekhlabi 2020 ^257^ | Case series | Yes | Yes | Yes | Yes | Yes | Yes | Unclear | Yes | Yes | N/A |
| Nassih 2020 ^258^ | Case report | Yes | Yes | Yes | Yes | Yes | Yes | Yes | Yes |  |  |
| Fakiri 2020 ^259^ | Case series | Yes | Yes | Yes | Yes | Yes | Yes | Yes | Yes | Yes | N/A |
| Janah 2020 ^260^ | Case series | No | No | No | Yes | Yes | Yes | Yes | No | Yes | N/A |
| deSanctis 2020 ^261^ | Case series | Yes | Yes | Yes | Yes | Yes | Yes | Yes | Yes | Yes | N/A |
| Caro-Dominguez 2020 ^262^ | Case series | Yes | Yes | Yes | Yes | Yes | Yes | No | Yes | Yes | Yes |
| Sola 2020 ^263^ | Case series | Yes | Yes | Yes | Yes | Yes | Yes | Unclear | Yes | Unclear | N/A |
| Slaats 2020 ^264^ | Case Report | Yes | Yes | Yes | Yes | Yes | Yes | No | Yes |  |  |
| Soumana 2020 ^265^ | Case report | Yes | Yes | Yes | Yes | Yes | Yes | No | Yes |  |  |
| Ibrahim 2020 ^266^ | Case series | Yes | Yes | Yes | Yes | Yes | Yes | Yes | No | Yes | N/A |
| Adedeji 2020 ^267^ | Cross sectional study | Yes | Yes | Yes | Yes | No | No | Yes | Yes |  |  |
| Stordal 2020 ^268^ | Cross sectional study | Yes | Yes | Yes | Yes | No | No | Yes | Yes |  |  |
| Kristoffersen 2020 ^269^ | Case Reports | Yes | Yes | Yes | Yes | Yes | Yes | Unclear | Yes |  |  |
| Alwardi 2020 ^270^ | Case series | Yes | Yes | Yes | Yes | Yes | Yes | Yes | Yes | Yes | N/A |
| Moazzam 2020 ^271^ | Case report | Yes | Yes | Yes | Yes | Yes | Yes | Unclear | Yes |  |  |
| Alzamora 2020 ^272^ | Case report | Yes | Yes | Yes | Yes | Yes | Yes | Yes | Yes |  |  |
| Conto-Palomino 2020 ^273^ | Case report | Yes | Yes | Yes | Yes | Yes | Yes | Yes | Yes |  |  |
| Rodriguez-Portilla 2020 ^274^ | Case report | Yes | Yes | Yes | Yes | Yes | Yes | Unclear | Yes |  |  |
| Montoya 2020 ^275^ | Case series | Yes | Yes | Yes | Yes | Yes | No | No | Yes | Yes | Yes |
| Gujski 2020 ^276^ | Case series | Yes | Yes | Yes | Yes | Yes | Unclear | Unclear | Unclear | Yes | Yes |
| Jarmolinski 2020 ^277^ | Case report | Yes | Yes | Yes | Yes | Yes | Yes | Yes | Yes |  |  |
| Correia 2020 ^278^ | Case report | Yes | Yes | Yes | Yes | Yes | Yes | No | Yes |  |  |
| PicaodeCarvalho 2020 ^279^ | Case series | Yes | Yes | Yes | Yes | Yes | Yes | Yes | Yes | Yes | Yes |
| Omrani 2020 ^280^ | Case series | Yes | Yes | Yes | Yes | Yes | Yes | Yes | No | Yes | Yes |
| AlKuwari 2020 ^281^ | Case series | Yes | Yes | Yes | Yes | Yes | Yes | Unclear | Yes | Yes | Yes |
| Soliman 2020 ^282^ | Case Report | Yes | Yes | Yes | Yes | Yes | Yes | No | Yes |  |  |
| Marginean 2020 ^283^ | Case series | Yes | Yes | Yes | Yes | Yes | Yes | No | Yes | Yes | N/A |
| Dima 2020 ^284^ | Case Series | Yes | Yes | Yes | Yes | Yes | Yes | Yes | Yes | Yes | Yes |
| PshenisNov 2020 ^285^ | Case series | Yes | Yes | Yes | Yes | Yes | Yes | No | Yes | Yes | Yes |
| Vashukova 2020 ^286^ | Case series | Yes | Yes | Yes | Yes | Yes | Yes | Yes | Yes | Yes | N/A |
| Spichak 2020 ^287^ | Case Reports | Yes | Yes | Yes | Yes | Yes | Yes | Unclear | Yes |  |  |
| Olisova 2020 ^288^ | Case report | Yes | Yes | Yes | Yes | Yes | No | No | Yes |  |  |
| Rusinova 2020 ^289^ | Case series | Yes | Yes | Yes | Yes | Yes | Yes | No | No | Yes | N/A |
| Dondurey 2020 ^290^ | Case Series | Yes | Yes | Yes | Yes | Yes | Yes | Yes | Yes | Yes | Yes |
| Meskina 2020 ^291^ | Case Series | Yes | Yes | Yes | Yes | Yes | Yes | Yes | No | Yes | Yes |
| Balashov 2020 ^292^ | Case report | Yes | Yes | Yes | Yes | Yes | Yes | No | Yes |  |  |
| Mazankova 2020 ^293^ | Case series | Yes | Yes | Yes | Yes | Yes | Yes | No | No | No | N/A |
| Uskov 2020 ^204^ | Case series | Yes | Yes | Yes | Yes | Yes | Yes | No | Yes | No | N/A |
| Elbehery 2020 ^295^ | Case report | Yes | Yes | Yes | Yes | Yes | Yes | No | Yes |  |  |
| Algadeeb 2020 ^296^ | Case report | Yes | Yes | Yes | Yes | Yes | Yes | Yes | Yes |  |  |
| Khalifa 2020 ^297^ | Case report | Yes | Yes | Yes | Yes | Yes | Yes | No | Yes |  |  |
| Khalifa 2020 ^298^ | Case Report | Yes | Yes | Yes | Yes | Yes | Yes | No | Yes |  |  |
| Alsuwailem 2020 ^299^ | Case report | Yes | Yes | Yes | Yes | Yes | No | No | Yes |  |  |
| Al-Hebshi 2020 ^300^ | Case series | Yes | Yes | Yes | Yes | Yes | Yes | No | Yes | Yes | Yes |
| Haroon 2020 ^301^ | Case series | Yes | Yes | Yes | Yes | Yes | Yes | Yes | Yes | Yes | N/A |
| Faqeeh 2020 ^302^ | Case report | Yes | Yes | Yes | Yes | Yes | Yes | No | Yes |  |  |
| Al-Omari 2020 ^303^ | Case series | Yes | Yes | Yes | Yes | Yes | Yes | Yes | Yes | Yes | Yes |
| Wong 2020 ^304^ | Case series | Yes | Yes | Yes | Yes | Yes | No | No | No | Unclear | N/A |
| Ng 2020 ^305^ | Case series | Yes | Yes | Yes | N/A | Yes | Yes | Yes | Yes | N/A | N/A |
| Kam 2020 ^306^ | Case report | Yes | Yes | Yes | Yes | Yes | No | No | Yes |  |  |
| Li 2020 ^307^ | Case Series | Yes | Yes | Yes | Yes | Yes | Yes | Yes | Yes | Yes | N/A |
| Goussard 2020 ^308^ | Case report | Yes | Yes | Yes | Yes | Yes | Yes | No | Yes |  |  |
| Lee 2020 ^309^ | Case series | Yes | Yes | Yes | No | Yes | Yes | No | No | Yes | No |
| Kim 2020 ^310^ | Case series | Yes | Yes | Yes | Yes | Yes | Yes | Yes | Yes | Yes | N/A |
| Yoo 2020 ^311^ | Case report | Yes | Yes | Yes | Yes | Yes | Yes | Unclear | Yes |  |  |
| Cho 2020 ^312^ | Case series | Yes | Yes | Yes | Yes | Yes | Yes | Yes | Yes | Yes | N/A |
| Park 2020 ^313^ | Case report | Yes | Yes | Yes | Yes | Unclear | Unclear | N/A | Yes |  |  |
| Han 2020 ^314^ | Case series | Yes | Yes | Yes | Yes | Yes | Yes | Yes | No | Yes | N/A |
| Yoon 2020 ^315^ | Case report | Yes | Yes | Yes | Yes | Yes | Yes | No | Yes |  |  |
| Han 2020 ^316^ | Case Series | Yes | Yes | Yes | Yes | Yes | Yes | Yes | No | No | Yes |
| Sanchez Tierraseca 2020 ^317^ | Case report | Yes | Yes | Yes | Yes | Yes | Yes | Yes | Yes |  |  |
| Gine 2020 ^318^ | Case report | Yes | Yes | Yes | Yes | Yes | Yes | Unclear | Yes |  |  |
| VelascoPuyo 2020 ^319^ | Case report | Yes | Yes | Yes | Yes | Yes | Yes | No | Yes |  |  |
| Garcia-Salido 2020 ^320^ | Case series | Yes | Yes | Yes | Yes | Yes | Yes | Yes | Yes | Yes | Yes |
| DeCeano-Vivas 2020 ^321^ | Case series | Yes | Yes | Yes | Yes | Yes | Yes | Yes | Yes | Yes | Yes |
| Giesen 2020 ^322^ | Case series | Yes | Yes | Yes | Yes | Yes | Yes | Yes | Yes | Yes | N/A |
| Melgosa 2020 ^323^ | Case series | Yes | Yes | Yes | Yes | Yes | Yes | Yes | Yes | Yes | N/A |
| Cabrero-Hernandez 2020 ^324^ | Case seriess | Yes | Yes | Yes | Yes | Yes | Yes | Yes | Yes | Yes | N/A |
| deRojas 2020 ^325^ | Case series | Yes | Yes | Yes | Yes | Yes | Yes | Yes | Yes | Yes | Yes |
| VegaHernandez 2020 ^326^ | Case report | Yes | Yes | No | Yes | Yes | No | No | Yes |  |  |
| Díaz 2020 ^327^ | Case report | Yes | Yes | Yes | Yes | Yes | N/A | N/A | Yes |  |  |
| Climent 2020 ^328^ | Case report | Yes | Yes | Yes | Yes | Yes | Yes | Yes | Yes |  |  |
| Chacón-Aguilar 2020 ^329^ | Case report | Yes | Yes | Yes | Yes | Yes | Yes | N/A | Yes |  |  |
| Mondejar-Lopez 2020 ^330^ | Case Series | Yes | Yes | Yes | Yes | Yes | Yes | Yes | Yes | Yes | N/A |
| Perez-Suarez 2020 ^331^ | Case series | Yes | Yes | Yes | Yes | Yes | Yes | Yes | Yes | Unclear | N/A |
| Martinez-Castano 2020 ^332^ | Case report | Yes | Yes | Yes | Yes | Yes | Yes | Unclear | Yes |  |  |
| Poblador-Plou 2020 ^333^ | Cross sectional study | Yes | Yes | Unclear | Yes | Yes | Yes | Yes | Yes |  |  |
| FernandezColomer 2020 ^334^ | Case series | Yes | Yes | Yes | Yes | Yes | Yes | Yes | No | Yes | N/A |
| Vicent 2020 ^335^ | Case series | Yes | Yes | Yes | Yes | Yes | No | No | Yes | Yes | N/A |
| Faura 2020 ^336^ | Case series | Yes | Yes | Yes | Yes | Yes | Yes | Unclear | Yes | Yes | Yes |
| GonzalezCortes 2020 ^337^ | Case series | Yes | Yes | Yes | Yes | Yes | Yes | Yes | Yes | Yes | N/A |
| Gimeno-Costa 2020 ^338^ | Case report | Yes | Yes | Yes | Yes | Yes | Yes | Yes | Yes |  |  |
| Hildenwall 2020 ^339^ | Case series | Yes | Yes | Yes | Yes | Yes | Yes | Unclear | No | Yes | Yes |
| Nyholm 2020 ^340^ | Case report | Yes | Yes | Yes | Yes | Yes | Yes | Yes | Yes |  |  |
| Rahmanzade 2020 ^341^ | Case report |  | Yes | Yes | Yes | Yes | Yes | No | Yes |  |  |
| Andre 2020 ^342^ | Case Reports | Yes | Yes | Yes | Yes | Yes | Yes | Unclear | Yes |  |  |
| Dantonello 2020 ^343^ | Case Report | Yes | Yes | Yes | Yes | Yes | Yes | No | Yes |  |  |
| Posfay-Barbe 2020 ^344^ | Case series | Yes | Yes | Yes | Yes | Yes | Yes | Yes | Yes | Yes | Yes |
| Masmejan 2020 ^345^ | Case Series | Yes | Yes | Yes | Yes | Yes | Yes | Yes | Yes | Yes | Yes |
| Yang 2020 ^346^ | Case report | Yes | Yes | Yes | Yes | Yes | No | Yes | Yes |  |  |
| Anurathapan 2020 ^347^ | Case report | Yes | Yes | Yes | Yes | Yes | Yes | Yes | Yes |  |  |
| Moolasart 2020 ^348^ | Case report | Yes | Yes | Yes | Yes | Yes | Yes | Yes | Yes |  |  |
| Wongsawat 2020 ^349^ | Case series | Yes | Yes | Yes | Yes | Yes | No | No | Yes | Yes | N/A |
| Yasri 2020 ^350^ | Case Series | Unclear | Unclear | Unclear | Yes | No | Yes | Yes | Unclear | No | N/A |
| Yarali 2020 ^351^ | Case series | Yes | Yes | Yes | Yes | Yes | No | Yes | Yes | Yes | Yes |
| Akcabelen 2020 ^352^ | Case report | Yes | Yes | Yes | Yes | Yes | Yes | N/A | Yes |  |  |
| Kesici 2020 ^353^ | Case report | Yes | Yes | Yes | Yes | Yes | Yes | N/A | Yes |  |  |
| CuraYayla 2020 ^354^ | Case series | Yes | Yes | Yes | Yes | Yes | Yes | Yes | Yes | Yes | Yes |
| Korkmaz 2020 ^355^ | Case series | Yes | Yes | Yes | Yes | Yes | Yes | Unclear | Unclear | Yes | Yes |
| Yilmaz 2020 ^356^ | Case series | Yes | Yes | Yes | Yes | Yes | Yes | Yes | Yes | Yes | Yes |
| Sarbay 2020 ^357^ | Case report | Yes | Yes | Yes | Yes | Yes | Yes | No | Yes |  |  |
| Palabiyik 2020 ^358^ | Case series | Yes | Yes | Yes | Yes | Yes | Yes | Yes | Yes | Yes | Yes |
| Koker 2020 ^359^ | Case series | Yes | Yes | Yes | Yes | Yes | Yes | Yes | Yes | Yes | Yes |
| Yildirim 2020 ^360^ | Case report | Yes | Yes | Yes | Yes | Yes | Yes | Yes | Yes |  |  |
| Tuncer 2020 ^361^ | Case series | Yes | Yes | Yes | Yes | Yes | Yes | Yes | No | Yes | Yes |
| Onal 2020 ^362^ | Case series | Unclear | Yes | Unclear | Yes | Yes | Yes | No | No | Yes | Yes |
| Sik 2020 ^363^ | Case series | Yes | Yes | Yes | Yes | Yes | Yes | Yes | Yes | Yes | Yes |
| Gorkem 2020 ^364^ | Case report | Yes | Yes | Yes | Yes | Yes | Yes | Unclear | Yes |  |  |
| Soysal 2020 ^365^ | Case series | Yes | Yes | Yes | Yes | Yes | Yes | Yes | No | Yes | Yes |
| Kanburoglu 2020 ^366^ | Case series | Yes | Yes | Yes | Yes | Yes | Yes | Yes | Yes | Yes | Yes |
| ElDannan 2020 ^367^ | Case series | Yes | Yes | Yes | Yes | Yes | Yes | Yes | Yes | Yes | N/A |
| Kirenga 2020 ^368^ | Case Series | Yes | Yes | Yes | Yes | Yes | Yes | Yes | Yes | Yes | Yes |
| Swann 2020 ^369^ | Case series | Yes | Yes | Yes | Yes | Yes | Yes | Yes | Yes | Yes | Yes |
| Gale 2020 ^370^ | Case series | Yes | Yes | Yes | Yes | Yes | Yes | Yes | Yes | Yes | Yes |
| Barsoum 2020 ^371^ | Case report | Yes | Yes | Yes | Yes | Yes | Yes | Yes | Yes |  |  |
| Patel 2020 ^372^ | Case report | Yes | Yes | Yes | Yes | Yes | Yes | Yes | Yes |  |  |
| Stokes 2020 ^373^ | Case series | Yes | Yes | Yes | Yes | Yes | Yes | Yes | Yes | Yes | N/A |
| Simpson 2020 ^374^ | Case series | Yes | Yes | Yes | Yes | Yes | Yes | Yes | Yes | Yes | N/A |
| Samies 2020 ^375^ | Case report | Yes | Yes | Yes | Yes | Yes | Yes | Yes | Yes |  |  |
| Wahlster 2020 ^376^ | Case report | Yes | Yes | Yes | Yes | Yes | Yes | Unclear | Yes |  |  |
| Diercks 2020 ^377^ | Case report | Yes | Yes | Yes | Yes | Yes | Yes | Yes | Yes |  |  |
| Shaw 2020 ^378^ | Case report | Yes | Yes | Yes | Yes | Yes | Yes | Yes | Yes |  |  |
| Wardell 2020 ^379^ | Case series | Yes | Yes | Yes | Yes | Yes | Yes | Yes | Yes | Yes | N/A |
| Agha 2020 ^380^ | Case series | Yes | Yes | No | Yes | Yes | Yes | Yes | Yes | Yes | Yes |
| Mithal 2020 ^381^ | Case series | Yes | Yes | Yes | Yes | Yes | Unclear | Yes | Yes | Yes | Yes |
| Rossoff 2020 ^382^ | Case series | Yes | Yes | Yes | Yes | Yes | Yes | Yes | Yes | Yes | N/A |
| Mannheim 2020 ^383^ | Case series | Unclear | Yes | Yes | Yes | Yes | Yes | Unclear | Yes | Yes | Yes |
| Jones 2020 ^384^ | Case report | Yes | Yes | Yes | Yes | Yes | Yes | Yes | Yes |  |  |
| Danley 2020 ^385^ | Case report | Yes | Yes | Yes | Yes | Yes | Yes | Unclear | Yes |  |  |
| White 2020 ^386^ | Case series | Yes | Yes | Yes | Yes | Yes | Yes | Yes | Yes | Yes | N/A |
| DeBiasi 2020 ^387^ | Case series | Unclear | Yes | Yes | Yes | Yes | Yes | Yes | Yes | Yes | Yes |
| Patek 2020 ^388^ | Case report | Yes | Yes | Yes | Yes | Yes | Yes | No | Yes |  |  |
| Bush 2020 ^389^ | Case report | Yes | Yes | Yes | Yes | Yes | Yes | Yes | Yes |  |  |
| Coronado Munoz 2020 ^390^ | Case report | Yes | Yes | Yes | Yes | Yes | Yes | Yes | Yes |  |  |
| Bhumbra 2020 ^391^ | Case series | Unclear | Yes | Yes | Yes | Yes | Unclear | Yes | Yes | Yes | Yes |
| Severance 2020 ^392^ | Case report | Yes | Yes | Yes | Yes | Yes | Yes | Yes | Yes |  |  |
| Mehta 2020 ^393^ | Case report | Yes | Yes | Yes | Yes | Yes | Yes | Yes | Yes |  |  |
| Precit 2020 ^394^ | Case report | Yes | Yes | Yes | Yes | Yes | Yes | Yes | Yes |  |  |
| Dumpa 2020 ^395^ | Case report | Yes | Yes | Yes | Yes | Yes | Yes | Yes | Yes |  |  |
| Pierce-Williams 2020 ^396^ | Case series | Yes | Yes | Yes | Yes | Yes | Yes | Yes | Yes | Yes | Yes |
| Lara 2020 ^397^ | Case report | Yes | Yes | Yes | Yes | Yes | Yes | Yes | Yes |  |  |
| Craver 2020 ^398^ | Case report | Yes | Yes | Yes | Yes | Yes | Yes | Yes | Yes |  |  |
| Paret 2020 ^399^ | Case series | Yes | Yes | Yes | Unclear | Yes | Yes | Yes | Yes | Yes | N/A |
| Acker 2020 ^400^ | Case series | Yes | Yes | Yes | Yes | Yes | Yes | Yes | Unclear | Yes | Yes |
| Kalyanaraman 2020 ^401^ | Case report | Yes | Yes | Yes | Yes | Yes | Yes | Yes | Yes |  |  |
| Lee 2020 ^402^ | Case series | Yes | Yes | Yes | Yes | Yes | Yes | Yes | Yes | Yes | N/A |
| Lagana 2020 ^403^ | Case report | Yes | Yes | Yes | Yes | Yes | Unclear | No | Yes |  |  |
| Dugue 2020 ^404^ | Case report | Yes | Yes | Yes | Yes | Yes | Yes | N/A | Yes |  |  |
| Derespina 2020 ^405^ | Case series | Yes | Yes | Yes | Yes | Yes | No | Yes | Yes | Yes | Yes |
| Feld 2020 ^406^ | Case series | Yes | Yes | Yes | Yes | Yes | Yes | Yes | Yes | No | N/A |
| Enner 2020 ^407^ | Case report | Yes | Yes | Yes | Yes | Yes | Yes | Yes | Yes |  |  |
| Kainth 2020 ^408^ | Case series | Yes | Yes | Yes | Yes | Yes | No | Yes | Yes | Yes | Yes |
| Gefen 2020 ^409^ | Case report | Yes | Yes | Yes | Yes | Yes | Yes | Yes | Yes |  |  |
| Perez 2020 ^410^ | Case series | Yes | Yes | Yes | Yes | Yes | Yes | Yes | Yes | Yes | N/A |
| Appiah-Kubi 2020 ^411^ | Case series | Yes | Yes | Yes | Yes | Yes | Yes | Yes | Yes | Yes | N/A |
| Lewis 2020 ^412^ | Case report | Yes | Yes | Yes | Yes | Yes | Yes | No | Yes |  |  |
| Heinz 2020 ^413^ | Case report | Yes | Yes | Yes | Yes | Yes | Yes | Yes | Yes |  |  |
| Krishnan 2020 ^414^ | Case series | Yes | Yes | Yes | Yes | Yes | Yes | No | Yes | Yes | N/A |
| Kihira 2020 ^415^ | Case report | Yes | Yes | Yes | Yes | Yes | No | Yes | Yes |  |  |
| Chao 2020 ^416^ | Case series | Yes | Yes | Yes | Yes | Yes | No | Yes | Yes | Yes | Yes |
| Almassi 2020 ^417^ | Case report | Yes | Yes | Yes | Yes | Yes | Yes | Yes | Yes |  |  |
| Gampel 2020 ^418^ | Case series | Yes | Yes | Yes | Yes | Yes | Yes | Yes | Yes | Yes | Yes |
| Choi 2020 ^419^ | Case report | Yes | Yes | Yes | Yes | Yes | Yes | Yes | Yes |  |  |
| Trogen 2020 ^420^ | Case report | Yes | Yes | Yes | Yes | Yes | Yes | Yes | Yes |  |  |
| Khoury 2020 ^421^ | Case series | Yes | Yes | Yes | Yes | Yes | Yes | No | No | Yes | Yes |
| McLaren 2020 ^422^ | Case series | Yes | Yes | Yes | Yes | Yes | Yes | No | No | Yes | N/A |
| Salik 2020 ^423^ | Case report | Yes | Yes | No | Yes | Yes | Yes | Yes | Yes |  |  |
| McAbee 2020 ^424^ | Case report | Yes | Yes | Yes | Yes | No | No | N/A | Yes |  |  |
| Farley 2020 ^425^ | Case report | Yes | Yes | Yes | Yes | Yes | Yes | Yes | Yes |  |  |
| SeeTsao 2020 ^426^ | Case report | Yes | Yes | Yes | Yes | Yes | Yes | Yes | Yes |  |  |
| Otto 2020 ^427^ | Case series | Yes | Yes | Yes | Yes | Yes | Yes | Yes | Yes | Yes | Yes |
| Kan 2020 ^428^ | Case report | Yes | Yes | Yes | Yes | Yes | Yes | Yes | Yes |  |  |
| Robbins 2020 ^429^ | Case report | Yes | Yes | Yes | Yes | Yes | Yes | N/A | Yes |  |  |
| Russell 2020 ^430^ | Case report | Yes | Yes | Yes | Yes | Yes | Yes | Yes | Yes |  |  |
| Bixler 2020 ^431^ | Cross sectional study | Yes | Yes | Yes | Yes | No | No | Yes | Yes |  |  |
| Woodworth 2020 ^432^ | Case Series | Yes | Yes | Yes | Yes | Yes | Yes | Yes | Yes | Yes | Yes |
| Turbin 2020 ^433^ | Case series | Yes | Yes | Yes | Yes | Yes | Yes | Yes | Yes | Yes | N/A |
| Alloway 2020 ^434^ | Case report | Yes | Yes | Yes | Yes | Yes | Yes | Yes | Yes |  |  |
| Sisman 2020 ^435^ | Case report | Yes | Yes | Yes | Yes | Yes | Yes | Yes | Yes |  |  |
| Stokes 2020 ^436^ | Cross sectional study | Yes | Yes | Yes | Yes | Yes | Yes | Yes | Yes |  |  |
| Team CDC COVID 2020 ^437^ | Case series | Yes | Unclear | Unclear | Yes | Yes | Yes | No | No | Yes | Yes |
| Shekerdemian 2020 ^438^ | Case series | Yes | Yes | Yes | Yes | Yes | No | Yes | Yes |  |  |
| Sachdeva 2020 ^439^ | Case series | Yes | Yes | Yes | Yes | Yes | Yes | Unclear | Yes | Yes | Yes |
| Kim 2020 ^440^ | Case series | Yes | Yes | Yes | Yes | Yes | Yes | No | No | Yes | N/A |
| Le 2020 ^441^ | Case report | Yes | Yes | Yes | Yes | Yes | Unclear | Yes | Yes |  |  |
| Nguyen 2020 ^442^ | Case Series | Yes | Yes | Yes | Yes | Yes | Yes | Yes | Yes | Yes | Yes |
| Al-Waleedi 2020 ^443^ | Cross sectional study | Yes | Yes | Yes | Yes | No | No | Yes | Yes |  |  |

Checklist scoring for risk of bias assessment were conducted using a checklist of Critical Appraisal Tools of Joanna Briggs Institute.

References are listed in S1 Table.

Abbreviations: Q, question

**Reference**

1. Joanna Briggs Institute. Critical Appraisal Tools. https://joannabriggs.org/ebp/critical_appraisal_tools. Accessed Apr 30, 2020.
