# Supplementary material for: The differential impact of pediatric COVID-19 between high-income countries and low- and middle-income countries: A systematic review of fatality and ICU admission in children worldwide
Source: PLoS One. 2021 Jan 29;16(1):e0246326. doi: 10.1371/journal.pone.0246326 (PMC7845974; doi:10.1371/journal.pone.0246326)
Supplement: S4 Table — (DOCX) [file pone.0246326.s009.docx]

**S4 Table Age-specific fatality and ICU admission (per 1,000,000 children), Case fatality rate and ICU admission rate**

|  | **Fatality (0-19y)** | **Fatality (<1y)** | **Fatality (1-4y)** | **Fatality (5-9y)** | **Fatality (10-14y)** | **Fatality (15-19y)** |
| --- | --- | --- | --- | --- | --- | --- |
| Global | 2.53 (3,713/1,466,708) | 10.03 (393/39,184) | 1.64 (242/147,256) | 0.92 (180/196,676) | 1.13 (213/187,721) | 2.70 (494/1582,890) |
| HIC | 1.32 (319/242,496) | 5.39 (36/6,684) | 0.59 (13/22,217) | 0.48 (14/29,231) | 0.76 (20/26,149) | 2.46 (60/24,393) |
| LMIC | 2.77 (3,426/1,224,212) | 10.98 (357/32,500) | 1.83 (229/125,040) | 0.99 (166/167,445) | 1.19 (193/161,572) | 2.74 (434/158,497) |
| Upper MIC | 3.83 (2,367/617,795) | 12.13 (354/29,175) | 2.05 (229111,795) | 1.05 (146/138,633) | 1.20 (163/135,356) | 2.88 (387/134,576) |
| Lower MIC | 2.01 (979/487,644) | 0.00 (0/2,239) | 0.00 (0/9,174) | 0.74 (18/24,252) | 1.26 (28/22,163) | 2.30 (47/20,470) |
| LIC | 0.40 (48/118,773) | 2.76 (3/1,086) | 0.00 (0/4,071) | 0.44 (2/4,561) | 0.49 (2/4,054) | 0.00 (0/3,451) |
|  | **ICU admission (0-19y)** | **ICU admission (<1y)** | **ICU admission (1-4y)** | **ICU admission (5-9y)** | **ICU admission (10-14y)** | **ICU admission (15-19y)** |
| Global | 4.99 (2,644/530,039) | 16.84 (356/21,137) | 1.41 (121/86,313) | 0.73 (81/110,775) | 0.97 (105/108,082) | 2.78 (288/103,475) |
| HIC | 18.80 (2,020/107,440) | 42.72 (36/843) | 6.74 (10/1,483) | 0.00 (0/2,535) | 0.00 (0/2,551) | 0.00 (0/55) |
| LMIC | 1.48 (624/422,654) | 15.90 (320/20,294) | 1.31 (111/84,830) | 0.75 (81/107,240) | 0.99 (105/105,531) | 2.74 (37/103,420) |
| Upper MIC | 1.49 (624/419,373) | 15.90 (320/20,130) | 1.32 (111/84,197) | 0.75 (81/108,470) | 1.00 (105/104,473) | 2.88 (37/102,711) |
| Lower MIC | 0 (0/3,281) | 0.00 (0/164) | 0.00 (0/633) | 0.00 (0/770) | 0.00 (0/758) | 0.00 (0/709) |
| LIC | N/A | N/A | N/A | N/A | N/A | N/A |
|  | **CFR (0-19y)** | **CFR (<1y)** | **CFR (1-4y)** | **CFR (5-9y)** | **CFR (10-14y)** | **CFR (15-19y)** |
| Global | 0.06% (2,061/3,379,049) | 0.58% (176/30,214) | 0.18% (99/55,704) | 0.08% (98/121,724) | 0.06% (111/172,287) | 0.14% (242/179,032) |
| HIC | 0.013% (310/2,653,444) | 0.07% (12/17,584) | 0.004% (1/24,490) | 0.002% (1/66,602) | 0.003% (2/98,507) | 0.004% (2/49,267) |
| LMIC | 0.24% (1,751/725,605) | 1.30% (164/12,630) | 0.31% (98/31,214) | 0.23% (97/55,122) | 0.15% (108/73,780) | 0.19% (240/129,765) |
| Upper MIC | 0.15% (725/482,441) | 1.33% (161/12,103) | 0.33% (98/29,714) | 0.17% (78/46,803) | 0.12% (78/64,319) | 0.18% (199/114,018) |
| Lower MIC | 0.43% (979/225,979) | 0.00% (0/380) | 0.00% (0/1022) | 0.22% (17/7,866) | 0.31% (28/8,925) | 0.27% (41/15,048) |
| LIC | 0.27% (47/17,185) | 2.04% (3/147) | 0.00% (0/478) | 0.44% (2/453) | 0.37% (2/536) | 0.00% (0/699) |
|  | **ICU admission rate (0-19y)** | **ICU admission rate (<1y)** | **ICU admission rate (1-4y)** | **ICU admission rate (5-9y)** | **ICU admission rate (10-14y)** | **ICU admission rate (15-19y)** |
| Global | 0.15% (2,644/1,738,306) | 1.41% (361/25,576) | 0.26% (121/47,028) | 0.25% (83/33,074) | 0.22% (11/50,313) | 0.13% (48/37,254) |
| HIC | 0.13% (2,020/1,581,263) | 0.85% (41/17,403) | 0.04% (10/24,529) | 0.52% (2/388) | 0.88% (6/681) | 0.37% (3/813) |
| LMIC | 0.40% (624/157,043) | 3.92% (320/8,173) | 0.49% (111/22,499) | 0.25% (81/32,686) | 0.21% (105/49,632) | 0.12% (45/36,441) |
| Upper MIC | 0.40% (624/157,042) | 3.92% (320/8,173) | 0.49% (111/22,499) | 0.25% (81/32,684) | 0.21% (105/49,625) | 0.12% (45/36,390) |
| Lower MIC | 0.00% (0/1) | N/A | N/A | 0.00% (0/2) | 0.00% (0/7) | 0.00% (0/51) |
| LIC | N/A | N/A | N/A | N/A | N/A | N/A |

Abbreviations: ICU, intensive care unit; CFR, case fatality rate; N/A, not available

Fatality and ICU admission was presented as per 1,000,000 children. The number of children (=denominator of incidence) was presented as per 1,000.

Age-specific national data with up to one year difference of age buckets were included. For example, age-specific national data reporting outcomes of 1-5 years and 10-15 years were included in our calculation of 1-4 years and 10-14 years.
