# Supplementary material for: The differential impact of pediatric COVID-19 between high-income countries and low- and middle-income countries: A systematic review of fatality and ICU admission in children worldwide
Source: PLoS One. 2021 Jan 29;16(1):e0246326. doi: 10.1371/journal.pone.0246326 (PMC7845974; doi:10.1371/journal.pone.0246326)
Supplement: S5 Table — (DOCX) [file pone.0246326.s010.docx]

**S5 Table. ICU cases in children aged 0-19 years with confirmed SARS-CoV-2 infection cases who have known respiratory or circulatory support outcome**

|  | **The number of ICU admissions**  **.** | **The number of NICU admissions** |  | **Maximal respiratory support** | | | | | | | | **Vasopressor** |
| --- | --- | --- | --- | --- | --- | --- | --- | --- | --- | --- | --- | --- |
|  |  |  | None | | | Oxygen only | | HFNC/HFOV | Non-invasive ventilation | Invasive ventilation | ECMO |  |
| Belgium | 1 | 1 | 0 | | 0 | | | 0 | 1 | 0 | 0 | 0 |
| Brazil | 69 | N/A | 0 | | | 33 | N/A | | 5 | 14 | N/A | 0 |
| Canada ^a^ | 2 | N/A | 0 | | | | | 1 | 0 | 1 | 0 | N/A |
| China **^b^** | 21 | 6 | 6 | | | 5 | | 0 | 3 | 7 | 0 | 2 |
| France | 25 | 1 | 3 | | | 2 | | 1 | 7 | 12 | 1 | 4 |
| Germany **^c^** | 17 | N/A | N/A | | | N/A | | N/A | 7 | N/A | N/A | N/A |
| Greece | 1 | 0 | 0 | | | 0 | | 0 | 0 | 1 | 0 | 0 |
| Grenada | 1 | 1 | 1 | | | 0 | | 0 | 0 | 0 | 0 | 0 |
| India **^d^** | 12 | 3 | 4 | | | | | 2 | 2 | 4 | 0 | 0 |
| Indonesia | 1 | 0 | 0 | | | 0 | | 0 | 0 | 1 | 0 | 0 |
| Iran | 23 | 10 | 2 | | | 1 | | 0 | 2 | 18 | 0 | 1 |
| Italy **^d^** | 18 | 1 | N/A | | | 4 | | 3 | 1 | 3 | 0 | 0 |
| Japan | 1 | 0 | 0 | | | 0 | | 0 | 0 | 1 | 0 | 0 |
| Kuwait | 3 | 2 | 1 | | | 1 | | 0 | 0 | 1 | 0 | 0 |
| Luxembourg | 1 | 0 | 0 | | | 0 | | 0 | 0 | 1 | 0 | 1 |
| Mexico **^c^** | 428 | N/A | N/A | | | N/A | | N/A | N/A | 190 | N/A | N/A |
| Morocco | 1 | 0 | 0 | | | 0 | | 0 | 0 | 1 | 0 | N/A |
| Oman | 3 | 3 | 3 | | | 0 | | 0 | 0 | 0 | 0 | 0 |
| Peru | 2 | 1 | 0 | | | 0 | | 0 | 1 | 1 | 0 | 0 |
| Portugal | 2 | 1 | 0 | | | 1 | | 0 | 0 | 1 | 0 | 0 |
| Qatar | 1 | 0 | 1 | | | 0 | | 0 | 0 | 0 | 0 | 0 |
| Romania | 1 | 0 | 0 | | | 0 | | 0 | 0 | 1 | 0 | 0 |
| Russia | 2 | 0 | 0 | | | 2 | | 0 | 0 | 0 | 0 | 0 |
| Saudi Arabia | 4 | 1 | 1 | | | 1 | | 0 | 0 | 2 | 0 | 0 |
| Spain | 29 | 9 | 3 | | | 5 | | 6 | 5 | 10 | 3 | 6 |
| Sweden | 1 | 0 | 0 | | | 0 | | 0 | 0 | 1 | 0 | 0 |
| Switzerland | 2 | 0 | 0 | | | 0 | | 1 | 1 | 0 | 0 | 0 |
| Turkey **^c^** | 50 | 27 | 5 | | | 18 | | 1 | 14 | 7 | 2 | 1 |
| UK **^c^** | 116 | 20 | N/A | | | N/A | | N/A | 57 | 58 | 2 | N/A |
| USA **^a^** | 1086 | 9 | 573 | | | | | 160 | 95 | 242 | 16 | N/A |
| Total | 1924 | 96 | 680 | | | | | 175 | 201 | 578 | 24 | N/A |

Abbreviations: ICU, intensive care unit; NICU, neonatal intensive care unit; HFNC, high flow nasal cannula; HFOV, high frequency oscillatory ventilation; ECMO, extracorporeal membrane oxygenation; N/A, not available

The number of ICU admission also includes the number of NICU admission

Non-invasive ventilation includes continuous positive airway pressure (CPAP) and bilevel positive airway pressure (BiPAP) support.

**^a^** Maximal respiratory support data did not differentiate no respiratory support and oxygen support only.

**^b^** Two cases reported no respiratory support may have had oxygen therapy

**^c^** Only the number of patients with invasive ventilation among ICU cases was available.

**^d^** The detailed supports of some cases were unknown
