# Supplementary material for: The differential impact of pediatric COVID-19 between high-income countries and low- and middle-income countries: A systematic review of fatality and ICU admission in children worldwide
Source: PLoS One. 2021 Jan 29;16(1):e0246326. doi: 10.1371/journal.pone.0246326 (PMC7845974; doi:10.1371/journal.pone.0246326)
Supplement: S6 Table — (DOCX) [file pone.0246326.s011.docx]

**S6. Table. Sensitivity analysis using alternative data source**

|  | **Global pediatric fatality incidence** | **Pediatric fatality incidence in HICs** | **Pediatric fatality incidence in LMICs** | ***p*** | **Global pediatric CFR** | **Pediatric CFR in HICs** | **Pediatric CFR in LMICs** | ***p*** |
| --- | --- | --- | --- | --- | --- | --- | --- | --- |
| Base case **^a^** | 2.53 | 1.32 | 2.77 | <0.001 | 0.06% | 0.01% | 0.24% | <0.001 |
| Bolivia **^b^** | 2.53 | 1.32 | 2.77 | <0.001 | 0.06% | 0.01% | 0.24% | <0.001 |
| Burkina Faso **^c^** | 2.51 | 1.32 | 2.75 | <0.001 | 0.06% | 0.01% | 0.24% | <0.001 |
| Cyprus **^c^** | 2.52 | 1.31 | 2.77 | <0.001 | 0.06% | 0.01% | 0.24% | <0.001 |
| Guinea **^c^** | 2.52 | 1.32 | 2.76 | <0.001 | 0.06% | 0.01% | 0.24% | <0.001 |
| India **^b^** | 2.44 | 1.32 | 2.65 | <0.001 | 0.06% | 0.01% | 0.23% | <0.001 |
| Iran **^d^** | 2.50 | 1.32 | 2.73 | <0.001 | 0.06% | 0.01% | 0.24% | <0.001 |
| Iraq **^d^** | 2.50 | 1.32 | 2.73 | <0.001 | 0.06% | 0.01% | 0.24% | <0.001 |
| Jordan **^d^** | 2.52 | 1.32 | 2.76 | <0.001 | 0.06% | 0.01% | 0.24% | <0.001 |
| Kazakhstan **^c^** | 2.53 | 1.32 | 2.76 | <0.001 | 0.06% | 0.01% | 0.24% | <0.001 |
| Kenya **^c^** | 2.49 | 1.32 | 2.72 | <0.001 | 0.06% | 0.01% | 0.24% | <0.001 |
| Kuwait **^c^** | 2.53 | 1.31 | 2.77 | <0.001 | 0.06% | 0.01% | 0.24% | <0.001 |
| Morocco **^d^** | 2.51 | 1.32 | 2.74 | <0.001 | 0.06% | 0.01% | 0.24% | <0.001 |
| Niger **^d^** | 2.51 | 1.32 | 2.74 | <0.001 | 0.06% | 0.01% | 0.24% | <0.001 |
| Norway **^c^** | 2.53 | 1.31 | 2.77 | <0.001 | 0.06% | 0.01% | 0.24% | <0.001 |
| Oman **^c^** | 2.53 | 1.31 | 2.77 | <0.001 | 0.06% | 0.01% | 0.24% | <0.001 |
| Pakistan **^c^** | 2.38 | 1.32 | 2.57 | <0.001 | 0.06% | 0.01% | 0.24% | <0.001 |
| Qatar **^c^** | 2.53 | 1.31 | 2.77 | <0.001 | 0.06% | 0.01% | 0.24% | <0.001 |
| Russia **^c^** | 2.48 | 1.32 | 2.71 | <0.001 | 0.06% | 0.01% | 0.24% | <0.001 |
| Saudi Arabia **^d^** | 2.51 | 1.26 | 2.77 | <0.001 | 0.06% | 0.01% | 0.24% | <0.001 |
| Trinidad Tobago **^c^** | 2.53 | 1.32 | 2.77 | <0.001 | 0.06% | 0.01% | 0.24% | <0.001 |
| UAE **^d^** | 2.53 | 1.31 | 2.77 | <0.001 | 0.06% | 0.01% | 0.24% | <0.001 |
| Yemen **^d^** | 2.51 | 1.32 | 2.74 | <0.001 | 0.06% | 0.01% | 0.24% | <0.001 |
| All national reports **^e^** | 2.26 | 1.29 | 2.43 | <0.001 | 0.06% | 0.01% | 0.24% | <0.001 |

Incidence was presented as per 1,000,000 children.

Abbreviations: CFR, case fatality rate; HIC, high-income country; LMIC, low- and middle-income country

*p*-values were calculated by fatality incidence/1,000,000 children or CFR between HICs and LMICs

**^a^** In the base case, the fatality incidence and CFR were calculated from the national data in Table 1

**^b^** Only subnational data were identified (La Paz city from Bolivia, Tamil Nadu and Andhra Pradesh regions from India). Because of the lack of nationally representative data, these countries were not included into the base case analysis, but were evaluated in the sensitivity analysis

**^c^** The nationwide data were reported more than 2 months before the date of final search (Dec 7, 2020), these countries were grouped CDC COVID-19 level 2 or more (moderate risk or more). The data were not included into the base case analysis, but were evaluated in the sensitivity analysis.

**^d^** Only case report or case series from hospitals were identified in the country. Because of the lack of nationally representative data, these countries were not included into the base case analysis, but were evaluated in the sensitivity analysis

**^e^** All nationwide data excluded from the base case analysis were included.
