# Supplementary material for: The differential impact of pediatric COVID-19 between high-income countries and low- and middle-income countries: A systematic review of fatality and ICU admission in children worldwide
Source: PLoS One. 2021 Jan 29;16(1):e0246326. doi: 10.1371/journal.pone.0246326 (PMC7845974; doi:10.1371/journal.pone.0246326)
Supplement: Attachment — Submitted filename: rsponse to reviewer.docx [file pone.0246326.s012.docx]

This manuscript reflects an enormous amount of work and will provide an important contribution to current knowledge about pediatric morbidity and mortality worldwide. Please address all the comments by the two reviewers, who spent considerable time and effort reviewing the paper.

In addition, in the discussion section, please mention the geographic distribution of the manuscripts and data cited in the review, and highlight the fact, which is clear from the tables, that much of the data from LMICs was from certain regions, such as the Americas, and other regions, like Sub-Saharan Africa and Asia, contributed very little data.

Finally, you mentioned your willingness to update your search to cover the period that has elapsed from the time of the last search until now. Because new data are constantly emerging in this pandemic, I believe updating the search to include the latest manuscripts and reports would make the findings of this review even more timely and relevant. I would encourage you to do this if you and your team are still willing and able.

Thank you very much for kindly recognizing our work. We are sincerely thankful for editor and reviewers for time and effort to review our manuscript as well as very thoughtful comments in this unprecedented time. We updated our search (search 3) starting on Dec 7^th^ based on your suggestion. After discussion and agreement with editor and our author team, this update search had two new changes compared to our previous searches.

1. searching term: (COVID-19 related words) AND (pediatrics words) AND (outcome related words) for this 3^rd^ search, compared to

(COVID-19 related words) AND (pediatrics words OR outcome related words) for our first term search and  
(COVID-19 related words) AND (pediatrics words) for our second term search (described in Supplemental file 1)

2. Based on our previous two review experiences with more than 10,000 articles reviewed, academic articles from countries with comprehensive national reports have never contributed to our main outcome (national fatality and ICU admissions) because the national reports have been more update and comprehensive regarding our main outcomes. (These articles were 100% deemed "overlapped”). Therefore, for academic article search in this new update, we only focused on articles from countries without comprehensive national reports. However, we included articles reporting nationwide subgroup outcomes (neonatal, age-specific or detailed ICU outcomes), even if the reports were from countries with comprehensive national reports to capture main (national fatality and ICU admissions) and subgroup (neonatal, age-specific or detailed ICU outcomes) from all countries as of the date of update search. (Line 125-128)

All other condition of the new update was exactly same to the 2^nd^ term search. The details of the new update search were provided in S1 file (supporting methods: page 1 and 14-17). By these new changes, we could manage to update our result for approximately 4 weeks (the other 2 searches took about 2 months for each time to complete our review). We do not believe this new change would have had any impact on our ability to make inferences about our outcomes.

Added the following sentence in discussion “There is a geographical disparity about countries which contributed to our study. While many American and European countries reported nationwide data in children, the data from Africa and Middle East were limited”

Reviewer #1: This is an extremely ambitious and comprehensive analysis of the published data regarding mortality and ICU admission for PCR-confirmed SARS-Cov2 infection in children. The paper required a large amount of work and cooperation among the coauthors and as such represents an admirable effort. That said, the pandemic is still in full swing, many countries currently experiencing their second waves as the Northern hemisphere moves into winter; thus it must be considered an interim snapshot of the first 8 months of the pandemic only.

General comments:
There is a problem with both the terminology of the outcomes addressed and their validity. The outcomes addressed in this study include case fatality rates (CFR), ICU admission rates and "fatality prevalence". The latter is not an acceptable term in infectious disease epidemiology or general epidemiology for that matter. Death is an instantaneous event and therefore cannot be prevalent. It can only be incident. "Mortality rate" might be an acceptable alternative, "deaths from COVID per million" could also be used. The "period prevalence" of death is not pertinent, for similar reasons.

We avoided the term “prevalence” in our revision and used the term “deaths or ICU admissions per million children”.

In fact, in the absence of person-time denominators, the number of days since the declaration of the pandemic, or some other benchmark date ( eg Dec 31, 2019) should be taken into account when reporting these rates since they will be highly influenced by date of reporting. CFR, as the authors note, is highly dependent on testing policies and will inevitably rise where testing is scarce and limited to symptomatic or severely ill children, and therefore will be elevated in resource-scarce settings. The authors should consider "normalizing" these rates by tests per million in the populations studied.

Thank you for pointing this out. To minimize the influence of these factors on our outcomes, we added the followings;

-For pediatric population (=the denominator for deaths or ICU incidence), we used UN data for estimation of national population by each age on July 1, 2020 for ALL countries. (Line 171-172)

-For national reports or articles reported 2 months or more before the date of search, we evaluated the data as follows; Data from countries with low transmission risk since the date of report (CDC COVID-19 Level 1) were included in our primary calculation. Data from other countries (CDC COVID-19 Level 2-4) were not included in our primary calculation, but they were evaluated in our sensitivity analysis. (Line 199-203)

Regarding ICU admission rates, this is clearly not a fair comparison between HIC and LMIC, many of which probably have no pediatric ICU care. Access to this type of care should be mentioned in the introduction. The dichotomization of the data into HIC and LMIC is questionable given the more detailed information presented in Figures 3A and B.
We agree that the comparison of ICU admission between HIC and LMIC does not reflect the severity of disease. Rather, the discrepancy of fatality and ICU admissions between HIC and LMIC (more deaths in LMIC, but more ICU admissions in HIC) reflects the issue of access to intensive care for children in LMIC. In the discussion, we emphasized that the comparison of ICU rates is more to indicate the difference of access to ICU care rather than to indicate the difference of disease severity (Line 282-285)

Access to ICU care was added in introduction.

We agree that there is a large variation of fatality and ICU admission outcome among lower income countries (upper MIC, lower MIC and LIC). For the variation of fatality, the discussion was added at the end of the first paragraph in discussion. For ICU outcome, this is more for the lack of data from lower MIC and LIC (supplemental table 4), and because we agree that presenting ICU data by figures is a bit confusing. ICU figures were removed. Also, the scarcity of ICU data from lower income countries was described in limitation section of discussion.

Specific Comments
Abstract – a definition of pediatric cases (up to 19 years) should appear in the abstract). The percentage of deaths in HIC and LMICs should also be accompanied by percent of the world's children living in HICs and LMICs respectively. For instance, according to the World Bank 16% of the population in HIC and 27% in LMIC are children under the age of 14 –<https://data.worldbank.org/indicator/SP.POP.0014.TO.ZS?name_desc=true>. Do we know the total numbers up to age 19? The proportion dying of COVID-10 should also be presented in the context of the total proportion of children in these countries. The statement "The highest fatality prevalence and CFR were in infants < 1 year old (9.58/1,000,000 children and 0.55%, respectively) does not take into account HIC and LMIC – this comparison should be added.

The definition of pediatric cases (0-19 years) and percent of the world's pediatric population were added in abstract. The vast majority of included countries report pediatric COVID-19 data for 0-19 years. For these countries, we used 0-19 years population to calculate our main outcomes. A few countries report pediatric COVID-19 data only for 0-14 years or 0-20 years. For these countries, the population of the corresponding age groups (0-14 or 0-20 years) were used to calculate our outcomes.

The infantile fatality data in HIC and LMIC were added in abstract

Introduction- the authors acknowledge that previous studies have shown that death from COVID-19 in children is rare. They should stress what this study adds. Please mention ICU availability in LMICs to justify the pertinence of this comparison.

Introduction was modified (Line 65-69). The sentence about ICU availability in LMIC was added. (Line 74-76)

Methods:
Country reports are available to end of August, while published reports end as of August 10. Given delays in publishing, this means that published reports are highly skewed to the early months of the pandemic. In a revision, these should be updated. It is not clear how data overlap between country reports and published manuscripts was handled. Many case series are included – these may be outbreak investigations in schools, clinical case series – how were data extracted from these studies concerning the study outcomes? How do we know that there are no duplicate reports?
For both national reports and published articles, our new updated was conducted on Dec 7 based on your suggestion. For national reports, it was impossible to complete all extractions in a single day, and it took a few days from December 7 (because some countries just report every individual case in separate news letters, and other countries report individual cases as a line list. Our reviewers counted all of these individual cases to extract the national data.) But we believe that this several days interval in national reports does not affect our overall outcomes.

Our exhaustive and elaborate overlap checking processes are also explained in supplemental file 1 (pp18-19) with a few examples (School outbreak is example 4). We are exclusive to avoid double-counting a case with any possibility of overlapping. Although we may have excluded articles which were not actually overlapped with another article (due to their unclear information, and then we could not consider them as completely distinct from another article), we believe that the avoidance of overcount is more important.

Results:
Lines 213-215 – in order to interpret these results, as mentioned, we need to know what proportion of children live in LMICS
The following sentence was added. “while 16.5% and 83.5% of pediatric population from all included countries were from HIC and LMIC respectively”

Figure 3A shows variation by country- there is a clear excess of deaths in South America in countries around the equator. This is not addressed in the text. Supplement S2 B – there is no HIC in the graph although the abbreviation is found in the legend.

The following sentence was added “Fig 2 shows the variation of deaths/1,000,000 children by country. There is an excess of deaths in Middle and South American countries.”

We agree that the comparison of ICU admission between HIC vs LMIC needs to be interpreted with cautions. This is because few lower MIC and LIC report ICU admission data compared to upper MIC and HIC (this is more apparent in updated data), while a relatively good number of countries from all 4 groups report fatality data. The phrase “although the ICU admission data from lower MIC and LIC were scarce” was added in the result section. Because presenting ICU admission by country income can be confusing given the lack of data from lower income countries, as shown in Supplemental Table S4, Figure 3B and Supplemental Figure S2B were removed

Figure Legends for age-specific data was corrected.

Figure 3Bthe picture examining LIC LMIC HMIC and HIC is very different from the dichotomous analysis presented in the abstract and results.

Because few countries from lower MIC and LIC report ICU outcome, we agree that presenting ICU outcomes by graph of 4 different income categories can be confusing. Figure 3B and Figure S2B were removed. We stated that ``the ICU admission data from lower MIC and LIC were scarce (Supplemental Table 4)``(Line 232)

Discussion line 282 – there are many reasons for a higher CFR besides less testing in LICs/ Is it possible that baseline characteristics of children admitted for COVID-19 differ between HIC and LIMIC? Could malnutrition, control of chronic conditions be playing a role in case fatality. This is important since comorbidity has been identified as a major risk factor for death in pediatric cases in HICs, according to multiple reports. Lines 283-300- this is a very long and repetitive explanation of the same point. Discussion can be shortened and tightened up.

We agree that chronic condition and especially malnutrition in LMIC are important factors for severe. Though our study was not designed to investigate these conditions, we added the followings in discussion part “Although our search found a few case reports from LMIC about fatal COVID-19 cases in children with malnutrition, none of the study extracted in our review investigated the impact of malnutrition on the severe COVID-19 outcomes in a population level.” (line 313-318) This area obviously needs further research.

We also removed a few sentences in a paragraph of CFR discussion to make it succinct based on your suggestions.

Style points:
Line 94: why "thus"? “Thus” was removed
Line 147: missing period after "Bank" Period was added
Line 168: Sentence is unclear and needs rewriting. Note that sometimes data are plural in the manuscript and sometime singular. The former are preferable, but need to be consistent throughout. This was reworded. “National pediatric populations by age for all countries were obtained from data in United Nations”
Line 185: What are "proportional outcomes"? Proportional was removed
Line 192: Representative not representatives “s” was removed
Line 231: "that of" should be deleted. deleted
Line 234: Age-specific number of fatality is not an accepted term. Replaced with “age-specific deaths”
Line 244 – " data of" should be: data regarding or data pertaining to Replaced with “data regarding”
Lines 250-253: Sentence needs to be rewritten for clarity. This sentence was reworded and moved to method section “In order to ensure data accurately reflected the time frame between searches 2 and 3, we excluded countries with CDC COVID-19 levels 2 to 4 (moderate, high, very high transmission) that had not updated their national reports for more than 2 months prior to December 7. These national reports were evaluated in the sensitivity analysis.”
Line 297: tests not test. Following this there are several compound words missing hyphens serologically –diagnosed PCR-diagnoses, age-specific. Corrected
Table 1 – Date should be added to the heading (to 31.08.2020). Added
Reference 13 in Supplement 2 – Israel is misspelled. (Isreal). Corrected

Reviewer #2: I would like to thank the authors for the immense work they did to conduct this systematic review. The topic of the review is obviously very important.

My comments:

Abstract:
Methods: To evaluate the magnitude of global ACUTE Covid-19 death, Acute does not seem correct, since not all PCR positive cases are necessary acute. They might also have been tested because were close contact of a case and were actually asymptomatic.
The number of reviewed articles and reports should be in the results not in the methods.
Since the Authors in the methods section report that only the first search round was performed on the countries national websites, I would suggest to remove the 11 languages from the abstract. Having the time period of the search in here would be very useful.
Conclusions: We knew that the COVID-19 an cause fatal disease in children, both in HIC and LMIC, and I would not say that should be the main conclusion of your study.

We agree with your point. “Acute” was removed.

The number of reviewed articles and reports were moved to Result section.

11 language and 16 databases were removed.

The date of search was added in abstract.

Conclusion was modified to “there may be a larger impact of pediatric COVID-19 fatality in LMICs compared to HICs”

Introduction
Line 75 – the authors should update the references reported. New reports with child mortality are available
<https://www.cdc.gov/mmwr/volumes/69/wr/pdfs/mm6937e4-H.pdf>
<https://services.aap.org/en/pages/2019-novel-coronavirus-covid-19-infections/children-and-covid-19-state-level-data-report/>
<https://www.thelancet.com/journals/lanchi/article/PIIS2352-4642(20)30177-2/fulltext>
These references were added in introduction.

Row 79 – number of cases is also dependent on the size of the population. “the size of population” was also added.
Row 80-83 – This seems more methods than introduction. These sentences were moved to methods.

Methods
Line 90-91 – Why primary case definition instead of just case definition? Corrected to “case definition”
Line 93-96 – What do you mean with POST-infectious multi-system inflammatory syndrome? What if the child had MIS-C during the hospitalization? I think the syndrome as such in COVID pediatric patients was not described from the beginning of the pandemic, therefore some of the children included in the review might have had it but was not clearly stated as such. How can the authors be sure that none of the included children had it?

We agree that MISC cases may not be fully excluded, especially in the early phase of pandemic. “Post-infectious” was removed. The potential inclusion of MIS-C cases in some country’s data has been included as a limitation.

Results
Line 207-209 – I am not sure I understand here the difference between countries or territories and then countries again. Could you please explain this sentence?

Because we used the country definition of World Bank, some are not independent countries, but “territory”, such as Taiwan, Gaum, and some of Caribbean or Pacific island countries. We believe this World Bank definition is better than United Nations definition, which just includes independent countries, because the outbreak situation in France vs French Polynesia or USA vs Guam is very different. However, we also agree that using “territory” in our manuscript is a bit confusing. So, in our article, we switched to use the word “county” throughout our manuscript.
Line 215 – Instead of global I would suggest to use “reported” deaths Switched to reported
Line 225 – I would suggest to use estimated CFR and ICU admission rate instead of global Switched to estimated
Line 250-252 – This seem to be more appropriate in the Methods part

The sensitivity analysis was explained in Methods part, and the sentence in result part was switched to “The result of one-way sensitivity is presented in Supplemental Table 6).”

Discussion
I find the discussion to be a little redundant, with a lot or results repetitions. Would prefer more reflections on the results and their interpretation. Like an interpretation of the results in figure 3A between UpperMIC and LIC.
Would also suggest to list the limitations of the study in a more ordered way instead of just spreading in the text.
The sentences about result description were removed to avoid repetitions of result.

Low fatality/capita (but still high CFR) in LIC was discussed at the end of the first paragraph of discussion part. (Line 282-285)

Limitations are now listed in one paragraph (Line 298-318).

Line 267-268 – I am not sure this is the case for Covid. Since there was high concern about the limited capacity of low middle income countries to perform PRC test, there has been high attention on the number of deaths, in all age groups so I would not conclude that there might have been many Covid related deaths which have been missed in the pandemic.
Line 269-271 – As above, there has been high attention to the number of deaths in low middle income countries, therefore I would not suggest that the prevalence of pediatric COVID-19 death is likely to be much higher
Line 293-295 – This is probably more true in LMIC than in HIC
Based on your suggestion, we switched to “whether undercounting deaths in LMIC is also applied to COVID-19 deaths is unknown” and removed the sentences about our assumption of undercounting of COVID-19 deaths in LMIC.

Conclusion
Throughout the manuscript the authors talk about impact, burden and severe outcomes. Would be good to be consistent and align the terminology. Line 326-335 – This sound more a continuation (and repetition) of the discussion more than a conclusion.

Modified to use “impact” throughout the manuscript. The conclusion section was shortened to avoid repetition as suggested.

Figures
Table 1. I would suggest to put this in the supplement. Would also say to include the number of deaths and ICU admissions for each country
Figure 1. The resolution of the image is not good enough for me to read the content

Table 1 was moved to supplement, and the number of deaths and ICU admissions for each country were added. Figure 1 is provided with a new word file.
