# Supplementary material for: The differential impact of pediatric COVID-19 between high-income countries and low- and middle-income countries: A systematic review of fatality and ICU admission in children worldwide
Source: PLoS One. 2021 Jan 29;16(1):e0246326. doi: 10.1371/journal.pone.0246326 (PMC7845974; doi:10.1371/journal.pone.0246326)
Supplement: Attachment — Submitted filename: Response to reviewer .docx [file pone.0246326.s013.docx]

Thank you very much again for your time to review our revised manuscript. We sincerely appreciate editor and reviewers’ time to timely process and review our article.

Reviewer #1: The authors have taken careful note of the reviewers' comments and the manuscript is updated and improved. It could still benefit from some style and linguistic improvements eg "There is a geographical disparity about countries which contributed to our study." - this sentence is unclear - does this mean not all geographic regions were equally presented? Are the authors referring to the quality of data which varies according to geography. The paper should be carefully reread for clarity. The conclusions of the study perhaps should add a suggestion that given the lack of uniform testing strategies among children across the globe, careful temporal and geographic comparisons of all-cause mortality among children should be undertaken during the COVID-19 pandemic and periods before and after, in order to discern the possible extent of undiagnosed SARs-Cov2-related mortality.

Thank you for the point. We meant the number (or proportion) of countries reporting national data in children differed by geographical region. The sentence was switched to “Not all geographic regions equally reported national data in children” as suggested.

Based on your suggestion, the sentence “Given the lack of uniform testing strategies among children across the globe, temporal and geographic comparisons among children should be carefully undertaken in order to discern the possible extent of undiagnosed SARS-CoV-2-related deaths” was added in conclusion

Reviewer #2: I thank the authors for addressing all my comments and the extra work done in updating the research.
I find that the manuscript reads better now.
Minor suggestion:
- Discussion – (Line 378-9): “While this study focuses on acute COVID-19, it is possible that some country’s data may include cases of MIS-C.” Here I would suggest rephrasing in “While this study excluded MIS-C cases, it is possible that some country’s data may include these cases.”

Thank you for the advice. Rephrased as suggested “While this study excluded known MIS-C cases, it is possible that some country’s data may include these cases.”
